# Comparison of the Efficiency of B–O and B–C Bond Formation Pathways in Borane-Catalyzed Carbene Transfer Reactions Using α-Diazocarbonyl Precursors: A Combined Density Functional Theory and Machine Learning Study

**DOI:** 10.1021/acscatal.4c03368

**Published:** 2024-09-16

**Authors:** Kaveh Farshadfar, Kari Laasonen

**Affiliations:** Department of Chemistry and Material Science, School of chemical Engineering, Aalto University, 02150 Espoo, Finland

**Keywords:** α-diazocarbonyl, carbene transfer, tris(pentafluorophenyl)borane, boron, catalysis, DFT, machine learning

## Abstract

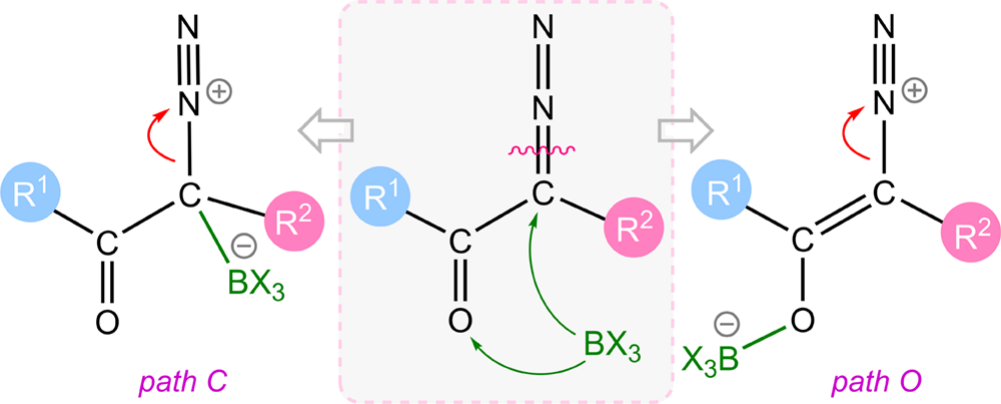

Lewis acidic boranes, especially tris(pentafluorophenyl)borane [B(C_6_F_5_)_3_], have emerged as metal-free catalysts for carbene transfer reactions of α-diazocarbonyl compounds in a variety of functionalization reactions. The established mechanism for how borane facilitates carbene generation for these compounds in the scientific community is based on the formation of a B–O (C=O) intermediate (*path O*). Herein, we report an extensive DFT study that challenges the notion of a ubiquitous *path O*, revealing that B–C(=N=N) bond formation (*path C*) for certain diazocarbonyl substrates proves to be the preferred pathway. This study elucidates, through the introduction of 22 various substituents on each side of the α-diazocarbonyl backbone, how the electron-donating and -withdrawing properties of substituents influence the competition between these B–O and B–C pathways. To elucidate the impact of the electronic features of diazo substrates on the competition between the O and C pathways in the studied dataset, we employed a machine learning approach based on the Random Forest model. This analysis revealed that substrates with higher electron density on the diazo-attached carbon, lower electron density on the carbonyl carbon, and more stable HOMO orbitals tend to proceed via *pathC*. Furthermore, this study not only demonstrates that borane efficiency in facilitating N_2_ release is greatly affected by the nature of substituents on both sides of the α-diazocarbonyl functionality but also shows that for some substrates, borane is incapable of catalyzing the release of molecular nitrogen.

## 1 Introduction

Trivalent boron compounds, possessing a vacant p-orbital that makes them effective Lewis acid catalysts, have long served as powerful tools in organic synthesis.^1,2^ Over the past decade, tris(pentafluorophenyl)borane, B(C_6_F_5_)_3_, has garnered significant attention for its catalytic function in activating α-diazocarbonyl compounds in carbene transfer reactions, enabling their broad application in cyclization, alkenylation, or X-H insertion reactions.^3−17^

**Scheme 1:**
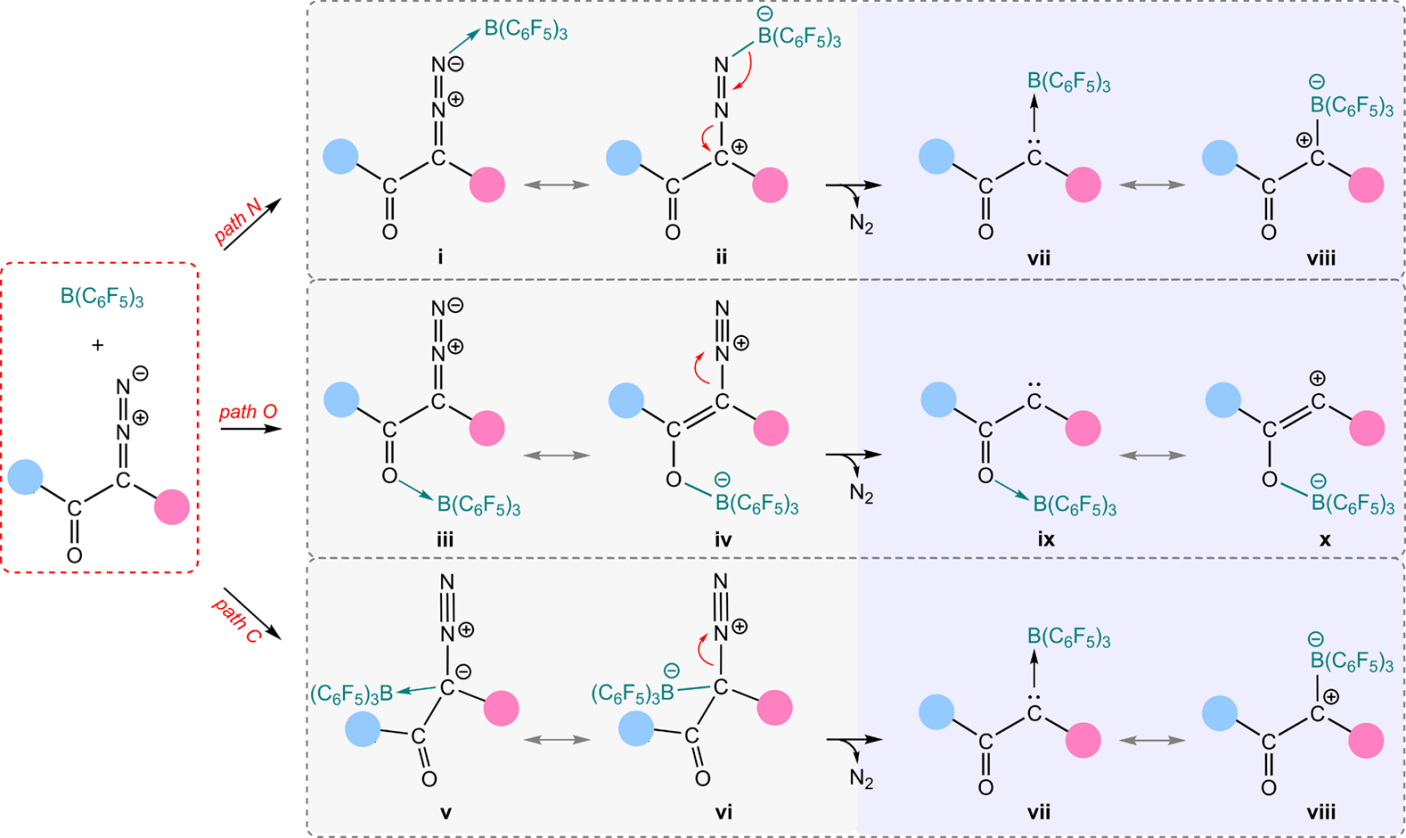
Possible Pathways for Activation of α-Diazocarbonyl Precursors for N_2_ Release Using B(C_6_F_5_)_3_ Red arrows indicate the direction of electron transfer leading to the subsequent structures.

Three plausible pathways can be envisioned for B(C_6_F_5_)_3_-catalyzed N_2_ release from α-diazocarbonyl compounds.^18^ As depicted in Scheme 1, borane may facilitate N_2_ release via the formation of B–N adduct, (path N), B–O adduct (*path O*), and B–C adduct (*path C*). The contribution of resonance structures **ii**, **iv**, and **vi**, which feature a weakened C–N bond, is expected to contribute to a decrease in the activation energy for N_2_ release. Notably, borane can also stabilize the resulting carbenes by delocalizing the electron density (Scheme 1). Density functional theory (DFT) studies have revealed that path N is not the preferred pathway for these borane-catalyzed transformations.^7,18^ The weak electron donation (from the sp orbital) of the nascent N_2_ molecule to the boron atom appears to be the underlying reason, preventing the stabilization of transition structures for N_2_ elimination as well as the ensuing N → B intermediates.

**Scheme 2:**
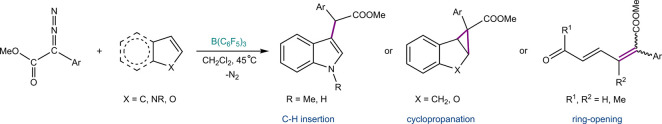
B(C_6_F_5_)_3_-Catalyzed Carbene Transfer Reactions Reported by Melen and Co-Workers

Recently, Melen and their co-workers^19^ conducted a DFT study to demonstrate that B(C_6_F_5_)_3_ can promote the release of N_2_ from α-diazocarbonyl compounds via B–O adduct formation in a series of carbene transfer reactions. According to their proposed mechanism, the borane-stabilized carbene intermediate **ix** (Scheme 1), which also exhibits carbocationic characteristics as evidenced by resonance contributor **x**, is poised for nucleophilic attack at the carbon atom. Consequently, this intermediate undergoes C–H insertion, cyclopropanation, and ring-opening reactions (Scheme 2). This proposed mechanism has been extensively cited in subsequent studies by them^20−23^ as well as by other research groups.^8−10,12,22−28^ Noteworthy that this pathway (*path O*) had also been proposed by Zhang et al. in a separate study.^18^ Nevertheless, our preliminary computations have indicated that *path O* may not invariably represent the preferred catalytic route for borane-catalyzed N_2_ elimination from α-diazocarbonyl precursors; instead, *path C* poses a potentially competitive alternative. Indeed, substituents on either side of the α-diazocarbonyl moiety exert distinct effects on the activation energies associated with these competing pathways.

Therefore, to establish the energy barriers for borane-catalyzed carbene formation from α-diazocarbonyl compounds via both *path O* and *path C*, we undertook comprehensive DFT studies at the SMD/M06-2X/def2-TZVP//SMD/M06-2*X*/6-31G(d) level of theory. Additionally, a machine learning (ML) approach was employed to analyze the DFT results and identify relevant chemical descriptors. This integrated approach has the potential to uncover previously unseen correlations and provide a deeper understanding of chemical transformations.

## 2 Results and Discussion

### 2.1 Scrutinous Look at the Mechanism of Carbene Generation from Phenyldiazoacetate

**Figure 1:**
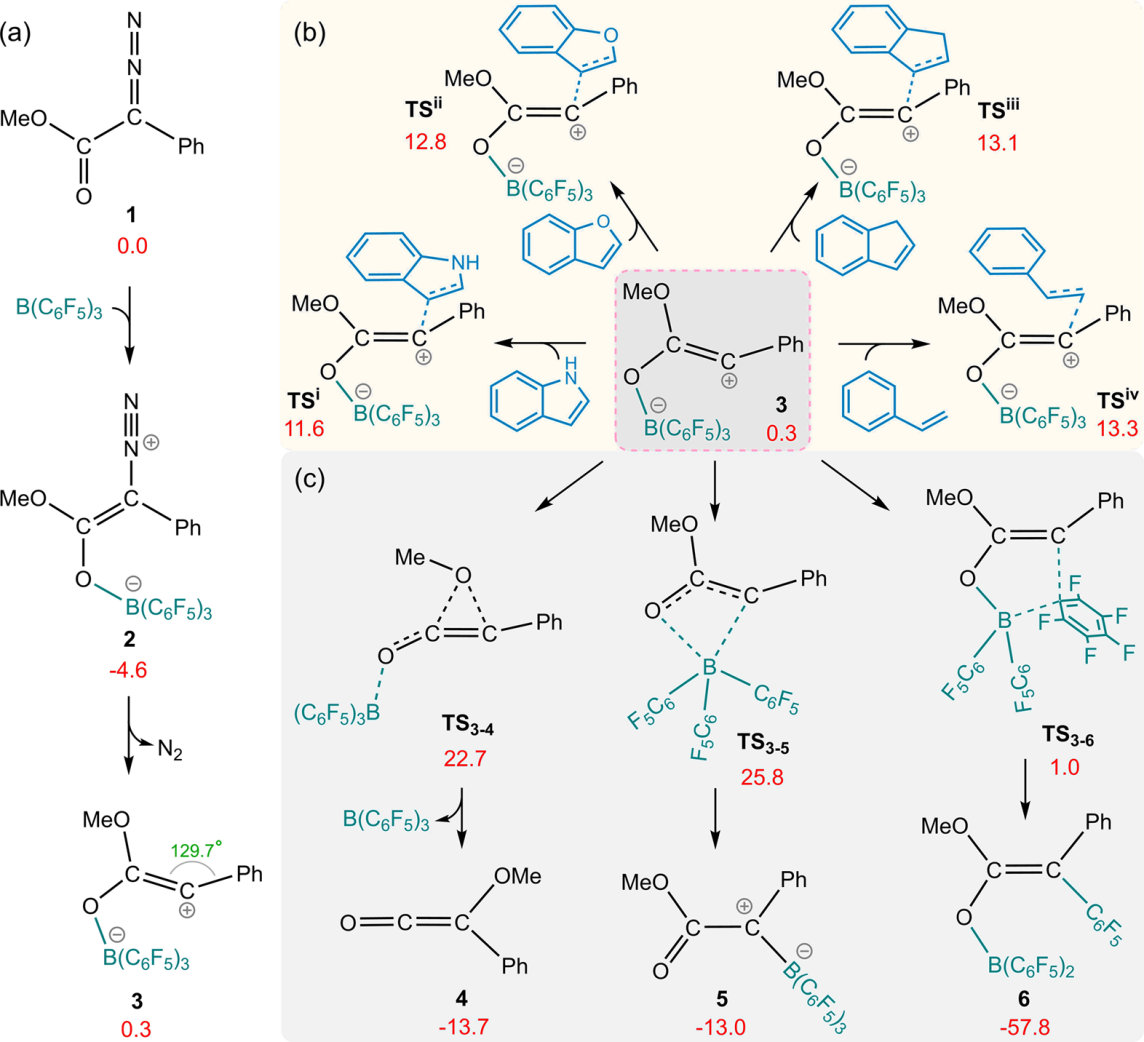
Calculated free energy for (a) N_2_ elimination of phenyldiazoacetate (**1**) in the presence of B(C_6_F_5_)_3_ via *path O*, (b) nucleophilic attack transition structures of indole, benzofuran, indene, and styrene, on the carbon center of **3**, and (c) plausible rearrangement of intermediate **3**. The relative free energies are given in kcal/mol, and the angle is annotated in green.

We undertook a scrutinizing investigation of the mechanistic aspects of the B(C_6_F_5_)_3_-catalyzed reaction of methyl phenyldiazoacetate (**1**) with nucleophiles indole, benzofuran, indene, and styrene, as reported by Ariafard and co-workers.^19^ The relative free energies for the B–C adduct (**2**) and borane-stabilized carbene (**3**) were calculated to be −4.6 and 0.3 kcal/mol, respectively (Figure 1a). Both the carbocation and carbene resonance forms of intermediate **3** possess a vacant p orbital perpendicular to the molecular plane, rendering it susceptible to nucleophilic attack.

Our DFT calculations show that the relative free energy barriers for the addition of indole, benzofuran, indene, and styrene to **3** are 11.6, 12.8, 13.1, and 13.3 kcal/mol, respectively (Figure 1b). However, the inherent carbenic-carbocationic character of intermediate **3** renders it highly reactive, potentially leading to intramolecular rearrangement before nucleophilic attack. The methoxy group might undergo migration to the formally positively charged carbon in a Wolff rearrangement. Although DFT calculations for the **3** → **4** rearrangement show that this migration is exergonic, it exhibits a high free energy barrier of 27.3 (22.7 – (−4.6)) kcal/mol. Similarly, B(C_6_F_5_)_3_ migration from oxygen to carbon to form zwitterion **5,** through **TS_3–5_**, requires an activation energy of 30.4 (25.8 – (−4.6)) kcal/mol. However, our calculations indicate that intermediate **3** readily undergoes C_6_F_5_ migration to the positively charged carbon atom via **TS_3–6_** (Δ*G*^‡^ = 1 – (−4.6) = 5.6 kcal/mol). The generated intermediate (**6**) is thermodynamically highly stable, suggesting that this step is not reversible. This essentially renders the experimental product unattainable, leading us to consider the possibility that the transformation does not proceed via the proposed mechanism.

The borane-catalytic mechanism, initiated by the addition of borane to carbon possessing diazo functionality (Scheme 1—*path C*), has rarely been considered. This mechanism was explored by Zhang et al.^18^ as a possible pathway for borane-catalyzed carbene transfer reactions. However, their calculations did not identify it as the preferred pathway. Nonetheless, in 2023, Nemoto and co-workers^7^ demonstrated the functional potential of *path C* in facilitating N_2_ release from specific α-diazocarbonyl compounds.

**Figure 2:**
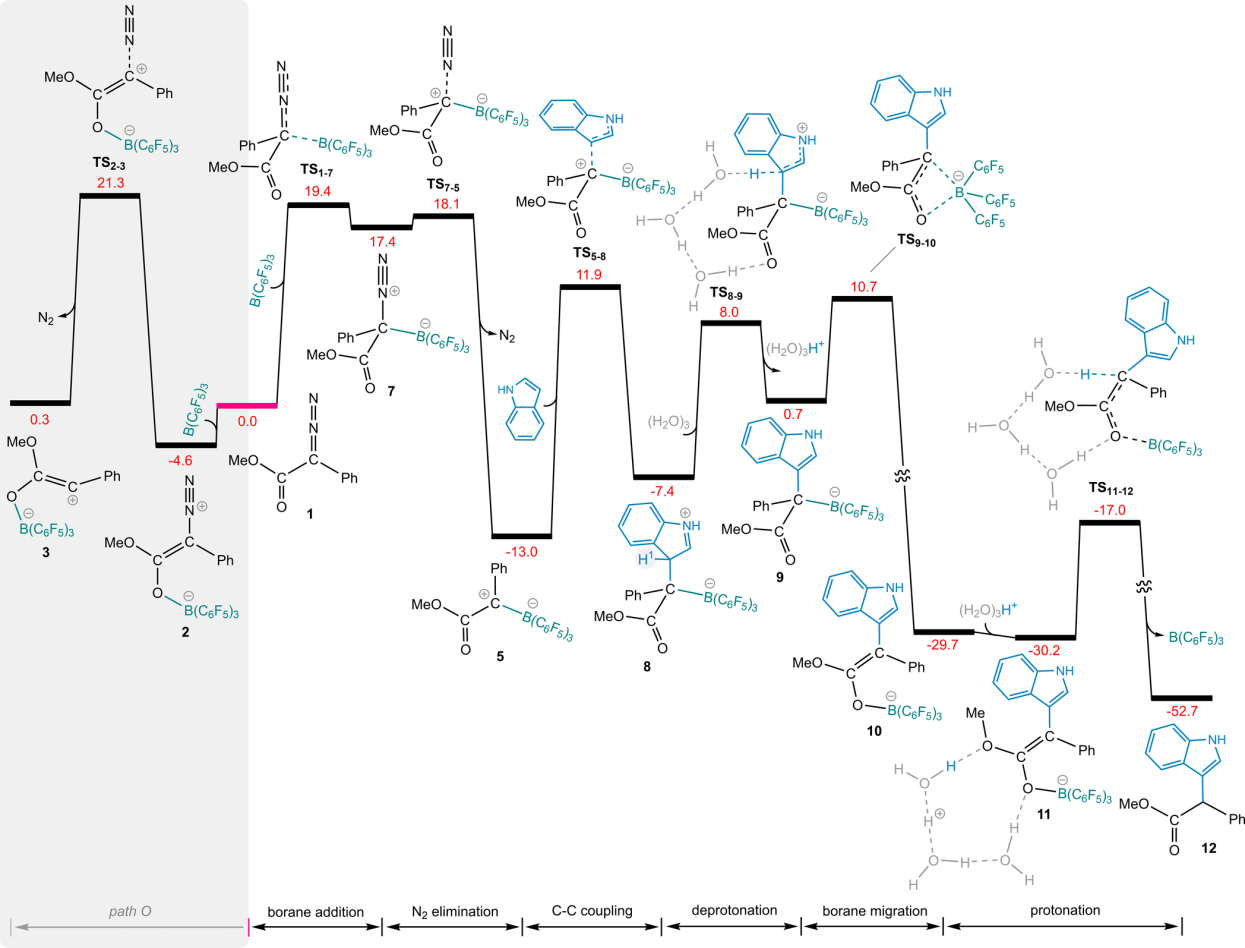
DFT-computed pathways for B(C_6_F_5_)_3_-catalyzed N_2_ release of phenyldiazoacetate (**1**), followed by reaction with the indole substrate. The relative free energies are given in kcal/mol.

We therefore calculated the free energy profile for *path C* of the aforementioned reaction, starting with the binding of B(C_6_F_5_)_3_ to the carbon atom bearing the diazo functionality. Figure 2 depicts the results of our DFT calculations for this pathway. Following the formation of the B–C adduct (**7**), N_2_ is released via transition state **TS**_**7**–**5**_, generating borane-stabilized carbene **5**. Both transition structures **TS**_**1**–**7**_ and **TS**_**7**–**5**_ lie at lower free energy levels compared to transition structure **TS**_**2**–**3**_, making *path C* more favorable in competition with *path O*. Subsequently, to explore nucleophilic reactions arising from intermediate **5** (Scheme 2), we calculated the free energy profile for its reaction with indole as a representative nucleophile. Intermediate **5** can adopt a zwitterionic resonance structure featuring a positively charged carbon atom and a negatively charged boron atom. The carbon atom in this resonance contributor, as well as in the carbenic resonance structure (**vii** - Scheme 1), is susceptible to nucleophilic attack. The addition of indole to intermediate **5** proceeds via transition structure **TS**_**5**–**8**_, requiring an activation energy of 24.9 kcal/mol (the free energy difference between **TS**_**5**–**8**_ and **5**) and leads to the formation of species **8** in an endergonic manner. Hydrogen atom 1 in intermediate **8** is remarkably acidic, readily undergoing deprotonation, even with mild bases such as indole or adventitious water. Adventitious water present in solvents such as dichloromethane can act as a proton shuttle. Instances of water functioning as a proton shuttle in nonaqueous solvents have been documented.^29−32^ Proton transfer was considered to be mediated by water molecules in this step. In both aqueous and nonaqueous solvents, water molecules can form clusters through hydrogen bonding, and it is well-documented that clusters of three water molecules (H_2_O)_3_ can participate in chemical transformations.^33−36^ The calculated free energy barrier for water-assisted proton elimination from **8** to yield **9** in an uphill process via the transition structure **TS**_**8**–**9**_ is 21.0 kcal/mol relative to **5**. Intermediate **9** undergoes B(C_6_F_5_)_3_ migration from carbon to oxygen, leading to a substantial stabilization of 30.4 kcal/mol stabilization. The resulting species **10** can form ion-pair adduct **11** by establishing hydrogen bonds with (H_2_O)_3_H^+^. Proton transfer from (H_2_O)_3_H^+^ to the carbon atom, overcoming an energy barrier of 13.2 kcal/mol, yields the final product **12**.

### 2.2 Does *Path C* Always Take Priority over *Path O*?

**Scheme 3:**
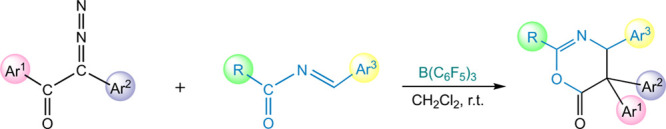
B(C_6_F_5_)_3_-Catalyzed Carbene Transfer Reactions Reported by Stephan and Co-Workers

To address whether all carbene transfer reactions from α-diazocarbonyl precursors occur through *path C* or not, we investigated pathways O and C for a nonester α-diazocarbonyl compound. Stephan and co-workers recently described a B(C_6_F_5_)_3_-catalyzed Wolff rearrangement (Scheme 3).^5^

**Figure 3:**
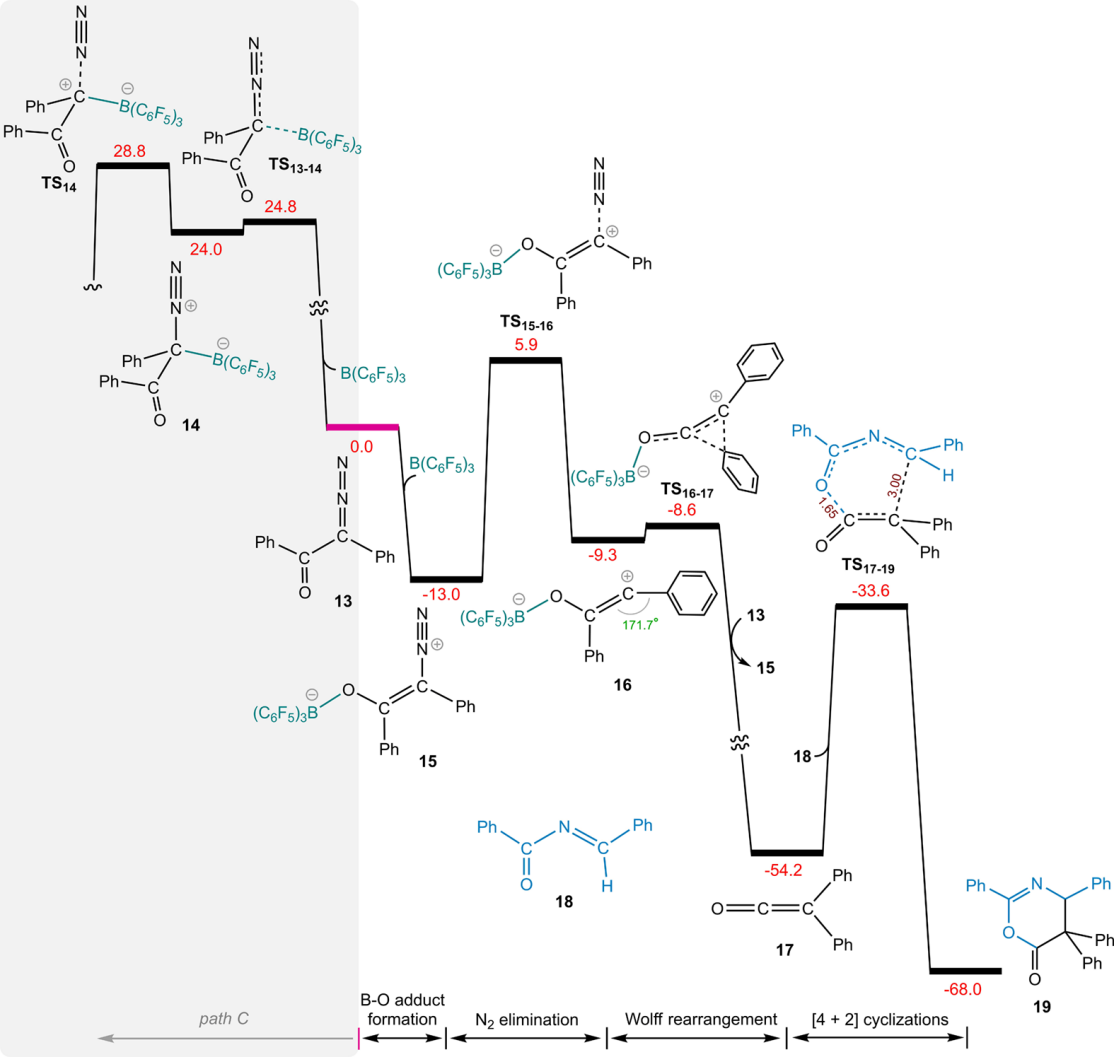
DFT-computed pathways for B(C_6_F_5_)_3_-catalyzed N_2_ release of 2-diazo-1,2-diphenylmethane-1-one (**12**), followed by Wolff rearrangement and [4 + 2] cyclization. The relative free energies are given in kcal/mol. The selected distances (Å) are annotated in brown, and the angle is shown in green.

Figure 3 illustrates the calculated energy profile for the formation of ketene from 2-diazo-1,2-diphenylethan-1-one (**13**). Interestingly, the initial B–O adduct (**15**) for this diazo substrate is significantly more stable compared to that of adduct **2**. This heightened stability makes it difficult for *path C* to proceed. Our results reveal a striking difference in the N_2_ dissociation pathways for α-diazoester versus α-diazoketones. While *path C* is preferred for the former (Figure 2), *path O* emerges as the favorable route for the latter, as evidenced by **TS**_**15**–**16**_ being 22.9 (5.9–28.8) kcal/mol more stable than **TS**_**14**_ (Figure 3). The B(C_6_F_5_)_3_-stabilized carbene **16** undergoes facile phenyl migration, affording ketene **17** in a highly exergonic fashion. The [4 + 2] cycloaddition of **18** and ketene proceeds through transition structure **TS**_**17**–**19**_ to yield the final product (**19**), requiring a moderate activation energy of 20.6 kcal/mol.

To further validate our findings, we recalculated the key structures of the two reaction pathways illustrated in Figures 2 and 3 using additional DFT methods, including the M06, M06-D3, and wB97XD functionals. These computed energies are summarized in Table S1 of the Supporting Information.

#### 2.2.1 *Path C* vs *Path O*

To elucidate the influence of substituent positioning on α-diazocarbonyl precursors, we investigated their preference for *path O* or *path C* in the dissociation of N_2_. To this end, we opted for cost-effective computations using the smaller molecule BCl_3_ instead of B(C_6_F_5_)_3_. BCl_3_ is a slightly stronger Lewis acid than B(C_6_F_5_)_3_.^37^ Although neither of these boranes protects the boron atom with their substitution, the effects of steric hindrance may vary depending on the substrates. Additionally, interactions such as CH−π and π–π stacking may occur in certain cases involving B(C_6_F_5_)_3_. Comparative energy profiles for the two different N_2_ elimination pathways for the two substrates discussed above (**1** and **13**), in the presence of BCl_3_, are provided in Figure S1. This analysis indicates that while changing the borane leads to somewhat different energy barriers, it does not affect the pathway preferences for these substrates. Nevertheless, in cases where the energy barrier difference between two pathways is small, the type of borane might be determinative. Additionally, employing BCl_3_ in this study could potentially draw more attention to this borane from the synthetic chemistry community.

**Scheme 4:**
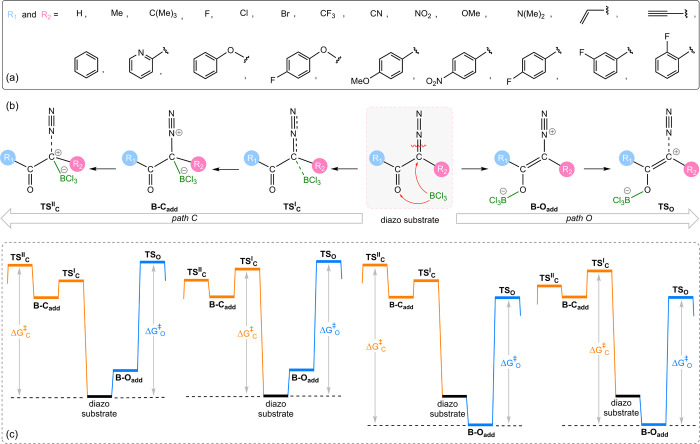
(a) R_1_ and R_2_ Substituents Employed To Generate Our α-Diazocarbonyl Dataset, (b) Two Catalytic Pathways O and C, (c) Schematic Illustrating the Four Possible Scenarios for Calculating Δ*G*_O_^‡^ and Δ*G*_C_^‡^ for Each Diazo Substrate

Accordingly, we introduced 22 different substituents on both sides of the α-diazocarbonyl backbone (Scheme 4a). The substitutions encompassed a wide range of electron-donating and -withdrawing groups. This resulted in 484 distinct samples. Notably, the substrate in which both R_1_ and R_2_ are substituted with H underwent rearrangement during N_2_ release via *path O* and was therefore excluded from the dataset. For each sample, key structures involved in the two potential mechanisms for N_2_ elimination via pathways O and C were identified. These structures include the B–O adduct (**B**–**O**_**add**_), the transition state for N_2_ release via *path O* (**TS**_**O**_), and two transition states for C–O adduct formation (**TS**_**C**_^I^) and subsequent N_2_ release (**TS**_**C**_^II^) via *path C* (Scheme 4b). We also identified transition structures in the absence of BCl_3_ to assess the catalyst efficiency for each substrate. The activation energy for each pathway was obtained by calculating the difference in free energy between the most stable species (either the initial diazo compound or the B–O adduct) and the transition state energy for that pathway (Scheme 4c). It is worth noting that for *path C*, some substrates exhibit a higher energy barrier for **TS**_**C**_^II^ (N_2_ elimination) compared to **TS**_**C**_^I^ (B–O bond formation), while others demonstrate a higher barrier for **TS**_**C**_^I^. Moreover, for a subset of diazo substrates, N_2_ elimination occurs concomitantly with B–C bond formation, thus circumventing the formation of a B–C adduct as a stationary point.

**Figure 4:**
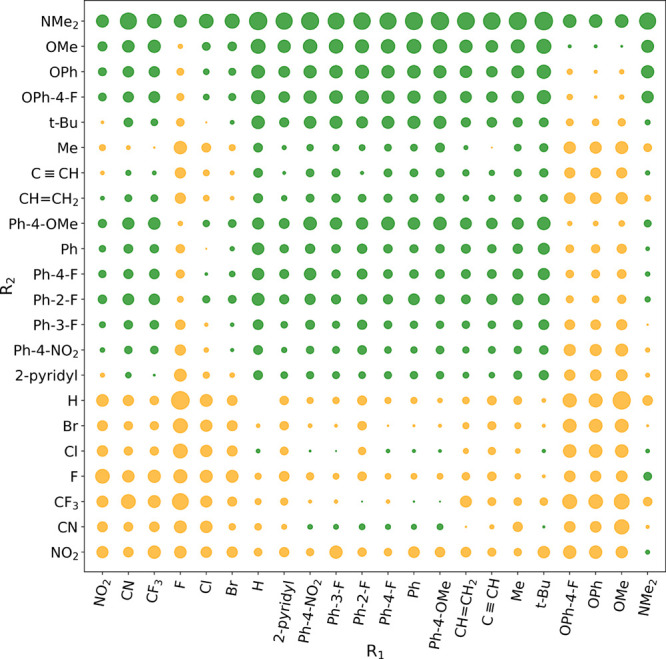
Effect of the R_1_ and R_2_ substituents on ΔΔ*G* (Δ*G*_C_^‡^ – Δ*G*_O_^‡^). Positive ΔΔ*G* values indicate the superiority of *path O* (represented by green circles), while negative ΔΔ*G* values suggest greater favorability for *path C* (represented by orange circles). The diameter of each circle is proportional to the magnitude of ΔΔ*G*. Small circles denote a minor energy difference in the activation barrier values between the two pathways.

Figures S2 and S3 illustrate the catalytic efficacy of BCl_3_ in pathways O and C for each sample, and Figure S4 complements this analysis by depicting the overall catalytic efficiency of BCl_3_. Our findings suggest that in certain samples the involvement of BCl_3_ results in an elevation of the activation energy required for N_2_ elimination, causing it to fail to function as a catalyst in either of the two pathways. Our entire dataset is presented in Table S3, and a graphical representation of the preferred reaction pathway for each substrate is provided in Figure 4.

The results (Figure 4 and Tables S2 and S3) indicate that methoxy and phenoxy functional groups at the R_1_ position favor *path C*, but the electron-donating ability of R_2_ modulates this preference. Accordingly, when the R_2_ group is NO_2_, CF_3_, or CN (with R_1_ being OMe, OPh, or OPh-4-F), *path C* is more favored compared to *path O*. However, as the electron-donating strength of R_2_ increases, the difference between Δ*G*_O_^‡^ and Δ*G*_C_^‡^ diminishes, and for a strong R_2_ electron-donor like NMe_2_, *path O* becomes preferred. Supporting this trend, the preference for *path C* for this class of samples follows the order: 4-methoxyphenyl < phenyl < 4-nitrophenyl for R_2_. Furthermore, weaker π-donating groups such as aromatic rings, vinyl groups, and ethynyl groups at the R_1_ position generally favor *path O* when R_2_ is a group of the same type or a strong σ-donor like tert-butyl. It is also worth noting that halogens and strong electron-withdrawing groups such as NO_2_, CF_3_, and CN at the R_2_ position, combined with a set of R_1_ groups including halogens, NO_2_, CF_3_, CN, methoxy, and phenoxy, favor *path C*. However, in most cases, π-donors at the R_2_ position select *path C* unless R_1_ is methoxy, phenoxy, or fluorine.

#### 2.2.2 Most Important Parameters

##### 2.2.2.1 B–O Adduct Stability

Some examples of data derived from our calculations, with more details, are showcased in Table 1 along with additional examples in Table S2. The significance of the stability of the B–O adduct emerges as a pivotal determinant of pathway selectivity. When the B–O adduct exhibits high thermodynamic stability, the reaction *path C* becomes challenging, even if there is a small energy difference between its transition structures and the starting materials due to the elevated energy difference between the transition structures and the resting state of the catalyst (Figure S5).

**Table 1:**
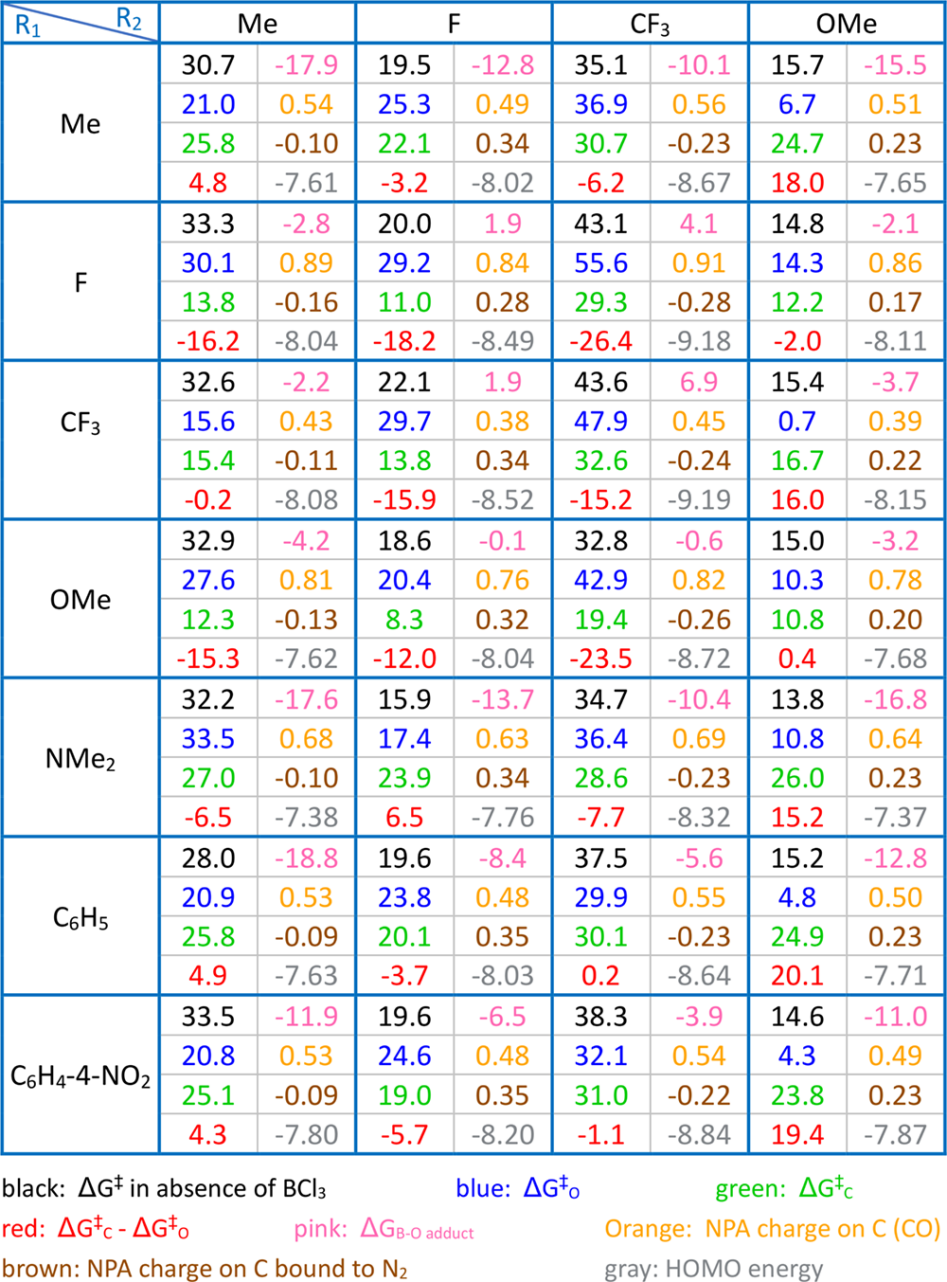
Dataset Examples: i.e., Free Energy Barrier (in kcal/mol) of N_2_ Release (without BCl_3_, Pathways C and O), Δ*G*_C_^‡^ – Δ*G*_O_^‡^, Relative Stability of the B–O Adduct (in kcal/mol), NPA Charge on Backbone Carbons, and HOMO Energy Level (in eV) for Each Diazo Substrate

R_1_ electron-donating substituents augment the basicity of the carbonyl oxygen, thereby strengthening the B–O bond. This effect is exemplified by methyl, amine, and phenyl substituents. For instance, when R_1_ and R_2_ are methyl groups, the highest transition structure for *path C* displays an energy difference of merely 8.9 kcal/mol relative to the starting materials. Nevertheless, due to the B–O adduct’s relative free energy of −17.9 kcal/mol, the free energy barrier for *path C* amounts to 25.8 (8.9 + 17.9) kcal/mol.

**Figure 5:**
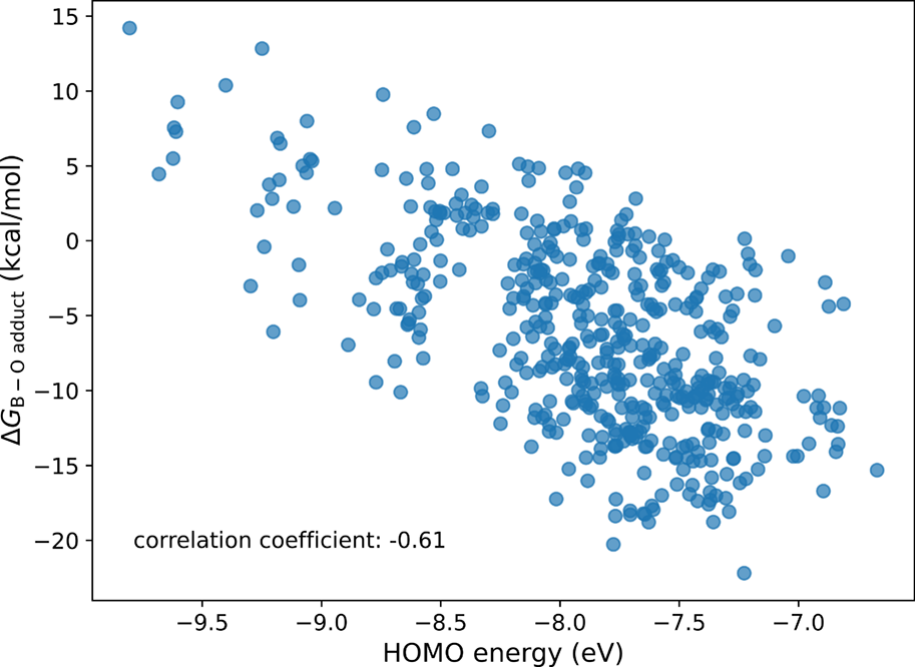
Scatter plot of the HOMO energy levels versus the stability of the B–O adducts.

Our findings indicate a notable correlation between the energy levels of the highest occupied molecular orbital (HOMO) and the stability of the B–O adducts (Figure 5). The HOMO energy level can serve as an indicator of the electron-withdrawing and -donating properties of the substituents on either side of the diazo functionality. On the other hand, although the p_*x*_ lone pair electrons on oxygen, which interact with the vacant boron orbital, are located at different underlying layers in HOMO-n for various diazo molecules, a correlation exists between the energy levels of the HOMO and those of oxygen’s p_*x*_ orbital (for further details, see the Supporting Information.). The elevated energy of the p_*x*_ orbital of oxygen, which is predominantly reflected in the higher-energy HOMO, leads to a stronger interaction with the vacant boron orbital, resulting in a more stable B–O adduct.

Overall, the electron-donating properties of both substituents elevate the HOMO energy level, which increases the interaction between borane and the diazo substrate, thereby facilitating *path O*.

##### 2.2.2.2 B–C Adduct Stability

Samples with R_1_ and R_2_ substituents exhibiting σ-donating (^*t*^Bu), σ-withdrawing (CF_3_), and π-donating (OMe) characteristics were selected to investigate the stability of the B–C adducts and the ease of their formation. It should be noted that the free energy values for **TS**_**C**_^I^, **B–O**_**add**_, and **TS**_**C**_^II^ were calculated relative to the starting materials (the corresponding diazo substrate and BCl_3_).

**Figure 6:**
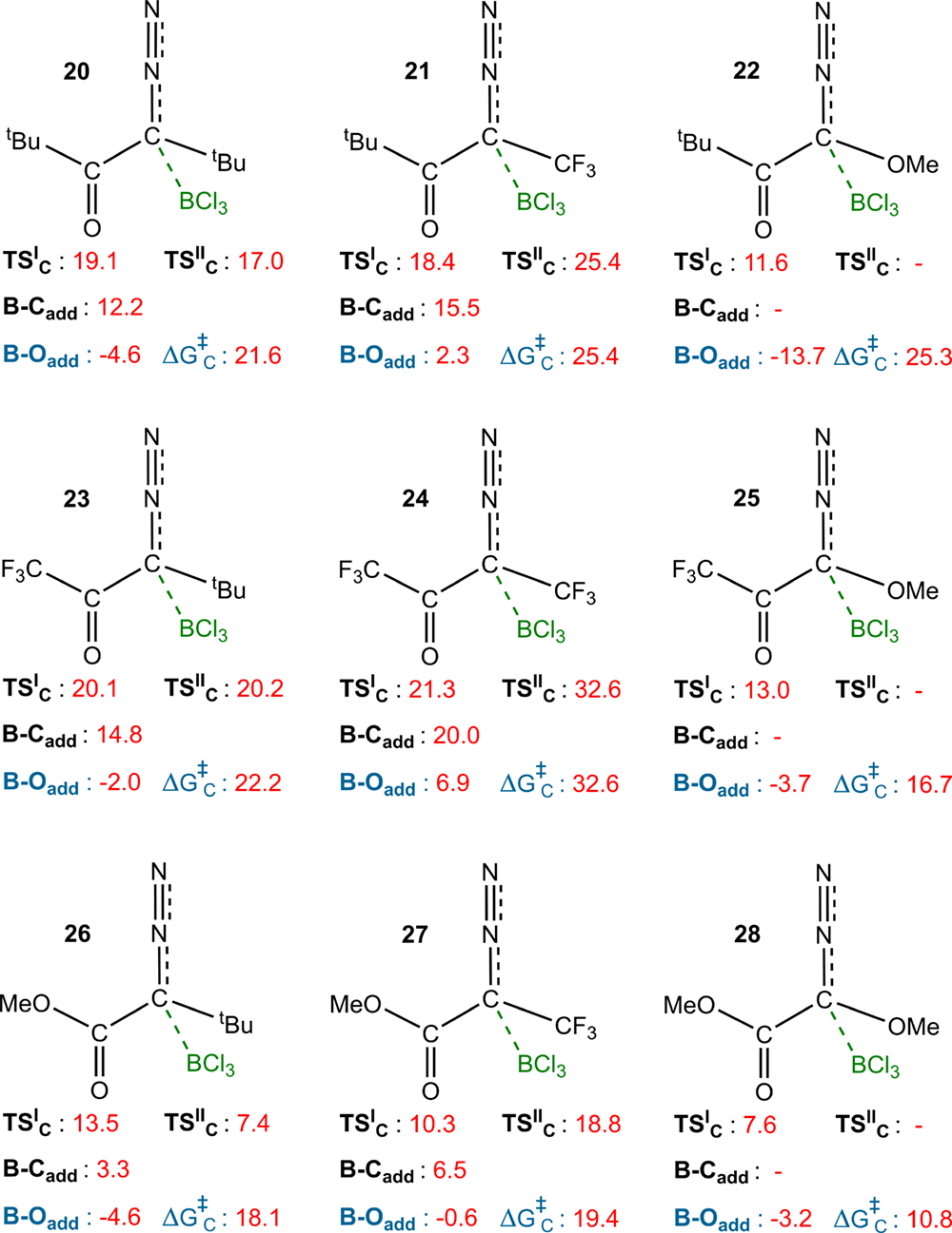
Effect of changing R_1_ and R_2_ from ^*t*^Bu to CF_3_ and OMe on **TS**_**C**_^I^ and Δ*G*^‡^_C_.

As shown in Figure 6, greater electron donation by R_2_ leads to a more stable B–C adduct as carbon can more effectively donate electron density to the boron center. For structures in which R_2_ is OMe, no local minimum for the B–C adduct was found. In these cases, the N_2_ molecule is eliminated during the formation of the boron–carbon bond, facilitated by electron donation from OMe to the nascent carbene p_*z*_ orbital. For these compounds, the relative energy of **TS**_**C**_^I^ is significantly lower compared to those with R_2_ = ^*t*^Bu or CF_3_. However, in a sample such as **22**, the lower energy of the B–O adduct ultimately leads to a higher Δ*G*^‡^_C_. These results indicate that in addition to R_2_, the electron-donating ability of R_1_ also contributes to the stability of the B–C adduct, with this effect being more pronounced for π-donating properties compared to σ-donating properties.

##### 2.2.2.3 Thermodynamic Stability of the Borane-Stabilized Carbenes

**Figure 7:**
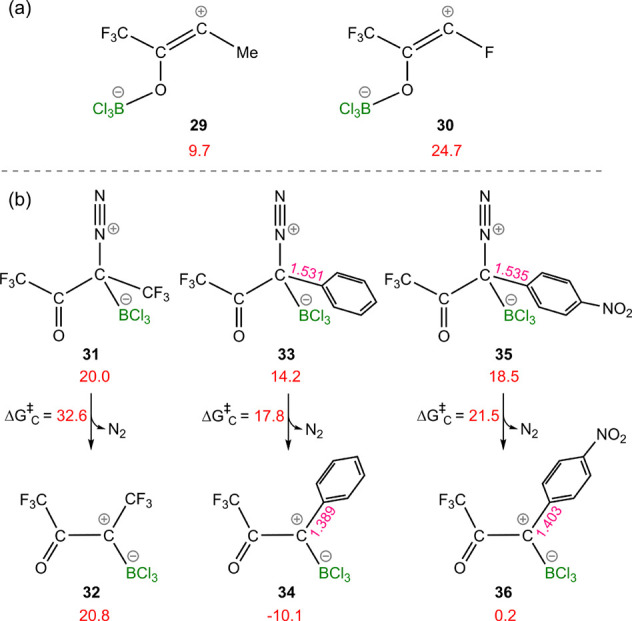
(a) Impact of borane-stabilized carbene stability on the energy barrier of *path C*. (b) Comparison of the effect of substituents on the thermodynamic stability of borane-stabilized carbenes on *path O*. The relative free energies are given in kcal/mol, and selected bond lengths are given in Å.

For an α-diazocarbonyl compound possessing R_1_ = CF_3_ and R_2_ = Me, the activation energy for N_2_ elimination in *path O* is 15.6 kcal/mol, whereas when R_2_ is substituted with F, the activation energy increases to 29.7 kcal/mol (Table 1). This is likely caused by the thermodynamic stability of the ensuing species after N_2_ release, which, to some extent, is reflected in the transition structures. The relative free energy for species **29** is 9.7 kcal/mol, while for species **30**, it is calculated to be 24.7 kcal/mol (Figure 7a). The instability of the ensuing intermediate is attributable to the destabilizing effect of fluorine on zwitterion **30**, which reduces the contribution of resonance structure **x** (Scheme 1). Substitution of the F moiety with a CF_3_ group further accentuates this effect, resulting in a significant elevation of the energy barrier to 47.9 kcal/mol (Table 1).

While the stability of the B–C adduct undeniably affects the activation barrier for *path C*, the stability of the ensuing intermediate after N_2_ release also exerts a substantial impact on the magnitude of **TS**_**C**_^II^. A better π-donor R_2_ can interact with the antibonding orbital of the N–C bond, thus lowering the activation energy for N_2_ elimination (**TS**_**C**_^II^). The magnitude of this stabilization can be reflected by the intermediate. This is exemplified by B–C adduct **35** (Figure 7b), which exhibits a relative free energy close to that of **31** but has a considerably lower Δ*G*^‡^_C_. This discrepancy can be seen in the stability of their subsequent intermediates. Species **36** was found to be 20.6 kcal/mol lower in energy than **32**. This enhanced stability is clearly reflected in the activation energy for N_2_ elimination, differing by 11.1 kcal/mol. The 4-nitrophenyl substituent, acting as a π-donor by delocalizing the positive charge in **36**, stabilizes it. As depicted in Figure 7b, the phenyl group exerts a more pronounced stabilizing effect than the 4-nitrophenyl group, evidenced by the shorter carbon–carbon bond distance in **34** compared to **36**.

##### 2.2.2.4 π-Donating Capability of R_1_ Substituents

**Figure 8:**
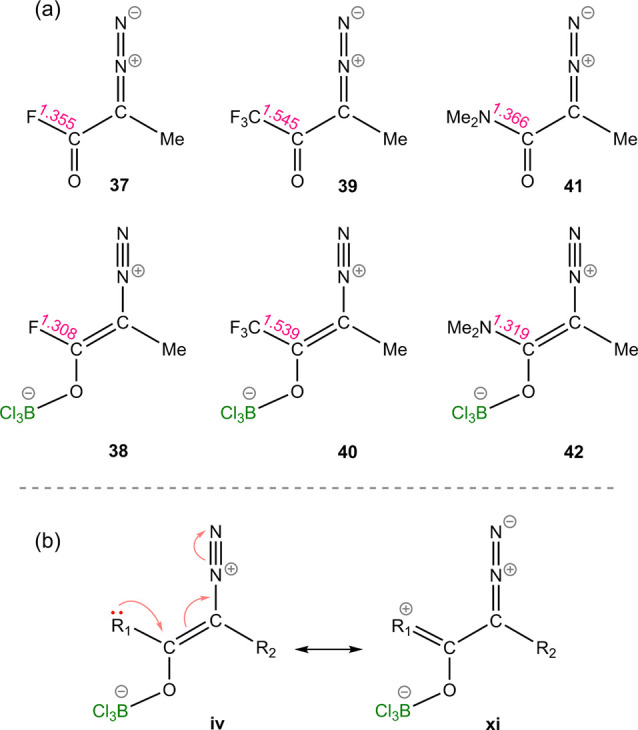
(a) Comparison of the R_1_-carbonyl carbon bond lengths in diazo substrates and B–O adducts. (b) Resonance contributor **xi** as a result of π-donation from R_1_. Interatomic distances are given in Å.

Another noteworthy observation requiring elucidation is an upward shift in activation energy for *path O*, rising from 15.6 to 30.1 kcal/mol, where R_2_ = Me, upon substituting R_1_ from CF_3_ (**39**) to F (**37**), with closely relative energy levels for the B–O adducts (Table 1, Figure 8a). This can be attributed to the π-donating capability of the fluorine atom, which increases the contribution of resonance structure **xi** (Figure 8b), wherein the carbon-diazo bond is reinforced, necessitating higher energy for dissociation. A similar effect is also evident for the NMe_2_ group (**41**), which diminishes the efficacy of the Lewis acid catalyst for *path O*. Comparison of the R_1_-carbonyl carbon bond lengths in the substrates and B–O adducts, as depicted in Figure 8a, reveals a more pronounced shortening of C–F and C–N bonds for F and NMe_2_ substituents in their corresponding adducts (**38** and **42**). This indicates the higher π-donating ability of these substitutions in the adduct compared to the substrate, supporting the participation of resonance structure **xi** (Figure 8b).

Overall, R_1_ π-donating groups, such as alkoxy, phenoxy, and amine, disfavor *path O* by pushing electron density into the π-orbital of C=N. It is noteworthy that diazo substrates with strong π-donating R_1_ groups, such as NMe_2_ and OMe, exhibit low activation energies for N_2_ release even in the absence of a catalyst.^38^ These results suggest that in the presence of borane *path O* is often favored for these substrates.

##### 2.2.2.5 Differential Effects of σ- vs π-Donating Properties of R_1_ on Reaction Pathways

By comparison of samples with different electronic characteristics for R_1_, distinct effects of σ-donating and π-donating properties can be discerned. For example, where R_1_ and R_2_ = CF_3_, Δ*G*^‡^_O_ and Δ*G*^‡^_C_ are calculated to be 47.9 and 32.6 kcal/mol, respectively (Table 1). Changing R_1_ to F, a substituent exhibiting σ-withdrawing and π-donating properties, leads to an increase in Δ*G*^‡^_O_ to 55.6 kcal/mol and a decrease in Δ*G*^‡^_C_ to 29.3 kcal/mol. Replacing R_1_ with Me, a purely σ-donating group, results in a decrease in Δ*G*^‡^_O_ to 36.9 kcal/mol, while Δ*G*^‡^_C_ (30.7 kcal/mol) even increases slightly compared to when fluorine is in the R_1_ position. Introducing OMe, a strong π-donor but σ-withdrawing group, at the R_1_ position yields energy barriers of 42.9 and 19.4 kcal/mol for Δ*G*^‡^_O_ and Δ*G*^‡^_C_, respectively. These observations highlight the contrasting effects of σ- and π-donations on the two pathways. While the trend is somewhat influenced by R_2_ variation, which is primarily driven by changes in the catalyst’s resting state energy, the overall conclusion remains that the σ-donating property of R_1_ is more effective in decreasing Δ*G*^‡^_O_, whereas the π-donating characteristic of R_1_ is particularly efficient at diminishing Δ*G*^‡^_C_.

#### 2.2.3 Correlation Analysis of Descriptors and ΔΔ*G*G

To gain data-driven insights into this transformation, we investigated the relationship between a list of electronic properties of α-diazocarbonyl molecules and the activation energy difference between pathways O and C (ΔΔ*G* = Δ*G*^‡^_C_ – Δ*G*^‡^_O_) for each sample. We initially employed natural population analysis (NPA) charges on the atoms of the α-diazo functionality (i.e., the carbonyl carbon (C^1^), carbonyl oxygen (O), the two diazo nitrogen atoms (N^1^: nitrogen bonded to carbon, N^2^: terminal nitrogen) and the carbon bonded to the diazo group (C^2^)), along with the HOMO energy level (in eV), p_*x*_ orbital occupancy (Occ) of oxygen, and p_*z*_ orbital occupancy of C^1^. It is noteworthy that in the B–O adduct, the boron atom lies in the plane of the diazocarbonyl moiety, receiving electrons from the oxygen’s p_*x*_ orbital. In contrast, the boron center in the B–C adduct interacts with the carbon’s p_*z*_ orbital.

A multiple linear regression analysis was performed to explore the relationship between the proposed descriptors and the computed ΔΔ*G* (in kcal/mol). The resulting equation is as follows:
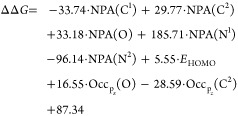
1

The model’s coefficient of determination (*R*^2^) is 0.67, with a mean absolute error (MAE) of 4.62 kcal/mol. These metrics indicate a suboptimal linear correlation between the chosen descriptors and ΔΔ*G*. To address this limitation and potentially uncover more complex (nonlinear) relationships, we opted to employ a machine learning approach.

#### 2.2.4 Machine Learning Approach

**Figure 9:**
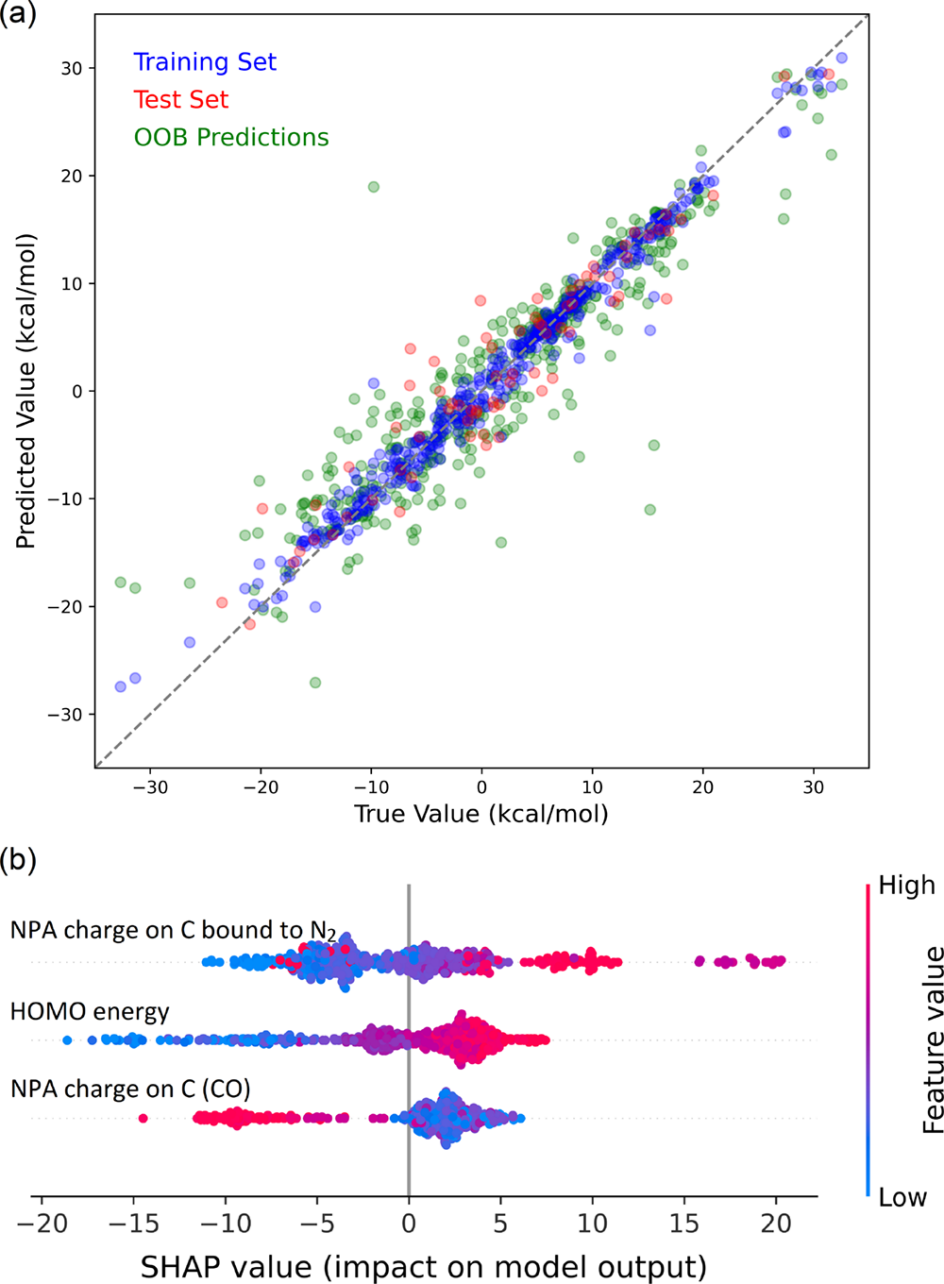
(a) Scatter plots of the DFT values for ΔΔ*G* vs the predicted values from the trained ML model, based on attribute features, for the training, test, and out-of-bag sets. (b) SHAP values for different features across various samples. Colors indicate the feature values, ranging from low (blue) to high (red).

The aforementioned properties were used in the feature space to train a random forest^39^ regression model (see the Supporting Information for the model selection rationale and model details.). Subsequently, to optimize the model’s performance, we excluded features that did not significantly contribute to its predictive ability. This resulted in the following final input features: NPA charges on the carbon bound to N_2_ and the carbonyl carbon along with the HOMO energy level. Figure 9a illustrates the predictability of the trained model. The model attained average *R*^2^ scores of 0.98 on the training set, 0.85 on the validation set, and 0.86 for the out-of-bag samples after 256 model iterations, with corresponding MAEs of 1.03 2.79, and 2.81 kcal/mol, respectively. These results underscore the effectiveness of the input variables provided in our ML model in detecting patterns and learning trends. The SHapley Additive exPlanations (SHAP) analysis^40^ was utilized to decode the importance of features and interpret the ML model. The SHAP analysis aims to dissect a model’s prediction by assessing the contribution of each feature to its predictions. This breakdown highlights the significance of each feature in shaping the model’s prediction. By quantifying the influence of each feature, the SHAP analysis provides a comprehensive and intuitive understanding of the model’s behavior. Figure 9b depicts the impact of the features on the trained model. Accordingly, a more negative partial charge on the carbon atom attached to the diazo group favors *path C*. This is likely due to the strengthening of the boron–carbon bond in the B–C adduct, leading to its enhanced stability. Conversely, a more negative partial charge on the carbonyl carbon enhances the stability of the B–O adduct, resulting in a preference for *path O*.

The machine learning results indicate that a higher HOMO energy level makes *path C* less favorable, which aligns with the DFT-based analysis for the formation of a more stable B–O adduct and ultimately widening of the energy gap between the transition structures for *path C* and the energy reference point (the B–O adduct in such cases). Additionally, according to the ML results, a more negative partial charge on the carbon atom attached to N_2_ facilitates *path C*, suggesting an increased propensity of borane as a Lewis acid to bind to this carbon, leading to a more stable B–C adduct. Conversely, a higher negative partial charge on the carbonyl carbon, based on the ML results, favors *path O*. This is in line with the DFT results, as it leads to a more stable B–O adduct, which is located on *path O*.

Overall, in the presence of BCl_3_, α-diazocarbonyl compounds that possess lower HOMO energy levels and less electron density on the carbonyl carbon (C^1^) are more likely to proceed via *path C*. Conversely, compounds with higher HOMO energy levels and a more negative partial charge on C^1^ tend to react via the *path O*.

## 3 Conclusions

In conclusion, through DFT calculations, we have demonstrated that in carbene transfer reactions catalyzed by borane the prevalent mechanism, which involves the formation of B–O adducts, is not necessarily the preferred pathway. An alternative pathway entails the attachment of borane to carbon bearing the diazo group (*path C*). We evaluated the catalytic pathway favorability by introducing 22 different substituents on both sides of α-diazocarbonyl precursors while utilizing BCl_3_ as a Lewis acid. The activation energies for N_2_ elimination were computed for each sample through three scenarios: via the B–O path, via the B–C path, and without borane.

Our findings demonstrate that σ-donating R_1_ groups (attached to the carbonyl) and σ- or π-donating R_2_ groups (attached to the diazo-bearing carbon) facilitate *path O* (Scheme 1), while π-donating R_1_ substituents favor the *path C* unless R_2_ is a strong π-donor. Indeed, these features influence the stability of the initial adducts (B–O adduct or B–C adduct) and the boron-stabilized carbenes, and the stability of the transition structures is significantly affected by the thermodynamic stability of these species. Additionally, this study depicts the variable catalytic efficiency of borane depending on substrate substitution, suggesting that even in some cases, borane cannot play a catalytic role in the formation of carbene intermediates in either of the two pathways.

Machine learning study revealed that lower electron density on the carbonyl carbon (C^1^) and higher electron density on the diazo-attached carbon (C^2^) facilitate *path C*. This study also demonstrates the variable catalytic performance of boron, depending on substrate substitution, with boron failing to catalyze the formation of a carbene intermediate in a few instances.

The information supplied in this research challenges the conventional understanding of borane-catalyzed carbene transfer reactions and provides valuable insights into the factors governing pathway selection and catalyst performance. The presented findings have implications for the rational design of efficient catalysts for borane-catalyzed carbene transfer reactions.

### 3.1 Computational Details

Gaussian 16^41^ was used to fully optimize all the structures reported in this paper at the M06-2X level of theory.^42^ For all the calculations, solvent effects were considered using the SMD solvation model^43^ with dichloromethane as the solvent. To fully optimize the geometry of structures and subsequently Frequency calculations, the 6-31G(d) basis set^44^ was employed. Transition structures were located using the Berny algorithm. To further refine the energies obtained from the SMD/M06-2*X*/6-31G(d) calculations, we carried out single-point energy calculations using a more accurate def2-TZVP basis set.^45^ Tight convergence criterion and an ultrafine integral grid were also employed to increase the accuracy of the calculations. IRC calculations were used to confirm the connectivity between transition structures and minima.^46,47^

The free energy for each species in solution was calculated by using the following formula:
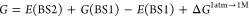
2

We used the random forest regression machine learning model as implemented in the Scikit-learn package^48^ of Python.

## Data Availability

Scripts and dataset available at: https://github.com/KavehFarshadfar/Borane-Diazocarbonyl.

## Supporting Information Available

Computational results; Cartesian coordinates of the calculated species; and machine learning details (PDF)
