# Supplementary material for: Comparison of the Efficiency of B–O and B–C Bond Formation Pathways in Borane-Catalyzed Carbene Transfer Reactions Using α-Diazocarbonyl Precursors: A Combined Density Functional Theory and Machine Learning Study
Source: ACS Catal. 2024 Sep 16;14(19):14486–96. doi: 10.1021/acscatal.4c03368 (PMC11494835; doi:10.1021/acscatal.4c03368)
Supplement: Supplementary file 1 — cs4c03368_si_001.pdf [file cs4c03368_si_001.pdf]

Comparison of the Efficiency of B-O and B-C Bond Formation Pathways in Borane-Catalyzed Carbene Transfer Reactions using α-Diazocarbonyl Precursors: A Combined Density Functional Theory and Machine Learning Study

Kaveh Farshadfar,* and Kari Laasonen*

Department of Chemistry and Material Science, School of Chemical Engineering, Aalto University, 02150 Espoo, Finland

Email: Kaveh.Farshadfar@aalto.fi, Kari.Laasonen@aalto.fi

Content:

Benchmark calculations for key structures (page S2)

Figure S1. DFT-computed pathways for the BCl_3-catalyzed N_2 release of phenyldiazoacetate (**1**) and 2-diazo-1,2-diphenylmethane-1-one (**13**). (page S3)

Figure S2. The efficiency of BCl_3 as a catalyst in the N_2 release from α-diazocarbonyl compounds decorated with R_1 and R_2 via *path O*. (page S4)

Figure S3. The efficiency of BCl_3 as a catalyst in the N_2 release from α-diazocarbonyl compounds decorated with R_1 and R_2 via *path C*. (page S5)

Figure S4. The overall catalytic efficiency of BCl_3 in the N_2 release from α-diazocarbonyl compounds decorated with R_1 and R_2. (page S6)

Table S2. Dataset examples. (page S7)

Figure S5. Plot of $\boldsymbol{\Delta G^{\ddagger}_{C}}$ vs $\boldsymbol{\Delta G_{B\text{-}O\ adduct}}$ for those samples with $\Delta G_{B\text{-}O\ adduct} < 0$. (page S8)

HOMO Energy Level as a Descriptor (page S8)

Machine learning analysis (page S9)

Table S3. The entire dataset of our study. (page S17)

Table S4. Cartesian coordinates and total energies for all of the calculated structures. (page S28)

Benchmark calculations for key structures

We recalculated the key structures of the two studied reactions using the DFT methods M06, M06-D3, and wB97XD (Table S1). It should be noted that the M06 and M06-D3 methods did not converge to a stable geometry for intermediate **7**, suggesting that the transition states **TS$_{1-7}$** and **TS$_{7-5}$** collapse into a single concerted step. This implies that the dissociation of N_2 occurs simultaneously with the addition of borane. Additionally, although the free energies calculated using other DFT methods exhibit similar trends, the potential energy differences better pronounce the energy difference between the two competitive pathways.

Table S1. Relative free energies (in black) and potential energies (in blue) for key structures of the two studied reactions (Figures 2 and 3) calculated using SMD/method/def2-TZVP//SMD/method/6-31g(d).

method	**2**	**TS$_{2-3}$**	**3**	**TS$_{1-7}$**	**7**	**TS$_{7-5}$**	**5**	**TS$_{5-8}$**	**14**	**TS$_{13}$**	**TS$_{14-15}$**
M06-2X	-4.6<br>-19.0	21.3<br>10.3	0.3<br>-1.7	19.4<br>5.5	17.4<br>2.2	18.1<br>5.3	-13.0<br>-16.4	11.9<br>-5.9	-13.0<br>-24.7	27.0<br>13.7	5.9<br>-3.4
M06	6.4<br>-9.8	33.8<br>19.2	6.6<br>1.4			32.2<br>17.3	-4.3<br>-9.4	24.7<br>4.5	-0.2<br>-16.4	41.1<br>25.8	18.8<br>6.5
M06-D3	-0.6<br>-15.3	27.8<br>13.9	3.0<br>-2.6			26.7<br>12.0	-8.6<br>-13.3	15.5<br>-4.5	-5.9<br>-21.9	34.1<br>19.8	13.0<br>1.0
wB97XD	0.7<br>-15.7	28.5<br>14.7	2.5<br>-1.7	27.1<br>11.0	25.9<br>8.3	26.6<br>10.8	-11.9<br>-17.4	15.1<br>-4.3	-7.2<br>-22.5	31.4<br>15.0	12.8<br>0.4

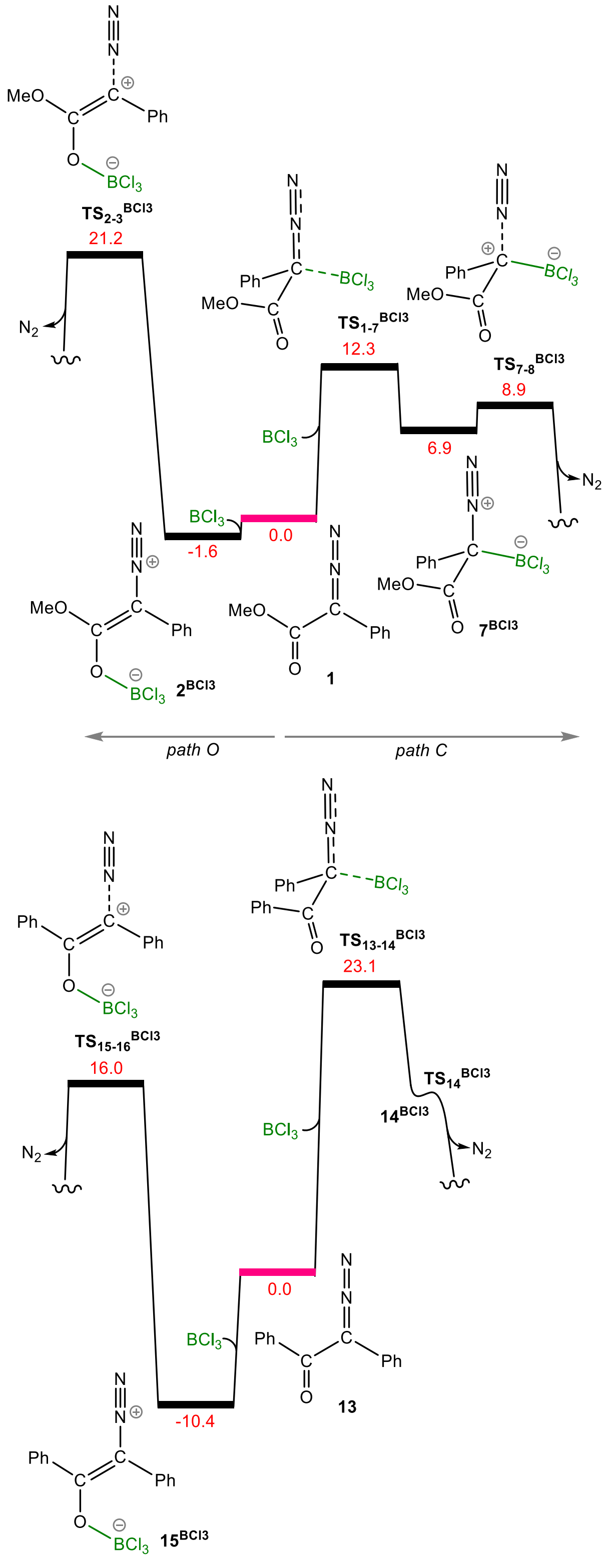

Figure S1. DFT-computed pathways for the BCl_3-catalyzed N_2 release of phenyldiazoacetate (**1**) and 2-diazo-1,2-diphenylmethane-1-one (**13**)

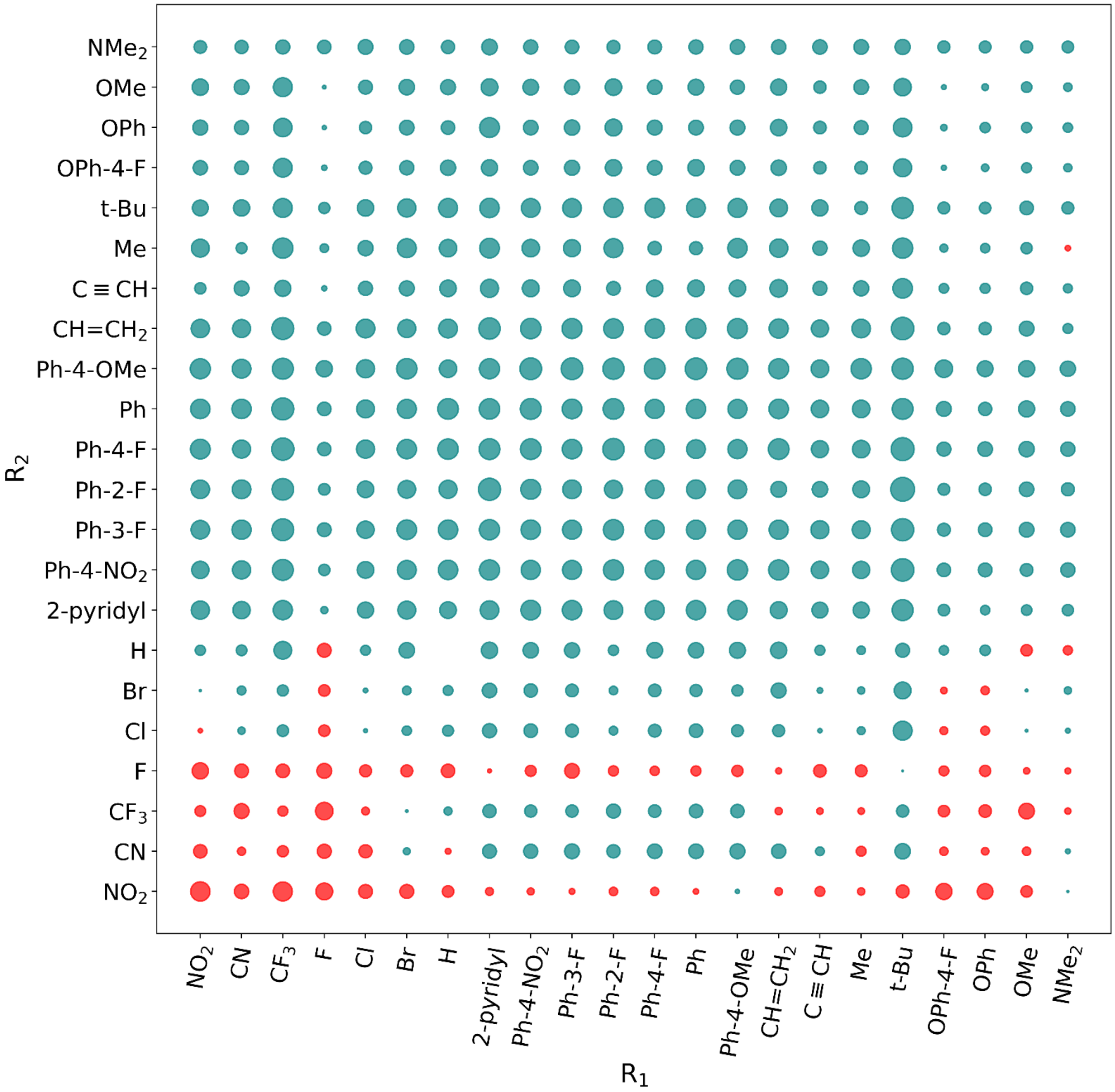

Figure S2. The efficiency of BCl_3 as a catalyst in the N_2 release from α-diazocarbonyl compounds decorated with R_1 and R_2 via *path O*. The size of the teal circles corresponds to the efficiency in reducing the activation energy, while the red color indicates that BCl3 not only fails to act as a catalyst but also elevates the activation energy through *path O*.

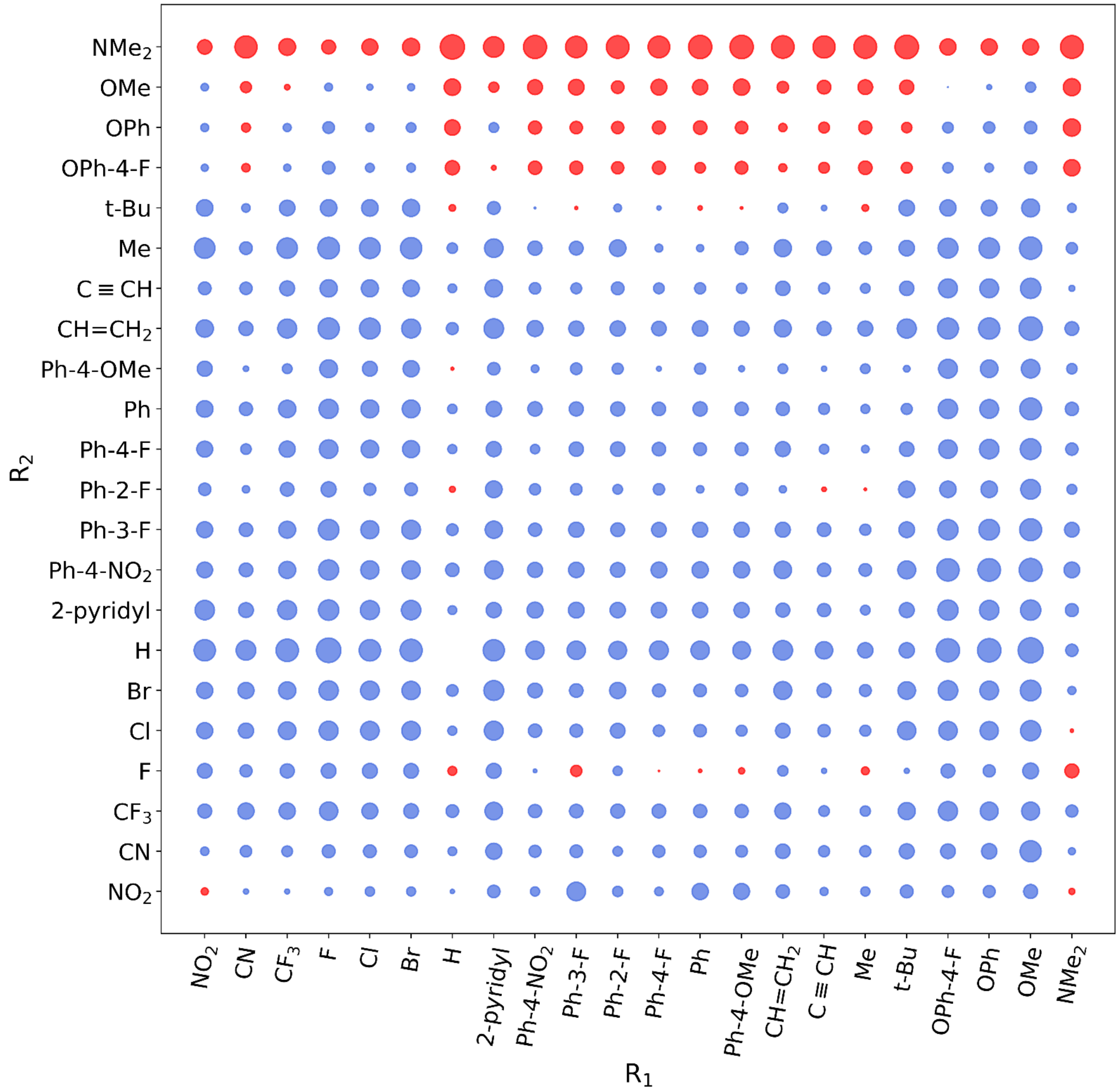

Figure S3. The efficiency of BCl_3 as a catalyst in the N_2 release from α-diazocarbonyl compounds decorated with R_1 and R_2 via *path C*. The size of the blue circles corresponds to the efficiency in reducing the activation energy, while the red color indicates that BCl_3 not only fails to act as a catalyst but also elevates the activation energy through *path C*.

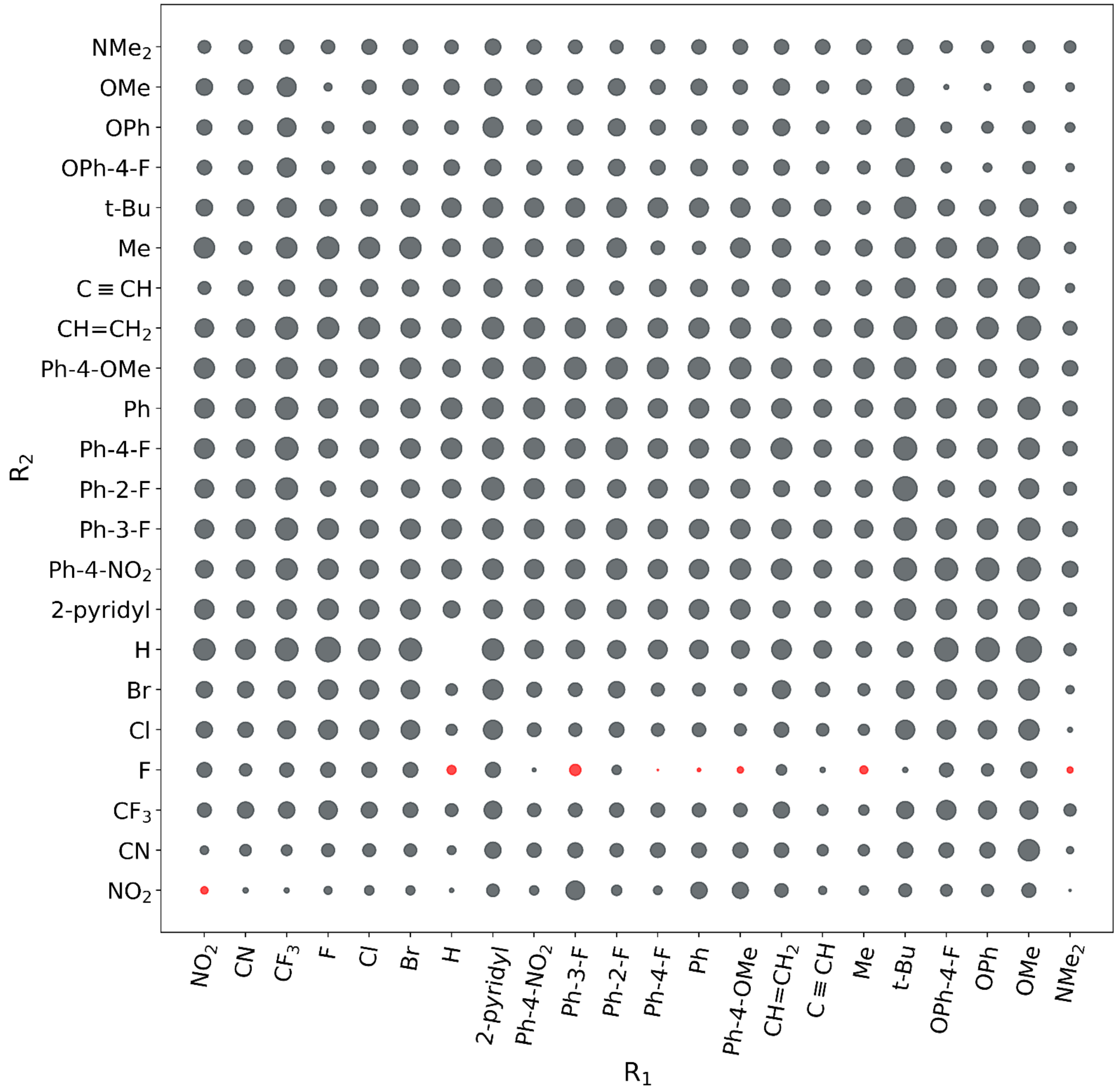

Figure S4. The overall catalytic efficiency of BCl_3 in the N_2 release from α-diazocarbonyl compounds decorated with R_1 and R_2. The size of the gray circles corresponds to the efficiency in reducing the activation energy, while the red color indicates that BCl_3 not only fails to act as a catalyst but also elevates the activation energy.

Table S2. Dataset examples: i.e., free energy barrier of N_2 release (without BCl_3, pathways C and O), $\Delta G^{\ddagger}_{C}$ - $\Delta G^{\ddagger}_{O}$, relative stability of the B-O adduct, NPA charge on backbone carbons, and HOMO energy level for each diazo substrate.

R_2 \ R_1	Me		^{t}Bu		F		CF_3		OMe		NMe_2		$CH=CH_2$		C_6H_5		C_6H_4-4-NO_2	
Me	30.7	-17.9	32.3	-10.8	33.3	-2.8	32.6	-2.2	32.9	-4.2	32.2	-17.6	33.8	-11.2	28.0	-18.8	33.5	-11.9
	21.0	0.54	15.3	0.56	30.1	0.89	15.6	0.43	27.6	0.81	33.5	0.68	19.9	0.50	20.9	0.53	20.8	0.53
	25.8	-0.10	22.3	-0.10	13.8	-0.16	15.4	-0.11	12.3	-0.13	27.0	-0.10	21.6	-0.09	25.8	-0.09	25.1	-0.09
	4.8	-7.61	7.0	-7.57	-16.2	-8.04	-0.2	-8.08	-15.3	-7.62	-6.5	-7.38	1.8	-7.64	4.9	-7.63	4.3	-7.80
^{t}Bu	30.5	-17.7	33.9	-4.6	31.7	-3.6	32.2	-2.0	31.4	-2.0	32.8	-15.8	31.8	-11.8	32.2	-13.8	31.8	-12.1
	23.6	0.55	15.4	0.56	26.5	0.90	17.4	0.44	23.7	0.44	26.8	0.69	18.9	0.51	17.6	0.54	17.1	0.53
	32.5	-0.09	23.7	-0.08	20.4	-0.15	22.2	-0.11	18.1	-0.11	29.5	-0.09	27.9	-0.07	33.0	-0.08	31.7	-0.08
	8.8	-7.62	8.3	-7.58	-6.2	-8.02	4.8	-8.07	-5.7	-7.61	2.7	-7.36	8.9	-7.64	15.4	-7.64	14.6	-7.79
F	19.5	-12.8	20.8	-7.9	20.0	1.9	22.1	1.9	18.6	-0.1	15.9	-13.7	20.8	-6.8	19.6	-8.4	19.6	-6.5
	25.3	0.49	20.6	0.51	29.2	0.84	29.7	0.38	20.4	0.76	17.4	0.63	22.4	0.45	23.8	0.48	24.6	0.48
	22.1	0.34	19.7	0.35	11.0	0.28	13.8	0.34	8.3	0.32	23.9	0.34	16.3	0.35	20.1	0.35	19.0	0.35
	-3.2	-8.02	-1.0	-7.97	-18.2	-8.49	-15.9	-8.52	-12.0	-8.04	6.5	-7.76	-6.0	-8.04	-3.7	-8.03	-5.7	-8.20
Cl	26.4	-10.7	28.5	0.7	28.0	1.6	29.0	3.1	26.4	1.3	24.7	-13.0	27.2	-7.2	29.0	-8.0	29.6	-6.4
	23.7	0.53	13.6	0.55	33.4	0.89	23.5	0.42	26.1	0.80	23.7	0.67	21.2	0.49	21.4	0.52	21.9	0.52
	21.3	-0.14	14.8	-0.13	13.2	-0.21	16.1	-0.16	9.6	-0.16	25.5	-0.14	18.3	-0.13	22.5	-0.13	22.2	-0.14
	-2.4	-7.95	1.2	-7.91	-20.1	-8.36	-7.4	-8.41	-16.5	-7.95	1.8	-7.71	-2.9	-7.96	1.1	-7.96	0.4	-8.12
CF_3	35.1	-10.1	37.6	2.3	43.1	4.1	43.6	6.9	32.8	-0.6	34.7	-10.4	36.7	-4.5	37.5	-5.6	38.3	-3.9
	36.9	0.56	31.4	0.57	55.6	0.91	47.9	0.45	42.9	0.82	36.4	0.69	39.0	0.52	29.9	0.55	32.1	0.54
	30.7	-0.23	25.4	-0.23	29.3	-0.28	32.6	-0.24	19.4	-0.26	28.6	-0.23	26.1	-0.22	30.1	-0.23	31.0	-0.22
	-6.2	-8.67	-6.0	-8.63	-26.4	-9.18	-15.2	-9.19	-23.5	-8.72	-7.7	-8.32	-12.9	-8.69	0.2	-8.64	-1.1	-8.84
NO_2	32.0	-4.0	32.6	5.5	35.9	7.3	34.8	9.3	33.8	6.5	29.0	-7.8	32.6	-1.6	33.4	-2.5	33.7	-0.4
	34.3	0.55	39.6	0.56	47.6	0.90	49.7	0.44	39.2	0.81	28.8	0.68	35.1	0.51	34.6	0.54	35.8	0.54
	28.5	0.01	25.8	0.01	33.4	-0.05	33.7	-0.01	25.7	-0.01	30.5	0.01	25.2	0.02	22.4	0.01	30.2	0.01
	-5.8	-9.09	-13.9	-9.05	-14.3	-9.61	-16.0	-9.60	-13.5	-9.17	1.8	-8.57	-9.8	-9.10	-12.2	-8.77	-5.7	-9.24
OMe	15.7	-15.5	17.1	-13.7	14.8	-2.1	15.4	-3.7	15.0	-3.2	13.8	-16.8	15.4	-11.8	15.2	-12.8	14.6	-11.0
	6.7	0.51	4.6	0.52	14.3	0.86	0.7	0.39	10.3	0.78	10.8	0.64	4.7	0.47	4.8	0.50	4.3	0.49
	24.7	0.23	25.5	0.23	12.2	0.17	16.7	0.22	10.8	0.20	26.0	0.23	21.0	0.24	24.9	0.23	23.8	0.23
	18.0	-7.65	20.9	-7.64	-2.0	-8.11	16.0	-8.15	0.4	-7.68	15.2	-7.37	16.3	-7.70	20.1	-7.71	19.4	-7.87
NMe_2	9.9	-18.3	9.3	-18.1	8.3	-3.2	7.5		8.9	-9.2	11.0	-22.2	7.9	-17.0	8.0	-17.4	8.3	-17.0
	0.9	0.53	-0.2	0.55	0.8	0.88	-0.6	0.42	2.8	0.80	5.3	0.67	-0.7	0.49	-0.3	0.52	-0.1	0.52
	31.3	0.05	32.3	0.09	16.4	0.01	19.1	0.06	19.3	0.05	32.5	0.07	29.1	0.09	30.4	0.08	30.3	0.08
	30.4	-7.64	32.5	-7.29	15.6	-8.14	19.8	-8.06	16.6	-7.54	27.3	-7.23	29.8	-7.35	30.7	-7.42	30.4	-7.57
$CH=CH_2$	33.4	-13.5	35.3	-5.9	34.5	-0.7	34.8	1.4	34.6	-1.9	34.7	-15.3	34.1	-10.6	34.7	-10.6	35.0	-9.0
	19.1	0.56	14.4	0.57	27.1	0.91	15.0	0.44	25.4	0.82	30.6	0.69	18.9	0.52	18.2	0.55	17.7	0.54
	24.1	-0.13	20.2	-0.13	15.9	-0.18	19.9	-0.14	12.1	-0.15	26.9	-0.13	22.4	-0.12	24.7	-0.12	24.2	-0.12
	5.0	-7.37	5.8	-7.34	-11.1	-7.70	4.9	-7.74	-13.3	-7.36	-3.8	-7.17	3.5	-7.39	6.6	-7.39	6.7	-7.52
C_6H_5	29.5	-16.2	30.3	-12.7	30.1	-1.8	30.3	0.1	32.0	-1.6	33.5	-14.4	29.9	-11.4	31.5	-10.4	31.2	-9.3
	16.7	0.56	12.4	0.57	22.5	0.91	10.2	0.44	21.2	0.82	24.8	0.69	14.1	0.52	16.0	0.55	13.2	0.54
	26.1	-0.10	25.3	-0.09	14.9	-0.15	17.8	-0.10	12.3	-0.12	26.2	-0.09	22.6	-0.08	23.1	-0.09	22.7	-0.09
	9.4	-7.25	12.9	-7.22	-7.6	-7.50	7.5	-7.56	-8.9	-7.20	1.4	-7.02	8.5	-7.26	7.0	-7.26	9.5	-7.38
C_6H_4-4-F	28.6	-15.9	31.0	-7.7	29.0	-2.1	28.1	-1.3	31.1	-2.0	32.7	-14.4	30.9	-9.3	29.6	-11.4	30.4	-9.4
	15.8	0.56	9.1	0.57	21.6	0.91	7.6	0.44	21.2	0.82	24.2	0.69	13.2	0.52	15.1	0.55	12.6	0.54
	26.1	-0.10	22.1	-0.09	14.6	-0.15	17.4	-0.10	13.1	-0.12	26.4	-0.09	21.5	-0.08	22.8	-0.09	26.7	-0.09
	10.3	-7.22	13.0	-7.20	-7.0	-7.46	9.8	-7.53	-8.1	-7.18	2.1	-7.0	8.3	-7.23	7.7	-7.22	14.1	-7.35
C_6H_4-4-OMe	29.7	-14.1	27.9	-13.6	28.3	-1.0	25.5	-5.7	27.9	-4.2	31.1	-15.3	27.5	-12.3	29.4	-11.1	28.9	-10.4
	12.7	0.56	9.5	0.57	17.3	0.91	7.2	0.44	17.0	0.82	21.4	0.69	10.9	0.51	10.1	0.54	9.4	0.54
	25.8	-0.09	26.1	-0.09	15.2	-0.15	21.5	-0.10	13.6	-0.11	26.6	-0.09	23.6	-0.08	24.2	-0.09	26.5	-0.09
	13.1	-6.84	16.6	-6.83	-2.1	-7.04	14.3	-7.10	-3.4	-6.81	5.1	-6.67	12.7	-6.86	14.0	-6.89	17.1	-6.98
C_6H_4-4-NO_2	31.4	-13.3	34.8	-4.7	33.6	0.1	33.5	2.6	34.1	-0.7	36.5	-12.6	34.0	-7.6	34.5	-7.6	34.5	-6.5
	19.1	0.56	13.8	0.58	28.2	0.92	15.4	0.45	27.4	0.83	28.2	0.69	17.1	0.52	19.2	0.55	19.3	0.55
	24.8	-0.10	21.5	-0.10	17.0	-0.16	21.5	-0.11	12.3	-0.12	26.3	-0.10	21.4	-0.09	23.6	-0.10	25.1	-0.10
	5.7	-7.62	7.7	-7.60	-11.2	-7.90	6.1	-7.96	-15.0	-7.59	-1.9	-7.38	4.3	-7.62	4.4	-7.61	5.8	-7.73
2-pyridyl	30.7	-13.8	29.6	-9.4	32.4	-0.6	30.4	-0.1	30.5	-3.8	33.6	-14.5	29.5	-11.1	32.4	-9.6	32.1	-7.9
	19.9	0.56	11.4	0.57	30.2	0.91	15.1	0.45	25.8	0.82	28.3	0.7	17.7	0.52	17.0	0.55	16.0	0.55
	26.8	-0.12	20.3	-0.11	15.2	-0.17	15.5	-0.12	13.5	-0.14	26.6	-0.11	21.4	-0.10	22.8	-0.12	21.5	-0.12
	7.0	-7.49	8.9	-7.42	-15.0	-7.76	0.4	-7.77	-12.3	-7.43	-1.7	-7.27	3.7	-7.46	5.8	-7.50	5.5	-7.63

black: $\Delta G^{\ddagger}$ in absence of BCl_3 blue: $\Delta G^{\ddagger}_{O}$ green: $\Delta G^{\ddagger}_{C}$ red: $\Delta G^{\ddagger}_{C}$ - $\Delta G^{\ddagger}_{O}$ pink: $\Delta G_{\text{B-O adduct}}$

amber: NPA charge on C (CO) brown: NPA charge on C bound to N_2 gray: HOMO energy

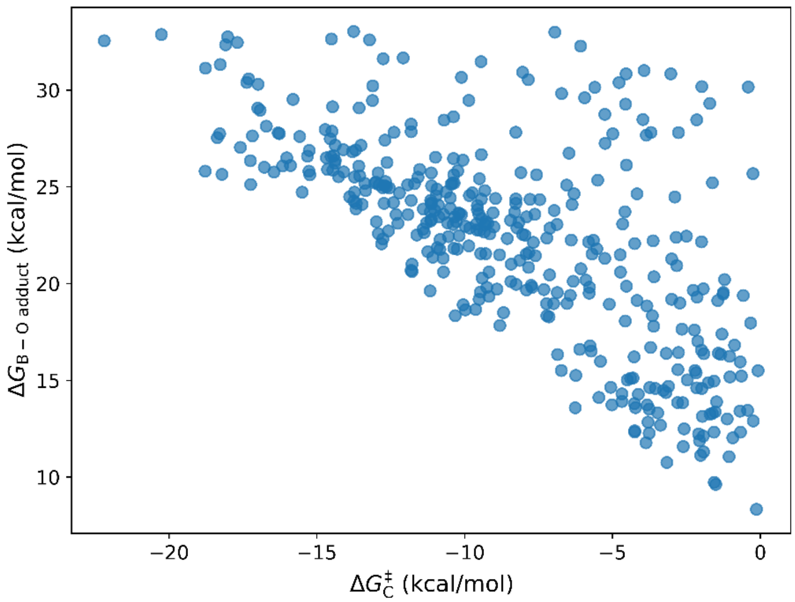

Figure S5. Plot of $\mathbf{\Delta G^{\ddagger}_{C}}$ vs $\mathbf{\Delta G_{B\text{-}O\ adduct}}$ for those samples with $\Delta G_{B\text{-}O\ adduct} < 0$.

HOMO Energy Level as a Descriptor

The primary reason for selecting the HOMO energy level as a descriptor is that it serves as a quantitative indicator of the electron-withdrawing and electron-donating properties of the substituents on both sides of the diazo functionality, which exert differential influence on the two reaction pathways. Additionally, we have demonstrated in Figure 5 that there is a significant correlation between the HOMO energy level and the stability of the B-O adduct, a crucial intermediate that significantly influences the catalytic pathway selection. As depicted in Figure S5, the vacant boron p orbital interacts with the in-plane lone pair of electrons on oxygen (p_x) in the B-O adduct, which is predominantly localized in lower-lying HOMOs. For most of the investigated samples, these oxygen lone pairs are located in HOMO-1, but not for all. In Figure S5, it can be observed that for some diazo substrates, this orbital is located in HOMO-2 or even HOMO-3. The higher the energy of this orbital, the more readily the lone pair can be donated from the diazo molecule to the boron atom. There exists a good linear correlation between the HOMO energy level and the MO energy level involved in the formation of the B-O adduct, regardless of which underlying layer the orbital is in, as evidenced by an R_2 value of 0.86 for the examples shown in Figure S5. This indicates why the HOMO energy level can serve as a descriptor for ΔΔG.

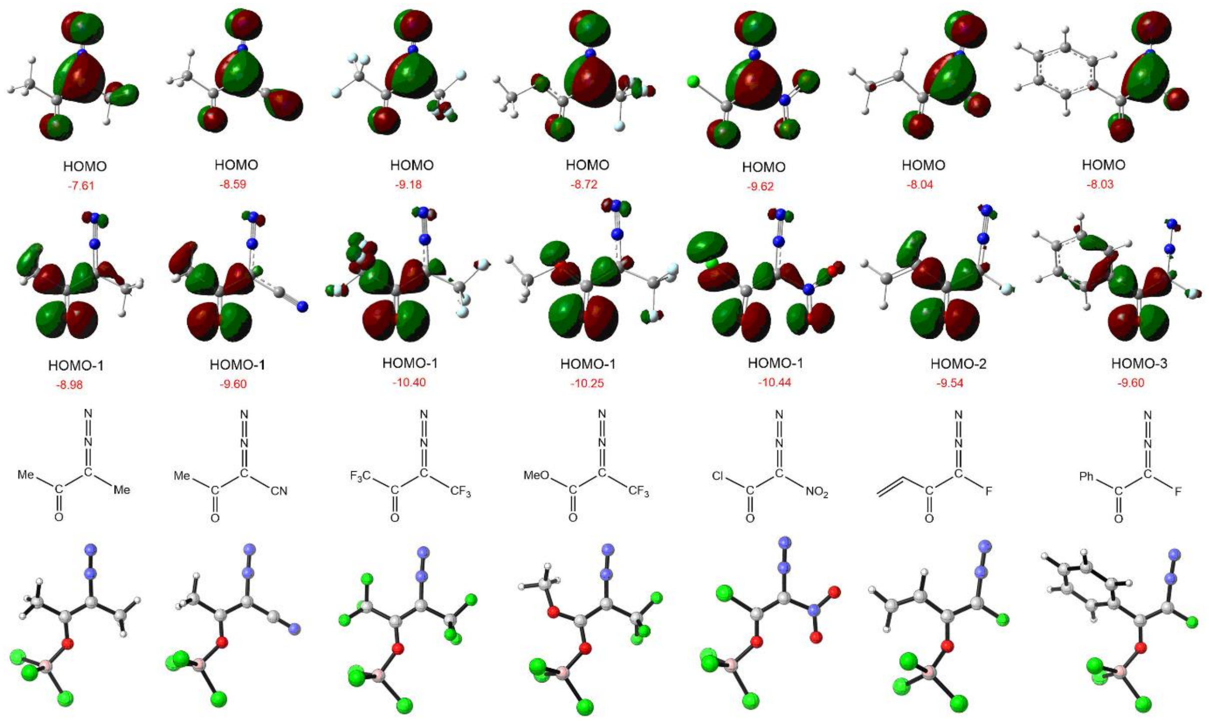

Figure S6. Spatial plots of the HOMO and the HOMO-n involved in B-O adduct formation for some examples from the studied dataset, along with 3D images of their corresponding B-O adducts.

Machine learning analysis

In this study, we evaluated several machine learning models to predict ΔΔG values, including Linear Regression, Decision Tree, Support Vector Machine, K-Nearest Neighbors, Gradient Boosting, Neural Network, and Random Forest. The performance metrics for these models in the initial training are summarized below:

Linear Regression: MAE: 4.730, R^2: 0.734
Decision Tree: MAE: 2.867, R^2: 0.880
Support Vector Machine: MAE: 5.565, R^2: 0.660
K-Nearest Neighbors: MAE: 3.631, R^2: 0.834
Gradient Boosting: MAE: 3.002, R^2: 0.907
Neural Network: MAE: 4.314, R^2: 0.820
Random Forest: MAE: 2.860, R^2: 0.904

Among these models, random forest and gradient boosting regression demonstrated strong performance. While gradient boosting achieved a slightly higher coefficient of determination (R^2), random forest was ultimately selected due to its lower mean absolute error (MAE) and its robustness in handling complex, nonlinear relationships and its ability to mitigate overfitting through ensemble learning.

Architecture of the Random Forest Regression Model:

- **Number of Trees:** 200
- **Minimum Samples to Split an Internal Node:** 2
- **Maximum Features Considered for Splitting:** Auto (square root of the number of features)
- **Minimum Samples at a Leaf Node:** 1
- **Out-of-Bag (OOB) Estimation:** Enabled
- **Random State:** Used for reproducibility
- **Cross-Validation:** 10-fold cross-validation for hyperparameter tuning

Summary of Training, Validation, and Out-of-Bag Set Selection

To evaluate the performance of our Random Forest model, we implemented a robust scheme for selecting training, validation, and out-of-bag (OOB) sets. The process is as follows:

1. Data Shuffling and Splitting:
    - The entire dataset is first shuffled to ensure randomness.
    - The shuffled data is then split into two sets: 85% for training and 15% for testing. This split is achieved using the train_test_split function with a randomly chosen seed for reproducibility.
2. Model Training with Cross-Validation and OOB Estimation:
    - We initialize a RandomForestRegressor with oob_score=True to enable OOB error estimation.
    - Using GridSearchCV, we perform hyperparameter tuning with 10-fold cross-validation. This ensures that the model's performance is evaluated on different subsets of the training data, thus providing a robust assessment.
3. Multiple Iterations for Robustness:
    - To ensure the reliability of our results, the entire process (shuffling, splitting, training, and evaluating) is repeated 256 times with different random seeds.
    - This approach mitigates the impact of any single random train-test split and provides statistically significant performance metrics.
4. Performance Metrics Collection:
    - For each iteration, we predict the target values for the training, validation (test), and OOB sets.
    - We calculate the Mean Absolute Error (MAE) and R^2 scores for each set and store these metrics.
5. Summary of Metrics:
    - After all iterations, we calculate the average and standard deviation of the MAE and R^2 scores for the training, validation, and OOB sets.
    - These metrics provide a comprehensive view of the model's performance and its consistency across different data splits.

Our methodology ensures a thorough and unbiased evaluation of the Random Forest model, leveraging cross-validation and out-of-bag estimation to maximize the use of available data while maintaining rigorous performance assessment standards. This approach allows for robust model validation and reliable performance metrics reporting.

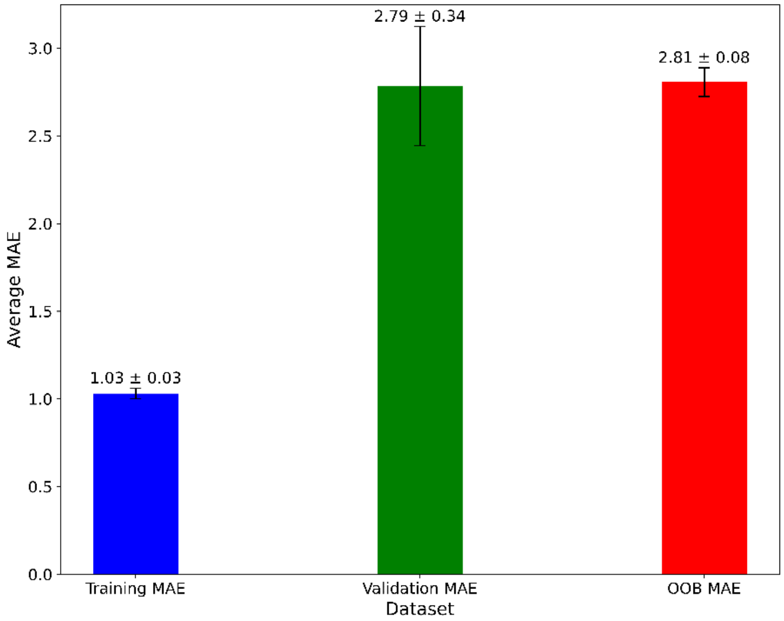

Figure S6. Mean Absolute Error (MAE) for training, validation, and out-of-bag sets after 256 model iterations. Error bars represent standard deviation.

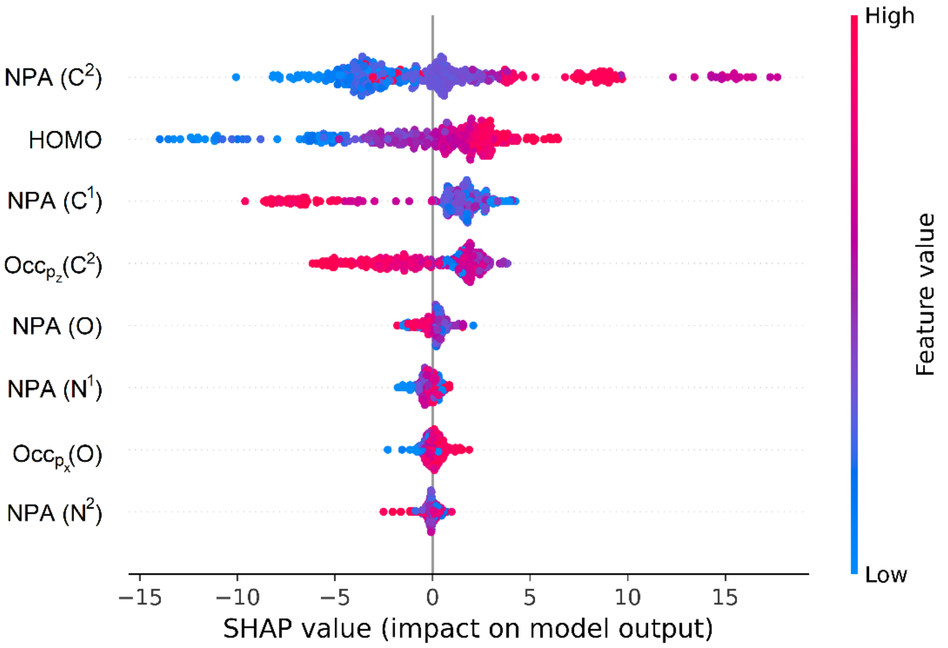

Figure S7. SHAP values for all DFT-based features obtained from a Random Forest regression model.

Explaining a linear logistic regression model

SHAP analysis reveals key electronic features influencing pathway preference (Figure 7b, Figure S5). Figure S5a depicts the dependence plot for HOMO energy. A positive correlation is observed between the HOMO energy level of diazo substrates and their SHAP value. This suggests that substrates with lower (more negative) HOMO energies exhibit more negative ΔΔG ($\Delta G^{\ddagger}_{C}$ - $\Delta G^{\ddagger}_{O}$) values, favoring path C. Each dot in the plot in this plot is colored based on the Natural Population Analysis (NPA) charge on the carbon bound to the diazo functionality (C^2). Substrates with HOMO energies more stable than -8.3 eV, those bearing a more positive charge on C^2 tend to have more negative ΔΔG values. The SHAP dependence plot for NPA charge on C^2 (Figure S5b) displays an inverted V-shape. This indicates that for a significant portion of the dataset, a more negative NPA charge on C^2 correlates with a more negative shift in ΔΔG. However, no clear correlation is observed with HOMO energy levels. Finally, the SHAP dependence plot for NPA charge on C^1 (Figure S5c) reveals no clear dependence on the NPA charge of C^2. However, it suggests that a more positive charge on C^1 leads to a more negative shift in ΔΔG.

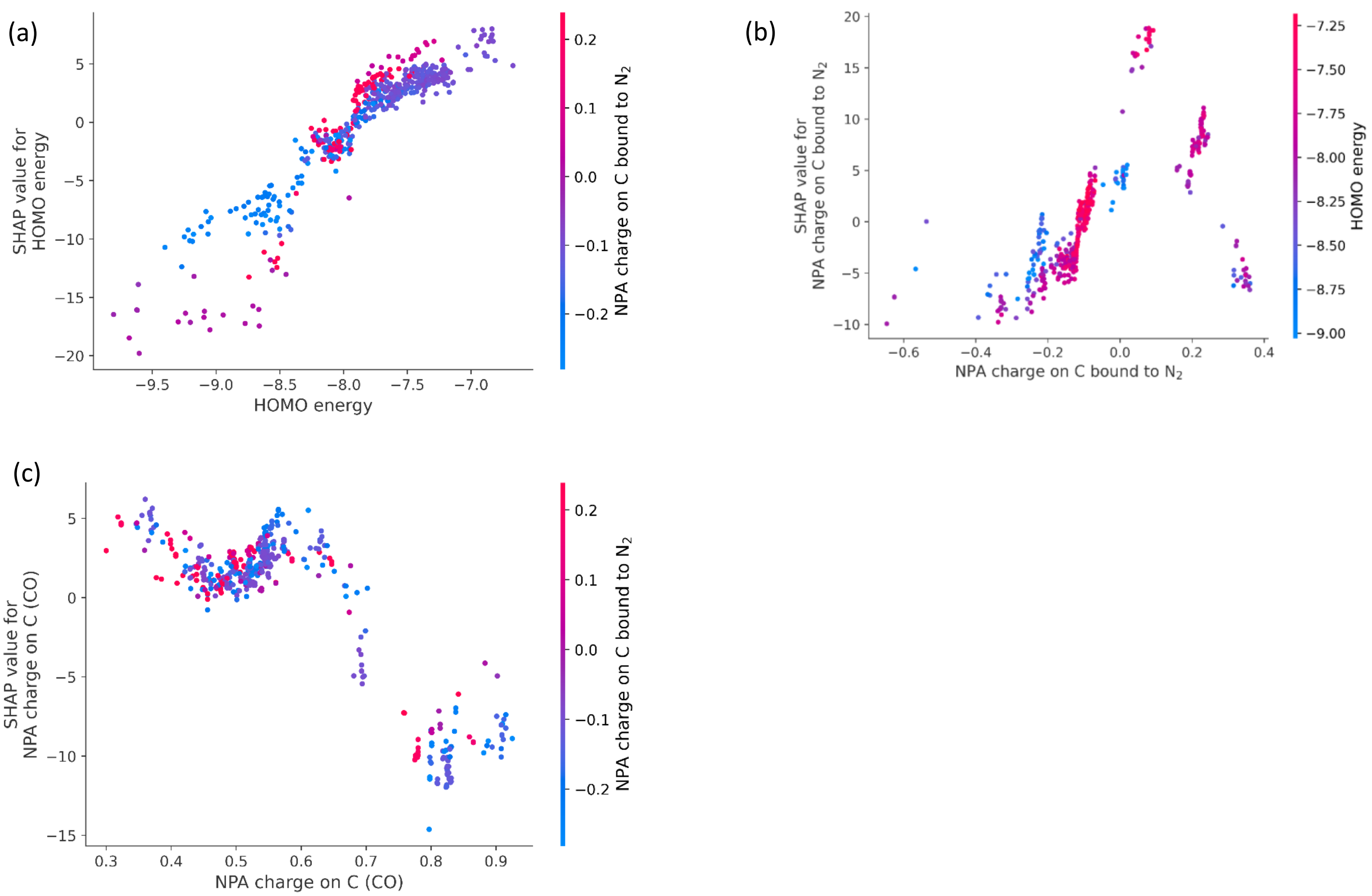

Figure S8. SHAP dependance plot of (a) HOMO energy (b) NPA charge on C bound to N_2 (C^2), and (c) NPA charge on carbonyl carbon (C^1).

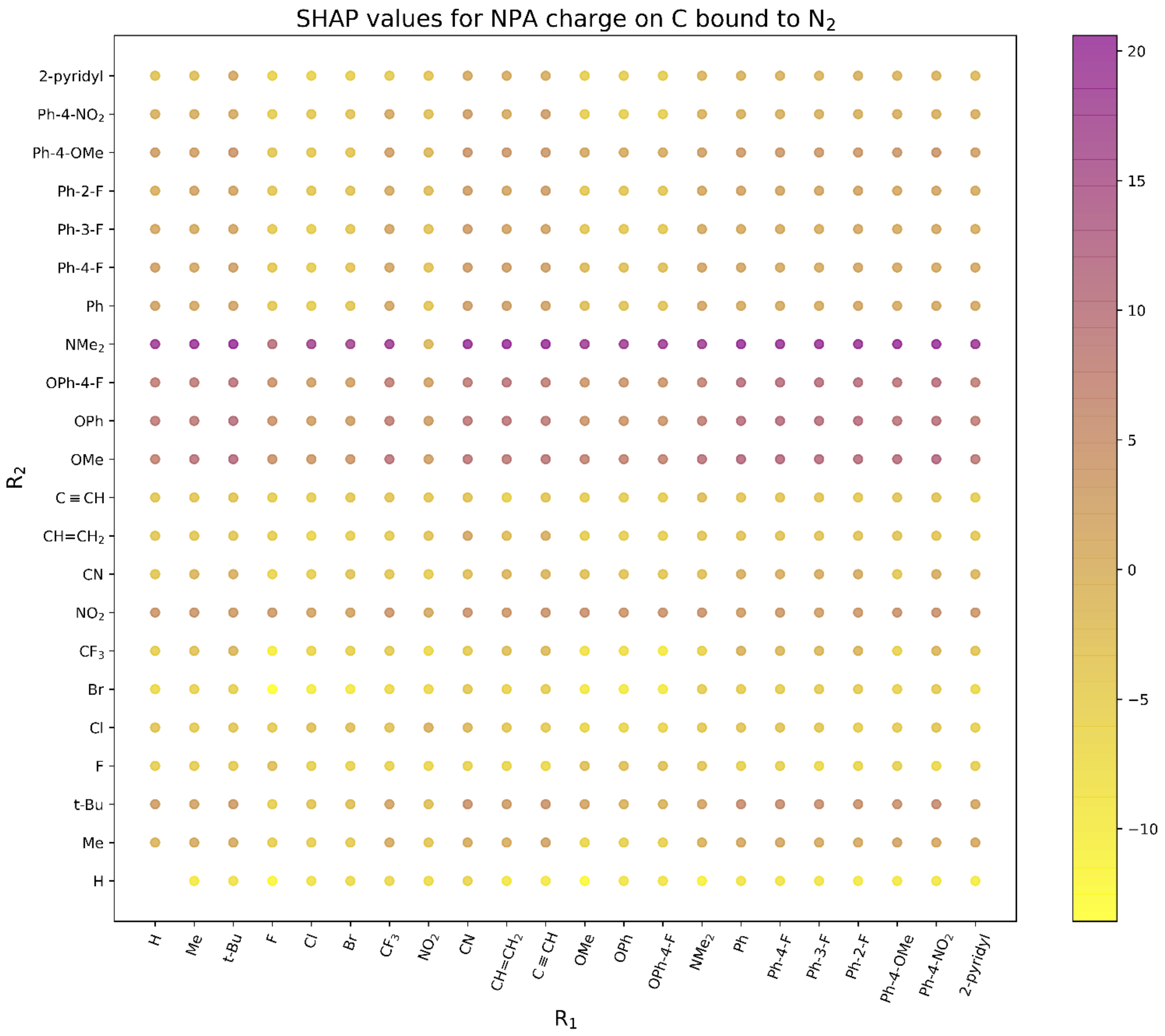
SHAP values for NPA charge on C bound to N_2
2-pyridyl
Ph-4-NO_2
Ph-4-OMe
Ph-2-F
Ph-3-F
Ph-4-F
Ph
NMe_2
OPh-4-F
OPh
OMe
C≡CH
CH=CH_2
CN
NO_2
CF_3
Br
Cl
F
t-Bu
Me
H
R_2
H
Me
t-Bu
F
Cl
Br
CF_3
NO_2
CN
CH=CH_2
C≡CH
OMe
OPh
OPh-4-F
NMe_2
Ph
Ph-4-F
Ph-3-F
Ph-2-F
Ph-4-OMe
Ph-4-NO_2
2-pyridyl
R_1
20
15
10
5
0
−5
−10

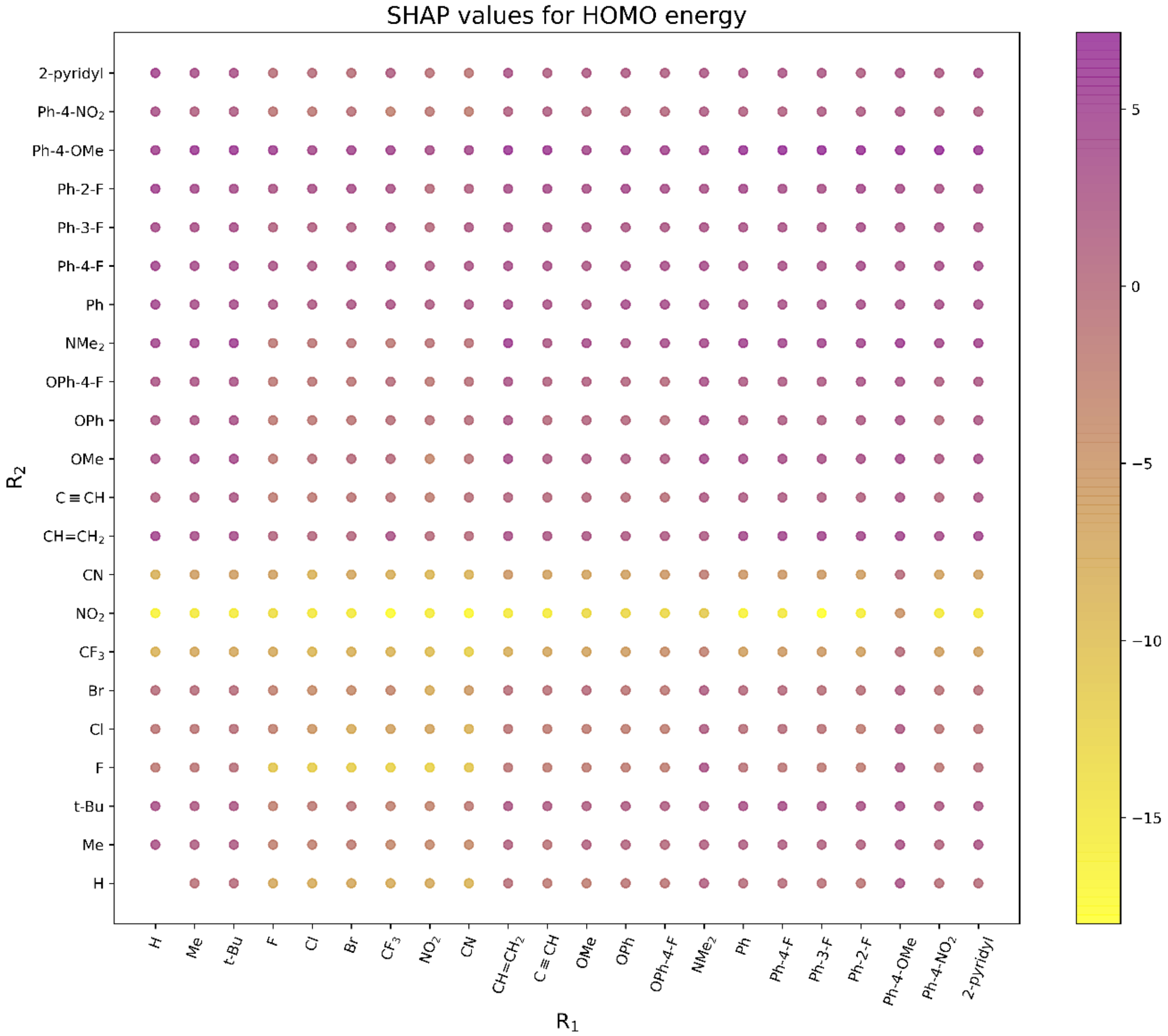
SHAP values for HOMO energy
2-pyridyl
Ph-4-NO2
Ph-4-OMe
Ph-2-F
Ph-3-F
Ph-4-F
Ph
NMe2
OPh-4-F
OPh
OMe
C≡CH
CH=CH2
CN
NO2
CF3
Br
Cl
F
t-Bu
Me
H
R2
H
Me
t-Bu
F
Cl
Br
CF3
NO2
CN
CH=CH2
C≡CH
OMe
OPh
OPh-4-F
NMe2
Ph
Ph-4-F
Ph-3-F
Ph-2-F
Ph-4-OMe
Ph-4-NO2
2-pyridyl
R1
5
0
−5
−10
−15

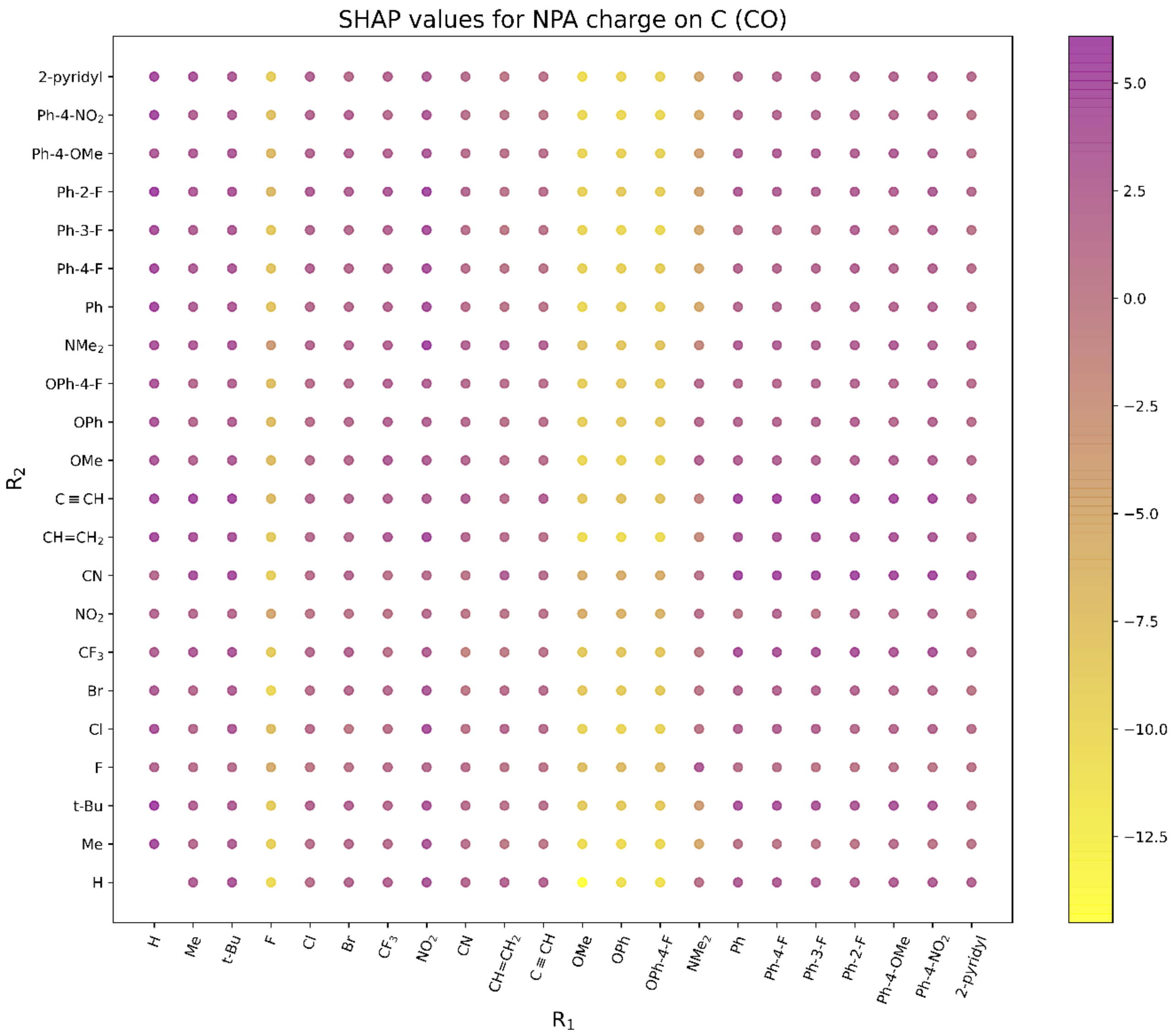

Figure 9: SHAP value plots of R_1 and R_2 for the most important features. These plots illustrate the contribution of each feature to the model's predictions.

Table S3. The entire dataset of our study: In α-diazocarbonyl compound naming, the first number represents R_1, and the second number represents R_2. The numbers 1 to 22 correspond to the following substituents: 1: H (Hydrogen), 2: Me (Methyl), 3: ^{t}Bu (tert-Butyl), 4: F (Fluorine), 5: Cl (Chlorine), 6: Br (Bromine), 7: CF_3 (Trifluoromethyl), 8: NO_2 (Nitro), 9: CN (Cyano), 10: CH=CH_2 (Vinyl), 11: C≡CH (Ethynyl), 12: OMe (Methoxy), 13: OPh (Phenoxy), 14: OPh-4-F (4-Fluorophenoxy), 15: NMe_2 (Dimethylamino), 16: Ph (Phenyl), 17: Ph-4-F (4-Fluorophenyl), 18: Ph-3-F (3-Fluorophenyl), 19: Ph-2-F (2-Fluorophenyl), 20: Ph-4-OMe (4-Methoxyphenyl), 21: Ph-4-NO_2 (4-Nitrophenyl), 22: 2-pyridyl (2-Pyridyl). p_z (C^2), and p_x (O) represent electron populations on the $2p_z$ orbital of diazo-attached carbon (C^2), and the $2p_x$ orbital of the carbonyl oxygen respectively. NPA (C^2), NPA (N^1), NPA (N^2), NPA (C^1), and NPA (O) demonstrate NPA charges on C^2, N^1 (nitrogen attached to carbon), N^2 (terminal nitrogen), C^1(carbonyl carbon), and O respectively. HOMO represents the HOMO energy level of diazo substrate in eV. $\Delta G^\ddagger$, $\Delta G_{B\text{-}O}$, $\Delta G^\ddagger_O$, and $\Delta G^\ddagger_C$ represent the free energy barrier for N_2 release without borane, relative free energy of B-O adduct, and free energy barrier for N_2 release in path O and path C correspondingly.

name	p_z (C^2)	p_x (O)	NPA (C^2)	NPA (N^1)	NPA (N^2)	NPA (C^1)	NPA (O)	HOMO	$\Delta G^\ddagger$	$\Delta G_{B\text{-}O}$	$\Delta G^\ddagger_O$	$\Delta G^\ddagger_C$
**16_17**	1.25984	1.86131	-0.089	0.086	0.08	0.547	-0.599	-7.221	29.64	-11.41	15.11	22.81
**8_19**	1.28716	1.8009	-0.14	0.119	0.16	0.631	-0.533	-7.976	30.58	4.55	16.46	24.22
**18_19**	1.18824	1.85751	-0.105	0.09	0.089	0.547	-0.595	-7.511	30.79	-10.36	16.39	25.19
**19_4**	1.16975	1.86966	0.348	0.005	0.079	0.477	-0.569	-8.086	22.05	-8.69	26.36	18.5
**19_8**	1.23926	1.85916	0.009	0.091	0.188	0.537	-0.529	-8.665	33.71	-1.71	36.73	29.31
**5_10**	1.30007	1.78232	-0.154	0.102	0.135	0.534	-0.542	-7.757	35.76	0.16	21.26	17.7
**20_14**	1.00176	1.78926	0.226	0.037	0.069	0.5	-0.595	-7.702	16.74	-10.87	7.65	23.45
**4_15**	1.32803	1.79772	0.007	0.073	0.088	0.883	-0.597	-8.138	8.32	-3.18	0.82	16.39
**18_22**	1.24367	1.84995	-0.116	0.083	0.074	0.549	-0.571	-7.564	32.62	-8.56	17.16	22.33
**18_9**	1.26158	1.83808	-0.217	0.112	0.154	0.565	-0.565	-8.616	34.38	-2.77	25.24	27.8
**19_18**	1.26662	1.85062	-0.094	0.09	0.092	0.546	-0.583	-7.419	32.45	-9.84	15.46	24.55
**16_18**	1.27163	1.84856	-0.093	0.089	0.088	0.55	-0.596	-7.364	32.83	-9.43	17.63	23.36
**5_12**	1.26993	1.7629	0.194	0.052	0.119	0.485	-0.54	-8.16	15.38	-2.63	7.33	13.84
**5_22**	1.30518	1.75908	-0.143	0.102	0.141	0.549	-0.508	-7.796	31.63	-1.99	20.87	16.56
**19_20**	1.25563	1.85164	-0.09	0.084	0.071	0.542	-0.586	-6.924	28.81	-11.16	10.23	23.72
**22_2**	1.27863	1.87981	-0.104	0.1	0.051	0.505	-0.621	-7.554	32.56	-10.32	16.48	18.34
**4_8**	1.36236	1.78301	-0.047	0.102	0.224	0.902	-0.542	-9.61	35.94	7.29	47.63	33.35
**22_10**	1.28808	1.8791	-0.131	0.114	0.086	0.522	-0.609	-7.323	35.21	-6.43	16.3	19.39
**8_13**	1.16339	1.71765	0.189	0.075	0.164	0.586	-0.523	-8.171	17	5.14	7.6	14.31
**15_6**	1.33134	1.84095	-0.217	0.034	0.045	0.669	-0.658	-7.652	26.91	-12.41	24.64	24.18
**22_18**	1.28932	1.85487	-0.099	0.113	0.106	0.524	-0.61	-7.333	30.16	-8.81	13.25	17.83
**6_22**	1.30577	1.73758	-0.143	0.103	0.147	0.512	-0.497	-7.83	31.79	-1.04	18.48	16.24
**12_8**	1.34647	1.81547	-0.01	0.1	0.19	0.812	-0.595	-9.173	33.76	6.5	39.16	25.66
**5_9**	1.3695	1.78253	-0.238	0.12	0.204	0.533	-0.516	-9.063	35.9	4.55	43.02	29.04
**16_10**	1.24298	1.8759	-0.124	0.091	0.076	0.546	-0.599	-7.388	34.73	-10.63	18.17	24.73
**11_6**	1.34773	1.84615	-0.211	0.059	0.115	0.461	-0.555	-8.074	29.28	-8.43	27.91	21
**16_19**	1.21991	1.86263	-0.105	0.089	0.083	0.548	-0.599	-7.466	30.83	-10.7	16.14	28.45
**18_11**	1.23066	1.84533	-0.165	0.101	0.11	0.555	-0.577	-7.803	29.18	-7.46	17.63	24.34
**18_4**	1.25613	1.85282	0.358	0.003	0.072	0.476	-0.581	-8.073	15.51	-11.81	24.44	20.68
**20_16**	1.25733	1.85283	-0.093	0.084	0.065	0.545	-0.601	-7.2	31.48	-11.12	16.5	24.13

**11_8**	1.33045	1.85922	0.02	0.097	0.206	0.48	-0.515	-9.297	33.6	-3.03	37.7	30.84
**6_13**	1.11887	1.68393	0.189	0.056	0.14	0.441	-0.523	-8.107	16.81	-0.24	7.85	12.9
**10_15**	1.24102	1.87659	0.092	0.046	0.04	0.49	-0.613	-7.346	7.94	-17	-0.69	29.07
**20_5**	1.28453	1.84251	-0.133	0.047	0.086	0.519	-0.587	-7.736	28.8	-9.57	22.94	23.82
**18_16**	1.24499	1.85435	-0.095	0.086	0.078	0.545	-0.592	-7.31	31.15	-9.83	15.16	22.87
**1_20**	1.26947	1.84736	-0.105	0.089	0.078	0.365	-0.602	-6.896	27.75	-16.71	15.16	28.13
**17_17**	1.27046	1.85685	-0.093	0.083	0.074	0.546	-0.596	-7.255	31.21	-10.21	15.68	23.65
**22_12**	1.26537	1.86572	0.225	0.06	0.069	0.472	-0.607	-7.624	16.34	-11.78	4.6	20.62
**19_16**	1.21716	1.86504	-0.091	0.089	0.088	0.545	-0.586	-7.307	31.44	-10.58	13.77	23.14
**17_20**	1.27064	1.84832	-0.09	0.081	0.063	0.543	-0.598	-6.91	29.23	-11.81	11.09	28.24
**5_16**	1.29818	1.77264	-0.122	0.102	0.137	0.539	-0.542	-7.564	29.82	-2.77	16.61	16.44
**6_17**	1.29836	1.75353	-0.121	0.102	0.142	0.495	-0.534	-7.554	28.61	-1.42	13.08	16.4
**17_4**	1.21841	1.86043	0.349	0.003	0.074	0.479	-0.582	-8.05	19.71	-7.72	23.32	19.8
**14_17**	1.30097	1.82946	-0.119	0.094	0.091	0.826	-0.623	-7.276	28.18	-4.68	20.04	13.91
**8_9**	1.37494	1.80252	-0.242	0.135	0.225	0.651	-0.497	-9.249	38.3	12.84	45.75	35.34
**3_11**	1.3083	1.87787	-0.165	0.098	0.087	0.581	-0.582	-7.706	29.96	-5.26	14.13	21.3
**8_20**	1.29492	1.79255	-0.118	0.117	0.155	0.627	-0.539	-7.226	24.23	0.13	7.6	14.76
**12_2**	1.29096	1.79232	-0.126	0.083	0.029	0.809	-0.652	-7.618	32.91	-4.25	27.62	12.33
**1_5**	1.34307	1.58698	-0.161	0.055	0.101	0.346	-0.58	-8.046	27.56	-12.73	22.52	24.14
**19_12**	1.13723	1.86443	0.231	0.037	0.064	0.494	-0.586	-7.764	16.01	-12.92	4.68	22.58
**13_4**	1.28562	1.79809	0.322	0.009	0.077	0.759	-0.606	-8.153	17.9	-3.87	23.11	11.77
**2_15**	1.30437	1.73717	0.051	0.079	0.053	0.534	-0.631	-7.644	9.88	-18.27	0.94	31.32
**6_18**	1.30061	1.74512	-0.124	0.104	0.148	0.495	-0.532	-7.716	31.97	0.43	16.01	17.33
**6_9**	1.36905	1.76551	-0.237	0.121	0.208	0.486	-0.508	-9.079	36.4	5.01	34.34	29.92
**1_22**	1.27863	1.86481	-0.129	0.091	0.09	0.369	-0.57	-7.509	30.97	-16.27	19.48	27.75
**2_6**	1.34601	1.85898	-0.218	0.051	0.084	0.531	-0.593	-7.892	27.77	-10.93	25.53	21.86
**17_7**	1.29924	1.85445	-0.225	0.089	0.116	0.546	-0.584	-8.585	37.49	-5.94	30.9	29.6
**16_22**	1.27519	1.85569	-0.115	0.082	0.068	0.55	-0.574	-7.504	32.39	-9.59	16.99	22.77
**13_6**	1.37546	1.82235	-0.249	0.06	0.099	0.801	-0.607	-7.991	26.88	-2.58	29.88	12.49
**5_1**	1.32021	1.66853	-0.361	0.096	0.112	0.514	-0.541	-8.573	38.69	-2.27	34.54	19.65
**15_1**	1.26086	1.75853	-0.338	0.062	0.005	0.669	-0.677	-7.768	33.8	-18.37	37.33	27.54
**8_16**	1.29975	1.80046	-0.123	0.12	0.161	0.63	-0.535	-7.722	29.26	1.75	13.63	17.93
**19_10**	1.19529	1.87463	-0.124	0.092	0.082	0.544	-0.586	-7.437	34.75	-10.46	18.87	25.13
**21_7**	1.28524	1.8506	-0.224	0.09	0.128	0.544	-0.573	-8.842	38.27	-3.93	32.06	31.01
**15_7**	1.32037	1.84347	-0.232	0.078	0.079	0.686	-0.666	-8.325	34.67	-10.38	36.35	28.62
**10_2**	1.25692	1.83616	-0.089	0.076	0.035	0.501	-0.622	-7.642	33.77	-11.25	19.89	21.65
**2_19**	1.28581	1.87072	-0.104	0.092	0.088	0.558	-0.615	-7.305	27.35	-17.18	15.68	27.63
**3_17**	1.26976	1.86764	-0.088	0.084	0.063	0.574	-0.601	-7.201	30.96	-7.68	9.09	22.13
**14_20**	1.29626	1.83272	-0.116	0.092	0.081	0.825	-0.624	-6.888	30.3	-2.79	17.66	15.55
**18_6**	1.27019	1.84285	-0.215	0.052	0.097	0.521	-0.576	-7.96	30.47	-7.12	23.79	22.86
**21_11**	1.25945	1.85282	-0.163	0.101	0.117	0.552	-0.572	-7.886	29.54	-6.37	17.73	24.08
**22_1**	1.30881	1.88611	-0.339	0.099	0.061	0.499	-0.611	-7.948	38.11	-6.87	27.11	19.53
**1_15**	1.30705	1.60712	0.036	0.079	0.073	0.347	-0.622	-7.776	8.58	-20.26	1.27	32.87
**15_20**	1.27114	1.79598	-0.09	0.063	0.015	0.692	-0.671	-6.671	31.09	-15.32	21.43	26.58

**10_6**	1.34102	1.87433	-0.208	0.05	0.089	0.491	-0.593	-7.903	29.56	-4.27	20.21	16.21
**19_2**	1.17572	1.87548	-0.09	0.075	0.038	0.529	-0.593	-7.706	33.64	-12.77	18.61	22.31
**11_7**	1.33446	1.8494	-0.213	0.096	0.138	0.486	-0.555	-8.888	37.73	-6.95	39.55	32.99
**7_21**	1.28903	1.85868	-0.108	0.094	0.151	0.451	-0.552	-7.959	33.5	2.61	15.36	21.47
**9_12**	1.24352	1.83198	0.244	0.047	0.131	0.401	-0.547	-8.253	14.68	-7.32	5.48	19.69
**2_3**	1.25833	1.87996	-0.088	0.076	0.026	0.547	-0.626	-7.615	30.53	-17.69	23.62	32.46
**10_1**	1.28784	1.74783	-0.323	0.076	0.047	0.492	-0.615	-8.042	36.3	-10.72	25.35	20.6
**16_1**	1.25601	1.88407	-0.33	0.077	0.049	0.522	-0.602	-8.044	37.08	-12.26	27.3	23.12
**8_8**	1.36179	1.78952	-0.024	0.11	0.253	0.627	-0.469	-9.804	38.57	14.21	54.03	40.72
**13_18**	1.30374	1.83252	-0.122	0.098	0.098	0.828	-0.622	-7.408	31.97	-3.19	24.25	14.37
**13_21**	1.30675	1.83252	-0.125	0.101	0.118	0.831	-0.618	-7.666	34.1	-1.12	26.21	13
**20_15**	1.17474	1.85764	0.081	0.05	0.037	0.521	-0.607	-7.357	8.51	-18.77	-0.25	31.13
**14_14**	1.02509	1.79485	0.194	0.047	0.084	0.78	-0.611	-7.891	17.74	-3.47	16.6	13.32
**7_18**	1.28447	1.84893	-0.103	0.092	0.135	0.447	-0.558	-7.684	29.82	-0.33	10.16	17.96
**7_1**	1.30261	1.73621	-0.343	0.083	0.099	0.422	-0.562	-8.519	39.54	1.38	26.48	18.75
**15_9**	1.32397	1.84711	-0.222	0.098	0.118	0.702	-0.654	-8.331	31.53	-9.86	30.52	29.47
**20_12**	1.19019	1.84104	0.233	0.034	0.05	0.496	-0.606	-7.566	14.61	-14.27	6.25	25.52
**6_11**	1.33594	1.76002	-0.19	0.114	0.164	0.493	-0.518	-8.162	29.95	1.8	20.59	18.93
**2_10**	1.2748	1.86239	-0.13	0.091	0.069	0.556	-0.613	-7.374	33.41	-13.47	19.09	24.09
**9_1**	1.29231	1.65804	-0.316	0.089	0.117	0.43	-0.546	-8.635	41.37	-5.5	36.59	25.34
**6_5**	1.36828	1.76418	-0.176	0.065	0.16	0.456	-0.518	-8.433	29.07	1.68	25.37	14.71
**4_19**	1.32168	1.80688	-0.158	0.104	0.128	0.912	-0.595	-7.573	28.6	-3	22.97	19.19
**5_19**	1.31069	1.77676	-0.128	0.103	0.151	0.538	-0.544	-7.633	28.08	-4.25	16.64	22.07
**15_18**	1.2783	1.79396	-0.096	0.071	0.037	0.694	-0.67	-7.139	34.21	-13	25.46	25.24
**20_3**	1.20752	1.85687	-0.082	0.074	0.027	0.537	-0.616	-7.514	32.34	-14.51	17.38	32.64
**4_10**	1.31009	1.77427	-0.183	0.103	0.114	0.908	-0.595	-7.696	34.5	-0.68	27.06	15.95
**21_6**	1.2953	1.85013	-0.216	0.052	0.104	0.518	-0.571	-8.051	31.12	-5.81	23.69	22.15
**13_7**	1.36065	1.82198	-0.253	0.099	0.129	0.823	-0.61	-8.502	36.22	1.83	42.7	22.9
**14_21**	1.30716	1.83125	-0.126	0.101	0.12	0.831	-0.617	-7.685	35.47	0.49	27.91	14.84
**20_9**	1.29114	1.83632	-0.216	0.108	0.145	0.563	-0.577	-7.918	33.41	-4.99	24.08	27.74
**7_9**	1.35743	1.8628	-0.228	0.111	0.196	0.466	-0.523	-9.061	35.84	8	41.05	31.09
**9_15**	1.28639	1.85994	0.086	0.068	0.118	0.429	-0.551	-8.239	6.28	-10.99	-0.94	26.53
**19_21**	1.24655	1.85722	-0.097	0.094	0.113	0.551	-0.578	-7.662	34.67	-8.1	17.81	25.73
**12_3**	1.28528	1.84377	-0.113	0.082	0.027	0.818	-0.653	-7.609	31.41	-4.57	23.73	18.06
**19_17**	1.21773	1.86238	-0.089	0.088	0.088	0.545	-0.587	-7.283	30.29	-9.46	11.29	21.55
**6_20**	1.28265	1.74344	-0.125	0.097	0.128	0.494	-0.533	-7.212	27.74	-0.87	10.56	16.82
**15_17**	1.27448	1.81515	-0.093	0.067	0.026	0.693	-0.67	-7.005	32.67	-14.38	24.23	26.36
**9_19**	1.25945	1.84675	-0.088	0.098	0.146	0.452	-0.545	-7.826	29.57	-5.25	15.02	27.25
**1_6**	1.35103	1.74616	-0.241	0.058	0.099	0.348	-0.579	-7.985	29.08	-11.92	24.98	23.57
**15_10**	1.25718	1.85648	-0.128	0.075	0.034	0.692	-0.675	-7.17	34.7	-15.26	30.63	26.88
**21_3**	1.20738	1.86244	-0.083	0.077	0.049	0.534	-0.602	-7.795	31.79	-12.08	17.11	31.66
**17_10**	1.24375	1.8706	-0.123	0.09	0.077	0.545	-0.6	-7.408	34.46	-11.13	18.83	25.72
**21_21**	1.26735	1.85508	-0.097	0.092	0.116	0.55	-0.582	-7.731	34.52	-6.55	19.3	25.09
**6_12**	1.27734	1.74757	0.194	0.054	0.125	0.442	-0.531	-8.19	15.42	-1.62	5.48	13.29

**5_14**	1.06556	1.65474	0.189	0.055	0.136	0.485	-0.532	-8.03	16.69	-0.69	9.81	13.41
**15_21**	1.28102	1.80241	-0.1	0.076	0.06	0.695	-0.667	-7.38	36.55	-12.63	28.2	26.26
**11_19**	1.28271	1.86056	-0.085	0.096	0.119	0.487	-0.575	-7.443	28.2	-13.57	18.17	29.08
**16_8**	1.28649	1.85974	0.01	0.09	0.182	0.539	-0.543	-8.772	33.37	-2.5	34.64	22.45
**18_13**	1.03654	1.78222	0.225	0.04	0.082	0.5	-0.584	-7.923	16.56	-9.98	6.81	22.96
**11_14**	1.03191	1.69035	0.235	0.047	0.101	0.439	-0.563	-7.905	16.95	-10.37	10.44	21.82
**9_16**	1.26748	1.84677	-0.076	0.094	0.145	0.45	-0.547	-7.653	29.48	-5.65	14.04	22.24
**18_5**	1.25561	1.84395	-0.136	0.049	0.1	0.52	-0.576	-8.021	29.22	-7.22	22.22	22.35
**22_21**	1.29273	1.862	-0.101	0.118	0.121	0.529	-0.604	-7.576	32.15	-7.15	15.35	18.28
**3_6**	1.34284	1.86928	-0.208	0.047	0.073	0.548	-0.58	-7.854	30.44	-2.65	18.68	17.64
**16_9**	1.31045	1.85797	-0.217	0.112	0.149	0.565	-0.57	-8.579	33.71	-3.86	24.84	27.67
**12_18**	1.30073	1.82764	-0.121	0.096	0.087	0.827	-0.642	-7.331	31.85	-2.06	22.79	11.88
**22_22**	1.29091	1.86759	-0.117	0.109	0.098	0.521	-0.575	-7.404	28.24	-9.98	13.87	18.64
**12_21**	1.30314	1.8338	-0.123	0.1	0.107	0.83	-0.639	-7.587	34.06	-0.67	27.36	12.32
**7_19**	1.26769	1.86338	-0.116	0.092	0.129	0.446	-0.558	-7.76	29.85	0.67	10.51	22.12
**17_22**	1.2731	1.85408	-0.114	0.081	0.068	0.55	-0.575	-7.524	32.57	-9.49	17.34	22.75
**11_2**	1.26108	1.87312	-0.083	0.085	0.063	0.468	-0.585	-7.828	33.12	-13.89	24.61	24.48
**10_8**	1.32362	1.86013	0.017	0.091	0.184	0.507	-0.557	-9.095	32.6	-1.62	35.06	25.21
**20_17**	1.26116	1.85046	-0.092	0.082	0.065	0.545	-0.602	-7.182	30.88	-11.4	15.79	23.85
**21_12**	1.18832	1.8512	0.232	0.037	0.073	0.492	-0.591	-7.867	14.59	-11.01	4.35	23.78
**8_14**	1.09675	1.68838	0.188	0.075	0.166	0.586	-0.523	-8.089	16.48	4.86	7.96	14.33
**19_6**	1.25883	1.85939	-0.216	0.053	0.097	0.52	-0.567	-7.957	30.76	-7.75	27.66	19.91
**16_15**	1.17903	1.87588	0.081	0.051	0.044	0.521	-0.602	-7.423	8	-17.37	-0.33	30.4
**18_2**	1.1907	1.85121	-0.09	0.074	0.038	0.529	-0.603	-7.706	33.57	-12.93	21.38	25.72
**21_20**	1.26615	1.85066	-0.089	0.083	0.078	0.54	-0.59	-6.978	28.95	-10.38	9.41	26.46
**11_10**	1.27342	1.87447	-0.111	0.097	0.101	0.483	-0.573	-7.531	33.83	-11.58	21.64	25.49
**8_7**	1.36293	1.80323	-0.255	0.118	0.194	0.633	-0.511	-9.401	43.79	10.38	48.59	35.67
**6_7**	1.35807	1.76047	-0.252	0.104	0.176	0.477	-0.518	-9.219	40.37	3.76	40.08	31.36
**4_18**	1.31204	1.78214	-0.153	0.102	0.126	0.911	-0.593	-7.627	32.57	0.27	24.66	15.12
**10_22**	1.27005	1.87131	-0.103	0.085	0.081	0.516	-0.58	-7.459	29.46	-11.08	17.67	21.36
**12_22**	1.30033	1.83493	-0.137	0.095	0.084	0.824	-0.616	-7.427	30.47	-3.76	25.81	13.51
**11_9**	1.34707	1.84622	-0.204	0.118	0.17	0.505	-0.541	-8.781	34.42	-4.56	31.19	29.27
**22_15**	1.26851	1.87868	0.073	0.075	0.05	0.496	-0.608	-7.411	9.06	-14.68	-1.07	26.5
**4_1**	1.33285	1.61667	-0.393	0.091	0.092	0.881	-0.599	-8.501	41.14	-1.34	49.03	16.34
**7_5**	1.35111	1.86672	-0.157	0.053	0.147	0.421	-0.54	-8.413	29.03	3.07	23.47	16.09
**1_2**	1.26696	1.59646	-0.117	0.083	0.042	0.355	-0.61	-7.705	32.33	-18.29	19.69	27.74
**4_14**	1.10168	1.66551	0.158	0.054	0.114	0.865	-0.584	-8.024	18.04	0.75	16.77	11.41
**9_4**	1.26423	1.83071	0.361	0.018	0.149	0.385	-0.529	-8.623	21.64	-2.21	29.46	15.51
**22_14**	1.09289	1.76673	0.217	0.063	0.088	0.477	-0.595	-7.76	18.26	-9.51	7.47	19.19
**7_8**	1.34142	1.85976	-0.006	0.088	0.229	0.441	-0.499	-9.603	34.78	9.27	49.73	33.73
**7_14**	1.01178	1.70383	0.209	0.045	0.131	0.399	-0.548	-8.025	16.48	0.8	1.92	14.3
**1_7**	1.33799	1.60546	-0.245	0.096	0.122	0.37	-0.581	-8.771	38.11	-9.45	35.35	31.47
**16_2**	1.26261	1.88114	-0.088	0.077	0.03	0.526	-0.617	-7.628	28	-18.78	20.94	25.81
**20_20**	1.26418	1.84442	-0.088	0.08	0.056	0.543	-0.603	-6.836	29.33	-12.39	11.08	27.82

**18_14**	0.99917	1.76106	0.224	0.04	0.083	0.5	-0.584	-7.868	16.41	-9.52	6.95	23.59
**11_11**	1.31025	1.85143	-0.151	0.107	0.128	0.493	-0.556	-7.911	28.86	-9.04	20.59	23.66
**22_5**	1.35368	1.86878	-0.135	0.07	0.105	0.495	-0.589	-7.879	29.31	-3.28	21	14.48
**5_20**	1.28262	1.75922	-0.124	0.096	0.123	0.536	-0.543	-7.183	27.53	-3.65	13.88	18.34
**2_9**	1.34543	1.87207	-0.223	0.11	0.147	0.574	-0.583	-8.592	31.9	-6.47	35.98	26.74
**10_17**	1.26558	1.8698	-0.081	0.085	0.08	0.516	-0.614	-7.23	30.88	-9.29	13.2	21.54
**1_8**	1.33053	1.59653	-0.01	0.097	0.193	0.359	-0.542	-9.202	33.04	-6.08	38.59	32.27
**14_22**	1.27935	1.82045	-0.141	0.095	0.09	0.825	-0.6	-7.576	31.48	-1.98	25.9	14.58
**16_20**	1.27102	1.84831	-0.091	0.082	0.063	0.544	-0.598	-6.894	29.35	-11.14	10.13	24.16
**10_11**	1.30509	1.87538	-0.158	0.1	0.103	0.524	-0.595	-7.755	29.06	-7.13	16.21	20.45
**2_12**	1.23156	1.83285	0.225	0.033	0.042	0.507	-0.604	-7.647	15.73	-15.5	6.72	24.72
**13_11**	1.33669	1.81421	-0.191	0.109	0.117	0.831	-0.61	-7.84	27.97	-1.53	23.75	13.37
**14_15**	1.26888	1.8139	0.043	0.067	0.058	0.801	-0.621	-7.704	8.16	-6.99	2.05	18.96
**6_10**	1.3	1.76538	-0.154	0.103	0.14	0.491	-0.533	-7.78	34.17	-2.14	20.74	19.3
**10_13**	1.10279	1.75187	0.232	0.039	0.072	0.47	-0.6	-7.824	16.92	-9.25	5.71	19.8
**3_22**	1.27199	1.86882	-0.108	0.084	0.064	0.571	-0.567	-7.423	29.61	-9.41	11.4	20.28
**18_8**	1.2317	1.8474	0.009	0.09	0.187	0.539	-0.538	-8.661	33.46	-1.44	34.89	19.12
**8_21**	1.30836	1.79682	-0.128	0.122	0.181	0.636	-0.527	-8.131	32.04	4.02	19.64	21.79
**22_13**	1.16662	1.79273	0.218	0.063	0.086	0.477	-0.595	-7.77	18.31	-4.68	2.05	14.3
**5_15**	1.32084	1.77292	0.034	0.076	0.108	0.526	-0.54	-8.195	8.28	-3.84	-0.73	18.84
**12_10**	1.29795	1.81966	-0.15	0.097	0.074	0.823	-0.643	-7.359	34.57	-1.93	25.44	12.12
**10_21**	1.27348	1.86335	-0.089	0.092	0.106	0.521	-0.607	-7.625	33.95	-7.61	17.15	21.44
**7_3**	1.26628	1.87128	-0.097	0.078	0.087	0.436	-0.573	-8.07	32.21	-1.99	17.41	22.16
**5_7**	1.35862	1.77736	-0.252	0.104	0.171	0.524	-0.527	-9.207	39.89	2.81	42.46	29.43
**18_7**	1.26143	1.84272	-0.225	0.09	0.121	0.546	-0.579	-8.592	38	-4.78	31.66	30.39
**2_20**	1.26501	1.84938	-0.094	0.081	0.057	0.555	-0.607	-6.843	29.71	-14.09	12.66	25.77
**5_13**	1.13877	1.6777	0.19	0.055	0.134	0.486	-0.532	-8.105	16.77	-1.49	10.48	13.89
**8_6**	1.3794	1.80423	-0.255	0.086	0.176	0.605	-0.516	-8.529	32.94	8.48	32.72	22.14
**12_19**	1.29153	1.84004	-0.132	0.096	0.081	0.825	-0.643	-7.379	31.17	-2.47	22.38	15.02
**11_22**	1.27091	1.85167	-0.101	0.091	0.099	0.486	-0.549	-7.656	32.26	-11.12	21.73	25.05
**11_3**	1.25517	1.86175	-0.07	0.083	0.062	0.474	-0.586	-7.822	31.52	-13.11	20.61	30.22
**2_16**	1.27168	1.8669	-0.096	0.089	0.074	0.555	-0.615	-7.246	29.48	-16.16	16.69	26.08
**1_14**	1.07891	1.63876	0.201	0.045	0.083	0.323	-0.589	-7.833	17.46	-14.46	7.7	25.86
**9_3**	1.25456	1.85302	-0.069	0.086	0.107	0.442	-0.559	-8.182	30.82	-8.27	19.84	27.82
**19_14**	0.88726	1.74883	0.226	0.045	0.086	0.497	-0.583	-7.804	18.49	-9.74	7.4	24.69
**5_4**	1.29004	1.74791	0.316	0.022	0.136	0.456	-0.527	-8.517	20.21	0.06	26.16	10.98
**12_15**	1.29358	1.83081	0.048	0.061	0.038	0.8	-0.642	-7.535	8.87	-9.18	2.78	19.34
**18_21**	1.25758	1.85112	-0.099	0.091	0.108	0.552	-0.586	-7.654	33.66	-6.97	18.69	23.78
**11_21**	1.27896	1.85107	-0.084	0.099	0.132	0.49	-0.567	-7.754	33.28	-10.24	20.79	25.82
**4_17**	1.30909	1.80297	-0.15	0.099	0.118	0.909	-0.595	-7.465	28.97	-2.15	21.57	14.61
**1_9**	1.34982	1.58684	-0.236	0.118	0.156	0.387	-0.566	-8.693	34.13	-8.04	35.57	30.92
**3_1**	1.2927	1.79911	-0.331	0.075	0.03	0.549	-0.603	-7.963	35.24	-15.24	27.2	25.62
**5_11**	1.33626	1.77852	-0.19	0.114	0.159	0.537	-0.527	-8.138	29.85	0.52	21.57	17.78
**8_17**	1.30007	1.79899	-0.121	0.118	0.162	0.629	-0.536	-7.682	28.65	2.82	12.5	18.15

**15_11**	1.29187	1.85063	-0.168	0.087	0.065	0.699	-0.66	-7.54	28.65	-13.51	25.21	27.14
**6_8**	1.35521	1.75774	-0.02	0.098	0.241	0.469	-0.481	-9.618	36.5	7.56	44.5	33.07
**2_21**	1.27957	1.85771	-0.102	0.094	0.102	0.561	-0.608	-7.618	31.43	-13.35	19.12	24.8
**7_13**	1.09702	1.7428	0.211	0.044	0.128	0.399	-0.548	-8.08	16.73	0.62	2.66	14.01
**20_10**	1.23001	1.85777	-0.122	0.089	0.069	0.545	-0.605	-7.318	35.07	-11.52	18.63	25.9
**22_4**	1.27273	1.87346	0.343	0.027	0.085	0.456	-0.589	-7.94	21.57	-3.76	22.24	12.27
**3_20**	1.26486	1.85643	-0.085	0.082	0.054	0.572	-0.603	-6.834	27.92	-13.58	9.46	26.07
**19_15**	1.11553	1.87668	0.081	0.052	0.05	0.519	-0.588	-7.457	7.69	-16.92	0.59	28.96
**14_3**	1.28919	1.82042	-0.117	0.085	0.044	0.818	-0.632	-7.75	30.42	-5.79	24.55	19.5
**14_5**	1.36832	1.809	-0.168	0.058	0.103	0.799	-0.606	-8.08	25.44	-1.92	28.07	11.3
**10_12**	1.23079	1.86983	0.239	0.034	0.055	0.465	-0.611	-7.697	15.43	-11.78	4.71	21
**13_3**	1.28882	1.82461	-0.117	0.084	0.042	0.819	-0.633	-7.725	30.32	-6.33	24.79	20.12
**9_11**	1.31242	1.84677	-0.148	0.11	0.165	0.462	-0.526	-8.219	28.71	-2.85	19.54	22.39
**11_20**	1.26475	1.84196	-0.072	0.09	0.098	0.481	-0.578	-6.956	26.79	-13.55	13.95	25.57
**1_3**	1.26064	1.68804	-0.103	0.081	0.041	0.36	-0.612	-7.706	30.91	-18.02	16.05	32.75
**15_16**	1.27231	1.82495	-0.094	0.068	0.026	0.693	-0.669	-7.022	33.52	-14.39	24.8	26.22
**10_3**	1.25116	1.88317	-0.075	0.074	0.033	0.507	-0.624	-7.642	31.81	-11.81	18.9	27.85
**2_2**	1.26428	1.71775	-0.102	0.078	0.027	0.54	-0.624	-7.609	30.67	-17.94	20.98	25.76
**4_6**	1.38613	1.8006	-0.288	0.067	0.131	0.888	-0.579	-8.281	29.67	1.79	35.16	14.19
**2_7**	1.33371	1.86615	-0.23	0.091	0.109	0.557	-0.596	-8.668	35.08	-10.11	36.91	30.66
**6_3**	1.28731	1.75275	-0.122	0.093	0.106	0.499	-0.54	-8.141	32.34	-1.21	18.63	20.21
**13_5**	1.36768	1.81256	-0.167	0.057	0.101	0.8	-0.607	-8.058	25.38	-2.61	28.61	11.58
**20_21**	1.26473	1.84884	-0.097	0.09	0.096	0.552	-0.596	-7.516	34.38	-8.31	17.36	23.69
**15_15**	1.19376	1.86041	0.074	0.035	-0.001	0.674	-0.675	-7.228	10.96	-22.19	5.28	32.55
**6_21**	1.30548	1.75446	-0.127	0.106	0.164	0.494	-0.528	-7.985	34.04	0.97	19.2	20.22
**7_10**	1.28393	1.86608	-0.136	0.091	0.126	0.444	-0.559	-7.743	34.82	1.37	15.02	19.91
**20_2**	1.21514	1.8514	-0.088	0.072	0.024	0.53	-0.613	-7.513	33.63	-14.43	18.07	26.6
**15_4**	1.21631	1.8338	0.34	-0.015	0.025	0.628	-0.659	-7.763	15.87	-13.68	17.35	23.86
**10_19**	1.2793	1.87681	-0.091	0.089	0.094	0.519	-0.614	-7.316	27.35	-12.89	16.89	25.11
**1_10**	1.27834	1.71913	-0.144	0.096	0.082	0.368	-0.598	-7.445	33.75	-16.31	19.18	27.79
**13_10**	1.30124	1.81063	-0.153	0.099	0.086	0.824	-0.623	-7.449	32.91	-4.13	26.49	14.28
**13_19**	1.31423	1.82597	-0.129	0.098	0.105	0.829	-0.624	-7.354	27.91	-6.11	21.56	16.6
**9_8**	1.33715	1.85025	0.017	0.095	0.24	0.452	-0.482	-9.681	35.9	4.46	44.49	34.76
**15_13**	1.16203	1.69313	0.221	0.021	0.021	0.647	-0.662	-7.465	15.58	-14.49	11.84	27.87
**21_2**	1.21305	1.86019	-0.089	0.075	0.046	0.527	-0.598	-7.802	33.5	-11.93	20.78	25.1
**20_19**	1.28458	1.85441	-0.105	0.086	0.073	0.546	-0.6	-7.343	31.15	-10.94	16.65	24.88
**13_20**	1.29593	1.83388	-0.116	0.092	0.08	0.825	-0.625	-6.873	27.87	-4.39	17.48	15.1
**9_5**	1.34427	1.85057	-0.136	0.061	0.163	0.429	-0.526	-8.502	28.6	-2.72	26.34	18.99
**22_17**	1.28606	1.86678	-0.095	0.11	0.099	0.521	-0.612	-7.189	28.17	-9.63	10.21	18.66
**4_21**	1.31622	1.80108	-0.156	0.107	0.141	0.915	-0.589	-7.901	33.59	0.08	28.21	16.97
**3_9**	1.34378	1.87368	-0.218	0.107	0.138	0.592	-0.571	-8.552	33.03	3.85	23.2	23.71
**14_10**	1.30158	1.80733	-0.153	0.099	0.088	0.824	-0.622	-7.469	33.14	-4.5	26.9	15.02
**2_5**	1.33863	1.87588	-0.14	0.049	0.087	0.53	-0.593	-7.95	26.36	-10.73	23.69	21.31
**14_9**	1.37072	1.8079	-0.241	0.121	0.165	0.838	-0.599	-8.436	32.67	2.47	35.6	23.64

**11_16**	1.26968	1.85732	-0.077	0.094	0.107	0.483	-0.575	-7.377	29.97	-12.55	17.48	24.94
**10_14**	1.05306	1.70566	0.231	0.039	0.073	0.47	-0.6	-7.771	16.96	-8.91	7.08	19.71
**3_5**	1.33549	1.87891	-0.13	0.045	0.076	0.546	-0.58	-7.911	28.47	0.72	13.55	14.76
**20_6**	1.29538	1.84265	-0.211	0.049	0.083	0.521	-0.587	-7.708	29.39	-9.29	24.19	23.21
**16_5**	1.30258	1.86381	-0.134	0.049	0.094	0.52	-0.581	-7.963	28.97	-7.96	21.42	22.5
**11_13**	1.11882	1.72079	0.236	0.047	0.099	0.439	-0.563	-7.944	16.84	-10.84	10.22	21.76
**18_17**	1.24928	1.852	-0.094	0.084	0.079	0.545	-0.593	-7.289	30.5	-9.86	14.78	21.95
**6_16**	1.29821	1.75418	-0.122	0.103	0.142	0.497	-0.532	-7.593	29.54	-1.91	14.48	16.38
**3_8**	1.32524	1.87079	0.011	0.088	0.173	0.561	-0.545	-9.048	32.6	5.46	39.64	25.76
**8_5**	1.37313	1.80292	-0.174	0.085	0.18	0.604	-0.515	-8.613	30.41	7.59	31.23	19.7
**1_4**	1.26271	1.59794	0.325	0.01	0.082	0.3	-0.583	-8.12	20.97	-13.75	28.45	24.26
**17_15**	1.18255	1.87031	0.081	0.05	0.045	0.521	-0.602	-7.442	8.52	-14.72	0.63	27.96
**21_13**	1.0242	1.81482	0.226	0.041	0.09	0.497	-0.58	-7.972	16.07	-9.22	7.11	23.12
**19_9**	1.25236	1.84767	-0.218	0.117	0.162	0.571	-0.548	-8.631	32.52	-5.26	25.14	28.75
**12_5**	1.36367	1.82672	-0.164	0.055	0.088	0.798	-0.627	-7.954	26.43	1.29	26.09	9.61
**18_20**	1.2573	1.84472	-0.09	0.082	0.07	0.543	-0.594	-6.916	29.08	-10.34	9.63	23.44
**10_16**	1.26513	1.8748	-0.083	0.087	0.08	0.516	-0.613	-7.256	29.9	-11.37	14.1	22.64
**1_17**	1.27475	1.86209	-0.109	0.091	0.087	0.367	-0.6	-7.274	30.95	-14.55	13.7	27.48
**17_8**	1.28136	1.85453	0.01	0.089	0.182	0.538	-0.543	-8.711	33.25	-1.98	36.15	30.18
**17_5**	1.30209	1.85776	-0.134	0.048	0.094	0.52	-0.581	-7.981	28.27	-8.23	21.52	22.88
**17_1**	1.25792	1.8748	-0.33	0.076	0.05	0.522	-0.603	-8.062	36.92	-11.62	26.58	22.51
**5_5**	1.36839	1.78386	-0.176	0.064	0.154	0.501	-0.527	-8.408	28.64	0.8	27.95	14.33
**5_8**	1.35536	1.77581	-0.02	0.098	0.237	0.515	-0.489	-9.622	36.36	5.5	44.25	32.66
**4_3**	1.29742	1.81112	-0.15	0.09	0.073	0.901	-0.606	-8.017	31.74	-3.6	26.52	20.35
**11_4**	1.25897	1.86827	0.36	0.011	0.1	0.418	-0.557	-8.229	20.71	-9.47	27.3	19.56
**16_21**	1.27554	1.85498	-0.098	0.092	0.105	0.553	-0.59	-7.607	34.52	-7.63	19.21	23.58
**17_21**	1.27489	1.85382	-0.098	0.091	0.104	0.553	-0.59	-7.622	34.64	-7.79	19.65	24.35
**11_1**	1.29207	1.68446	-0.317	0.084	0.076	0.461	-0.576	-8.249	37.13	-12.21	32.92	24.68
**21_5**	1.28721	1.85155	-0.136	0.05	0.108	0.517	-0.571	-8.115	29.57	-6.4	21.85	22.25
**20_7**	1.2871	1.84209	-0.223	0.088	0.108	0.544	-0.591	-7.875	37.16	-6.72	29.69	29.82
**6_2**	1.29244	1.76133	-0.133	0.094	0.108	0.486	-0.541	-8.153	33.75	-1.57	18.87	14.96
**12_11**	1.33333	1.8343	-0.189	0.107	0.105	0.829	-0.63	-7.753	29.02	0.42	22.75	11.98
**7_16**	1.28021	1.86512	-0.101	0.09	0.128	0.444	-0.56	-7.561	30.26	0.07	10.21	17.77
**20_13**	1.05812	1.80405	0.227	0.037	0.067	0.5	-0.595	-7.694	16.88	-11.13	7.65	23.31
**21_4**	1.20847	1.85121	0.349	0.006	0.089	0.476	-0.573	-8.196	19.57	-6.54	24.62	18.97
**11_12**	1.24231	1.83488	0.243	0.043	0.083	0.433	-0.575	-7.879	15.45	-12.99	9.14	23.19
**12_4**	1.28055	1.76996	0.324	0.005	0.063	0.758	-0.625	-8.04	18.62	-0.13	20.35	8.33
**4_22**	1.31025	1.79838	-0.172	0.099	0.118	0.908	-0.571	-7.763	32.43	-0.64	30.25	15.22
**17_6**	1.31309	1.85417	-0.213	0.05	0.091	0.521	-0.581	-7.922	30.06	-8.17	23.44	23.16
**18_18**	1.25435	1.84283	-0.094	0.089	0.093	0.549	-0.592	-7.406	32.48	-9.01	17.51	22.93
**5_3**	1.28697	1.77413	-0.121	0.092	0.099	0.538	-0.551	-8.103	32.35	-2.83	21.14	20.93
**16_16**	1.26901	1.86255	-0.094	0.086	0.074	0.546	-0.596	-7.258	31.53	-10.39	16.04	23.06
**4_12**	1.26561	1.77889	0.165	0.049	0.094	0.859	-0.593	-8.108	14.82	-2.09	14.26	12.23
**1_21**	1.2826	1.86109	-0.117	0.098	0.114	0.373	-0.591	-7.678	34.86	-12.7	19.15	27.41

**16_12**	1.19432	1.86204	0.232	0.036	0.058	0.496	-0.6	-7.711	15.25	-12.78	4.84	24.95
**1_16**	1.27409	1.86262	-0.11	0.093	0.087	0.367	-0.599	-7.301	31.3	-15.58	13.65	27.6
**13_15**	1.22071	1.81657	0.042	0.069	0.058	0.801	-0.622	-7.803	8.26	-7.23	2.45	18.96
**13_9**	1.37043	1.81164	-0.241	0.12	0.164	0.838	-0.6	-8.543	32.59	2.23	34.97	22.82
**4_16**	1.30851	1.80462	-0.151	0.1	0.118	0.909	-0.595	-7.5	30.11	-1.77	22.48	14.88
**8_12**	1.27423	1.79646	0.195	0.072	0.15	0.58	-0.534	-8.371	15.97	2.39	4.81	13.61
**2_17**	1.27228	1.86215	-0.095	0.087	0.074	0.555	-0.615	-7.22	28.57	-15.89	15.8	26.1
**3_10**	1.27425	1.88109	-0.126	0.088	0.058	0.574	-0.599	-7.34	35.31	-5.9	14.38	20.18
**13_14**	1.01248	1.78259	0.194	0.047	0.082	0.78	-0.612	-7.883	16.85	-5.02	14.5	13.73
**18_10**	1.20437	1.86313	-0.124	0.09	0.081	0.545	-0.595	-7.435	34.96	-10.37	18.36	25.62
**9_22**	1.27356	1.84315	-0.099	0.091	0.141	0.456	-0.52	-7.911	30.93	-5.53	18.44	21.8
**3_4**	1.25435	1.86619	0.35	-0.001	0.055	0.506	-0.579	-7.974	20.77	-7.91	20.64	19.67
**22_6**	1.36082	1.86208	-0.212	0.072	0.103	0.496	-0.589	-7.827	31.02	-3.11	22.29	14.69
**17_9**	1.30876	1.85208	-0.216	0.111	0.149	0.565	-0.57	-8.567	33.86	-3.7	25.05	27.81
**15_19**	1.28521	1.83457	-0.106	0.072	0.036	0.693	-0.667	-7.142	31.19	-14.38	24.13	27.15
**12_13**	1.1207	1.74413	0.197	0.043	0.065	0.779	-0.631	-7.835	16.03	-1.51	11.65	9.62
**4_4**	1.29988	1.62478	0.285	0.018	0.114	0.842	-0.577	-8.486	20.01	1.85	29.19	10.97
**20_11**	1.25754	1.84399	-0.163	0.099	0.097	0.554	-0.588	-7.634	29.35	-9.32	18.05	24.8
**22_8**	1.3401	1.86524	0.008	0.109	0.194	0.516	-0.548	-8.944	33.42	2.17	36.04	26.93
**12_17**	1.29737	1.83795	-0.117	0.092	0.077	0.825	-0.644	-7.181	31.09	-1.96	21.25	13.14
**12_9**	1.36703	1.82849	-0.24	0.119	0.154	0.836	-0.62	-8.645	33.52	4.16	36.5	15.07
**21_17**	1.26267	1.85609	-0.092	0.085	0.087	0.542	-0.588	-7.354	30.35	-9.44	12.59	26.66
**7_20**	1.27531	1.85415	-0.096	0.084	0.122	0.442	-0.564	-7.1	25.49	-5.69	7.23	21.53
**3_21**	1.27689	1.86186	-0.096	0.091	0.091	0.579	-0.595	-7.596	34.81	-4.74	13.84	21.49
**17_2**	1.26257	1.87577	-0.088	0.076	0.03	0.526	-0.618	-7.65	28.22	-18.21	20.76	25.64
**2_14**	1.05377	1.67011	0.217	0.038	0.062	0.512	-0.592	-7.769	17.1	-13.73	10.43	24.72
**9_21**	1.28035	1.8424	-0.082	0.101	0.167	0.458	-0.539	-8.036	32.47	-4.17	18.73	24.64
**15_12**	1.26151	1.8422	0.23	0.015	0	0.644	-0.669	-7.372	13.8	-16.78	10.8	26.01
**2_8**	1.32712	1.86598	0.006	0.092	0.181	0.545	-0.557	-9.091	32.02	-3.97	34.31	28.48
**11_5**	1.3404	1.85602	-0.13	0.057	0.118	0.461	-0.555	-8.14	27.94	-8.82	27.11	21.48
**3_16**	1.26917	1.8724	-0.089	0.086	0.063	0.573	-0.601	-7.225	30.31	-12.68	12.37	25.27
**5_2**	1.29239	1.77337	-0.132	0.093	0.102	0.526	-0.552	-8.113	33	-4.31	23.38	15.12
**13_13**	1.09279	1.80287	0.195	0.047	0.08	0.78	-0.612	-7.906	18.1	-3.38	13.68	12.68
**22_9**	1.35895	1.86243	-0.222	0.131	0.158	0.544	-0.577	-8.504	35.28	1.98	27.24	24.56
**15_14**	1.10354	1.6589	0.219	0.021	0.022	0.647	-0.661	-7.483	14.81	-15.27	11.99	25.81
**21_22**	1.26362	1.85339	-0.115	0.083	0.082	0.547	-0.565	-7.628	32.11	-7.92	16.02	21.52
**21_14**	0.97797	1.79907	0.225	0.041	0.091	0.497	-0.58	-7.908	15.73	-9.31	6.89	23.04
**22_16**	1.28553	1.87026	-0.096	0.111	0.098	0.521	-0.611	-7.213	28.92	-10.05	11.53	18.9
**10_4**	1.25136	1.81732	0.356	0.002	0.071	0.449	-0.594	-8.037	20.82	-6.85	22.39	16.33
**11_17**	1.27035	1.8543	-0.076	0.093	0.107	0.483	-0.576	-7.348	28.94	-12.71	16.89	25.06
**18_3**	1.18104	1.85753	-0.084	0.076	0.041	0.537	-0.607	-7.697	32.17	-13.22	17.58	32.59
**15_22**	1.27597	1.81374	-0.114	0.068	0.028	0.697	-0.655	-7.272	33.61	-14.52	28.3	26.56
**8_2**	1.29401	1.77855	-0.134	0.113	0.127	0.614	-0.548	-8.303	34.73	1.85	21.35	17.56
**21_15**	1.16889	1.8644	0.08	0.052	0.059	0.517	-0.593	-7.573	8.26	-16.99	-0.14	30.3

**5_17**	1.29289	1.76829	-0.125	0.097	0.133	0.536	-0.542	-7.585	29.87	-2.12	16.19	17.02
**14_19**	1.31446	1.8221	-0.129	0.098	0.106	0.829	-0.623	-7.372	27.9	-5.76	22.06	16.78
**7_22**	1.28695	1.86218	-0.123	0.088	0.127	0.448	-0.529	-7.77	30.42	-0.08	15.08	15.5
**17_14**	0.98443	1.79003	0.225	0.039	0.077	0.5	-0.589	-7.857	16.59	-10.18	7.64	23.62
**18_12**	1.15585	1.83461	0.231	0.036	0.064	0.495	-0.596	-7.768	14.83	-12.65	5.49	25
**9_20**	1.26388	1.83422	-0.069	0.091	0.14	0.448	-0.551	-7.162	24.73	-7.91	10.06	23.45
**2_4**	1.25754	1.69827	0.343	0.004	0.066	0.489	-0.594	-8.015	19.46	-12.81	25.27	22.05
**4_11**	1.34591	1.8055	-0.22	0.115	0.141	0.915	-0.581	-8.095	29.37	1.32	28.22	16.97
**3_2**	1.26269	1.87663	-0.097	0.074	0.015	0.559	-0.609	-7.569	32.29	-10.83	15.31	22.28
**14_4**	1.21468	1.80102	0.322	0.01	0.08	0.758	-0.605	-8.095	19.13	-2.02	23.2	11.12
**1_11**	1.31388	1.60861	-0.183	0.106	0.111	0.377	-0.581	-7.828	28.37	-11.7	17.07	25.16
**12_14**	1.06475	1.71018	0.196	0.043	0.066	0.779	-0.631	-7.802	16.06	-1.56	11.86	9.73
**6_14**	1.02509	1.66427	0.187	0.057	0.142	0.439	-0.523	-8.049	16.65	-0.43	8.21	13.45
**6_1**	1.31994	1.72915	-0.361	0.097	0.119	0.475	-0.53	-8.612	39.64	-1.26	29.68	19.46
**14_13**	1.07802	1.80691	0.195	0.047	0.082	0.78	-0.611	-7.925	17.61	-3.8	15.84	12.84
**10_10**	1.26882	1.86372	-0.118	0.09	0.075	0.515	-0.612	-7.386	34.13	-10.55	18.88	22.43
**7_11**	1.32087	1.86679	-0.175	0.103	0.15	0.455	-0.54	-8.135	29.03	4.96	18.27	19.81
**9_10**	1.27398	1.85862	-0.109	0.098	0.142	0.45	-0.546	-7.831	32.94	-6.3	19.55	24.64
**9_14**	1.04431	1.68641	0.235	0.051	0.147	0.407	-0.536	-8.061	15.97	-5.11	8	18.93
**13_12**	1.18023	1.81734	0.201	0.042	0.062	0.776	-0.62	-7.769	15.15	-5.46	13.21	14.11
**8_11**	1.33973	1.80498	-0.193	0.13	0.182	0.64	-0.515	-8.298	30.67	7.33	25.36	23.96
**8_10**	1.30215	1.79631	-0.156	0.122	0.155	0.629	-0.534	-7.924	34.34	4.82	20.4	21.96
**13_16**	1.30017	1.82901	-0.121	0.096	0.09	0.826	-0.623	-7.287	29.19	-5.06	21.93	14.64
**9_13**	1.12628	1.71969	0.237	0.051	0.145	0.407	-0.536	-8.139	16.42	-5.78	8.14	19.81
**9_6**	1.35131	1.8411	-0.218	0.063	0.16	0.43	-0.526	-8.422	30.45	-1.93	27.14	19.73
**7_6**	1.35834	1.86139	-0.239	0.056	0.143	0.422	-0.54	-8.329	30.61	3.62	25.46	18.21
**5_21**	1.30602	1.77159	-0.126	0.105	0.159	0.538	-0.538	-7.959	33.27	-1.25	21.71	19.54
**12_6**	1.37134	1.83871	-0.245	0.057	0.085	0.8	-0.627	-7.891	27.72	0.8	27.35	9.97
**10_18**	1.26964	1.85359	-0.085	0.089	0.088	0.518	-0.612	-7.377	31.13	-10.25	15.99	21.78
**8_1**	1.32307	1.687	-0.367	0.113	0.136	0.609	-0.535	-8.747	42.43	4.74	38.09	23.67
**22_19**	1.29139	1.86646	-0.113	0.107	0.094	0.523	-0.604	-7.409	32.35	-6.07	12.02	20.76
**20_4**	1.20243	1.84073	0.349	0.001	0.064	0.479	-0.588	-7.757	19.02	-9.18	24.26	20.6
**16_7**	1.30042	1.859	-0.225	0.09	0.115	0.547	-0.583	-8.639	37.47	-5.6	29.89	30.14
**14_18**	1.30408	1.83019	-0.123	0.098	0.1	0.828	-0.621	-7.429	30.57	-3.83	23.65	13.73
**12_20**	1.29277	1.8302	-0.114	0.09	0.067	0.824	-0.645	-6.811	27.89	-4.22	17.02	13.59
**6_15**	1.32192	1.74997	0.032	0.077	0.115	0.49	-0.529	-8.211	7.57	-4.53	-1.09	19.86
**2_1**	1.29366	1.66193	-0.335	0.077	0.041	0.531	-0.616	-8.016	34.77	-17.24	31.7	25.12
**5_6**	1.375	1.77637	-0.257	0.066	0.151	0.503	-0.527	-8.329	30.41	0.96	29.51	15.99
**9_18**	1.2739	1.82314	-0.076	0.098	0.152	0.453	-0.545	-7.76	30.73	-4.66	15.5	23.08
**14_2**	1.29488	1.80197	-0.129	0.086	0.046	0.81	-0.631	-7.76	31.92	-6.73	29.19	15.51
**15_2**	1.23816	1.85493	-0.096	0.056	-0.017	0.681	-0.679	-7.377	32.25	-17.59	33.5	27.04
**10_7**	1.32907	1.87713	-0.219	0.089	0.113	0.516	-0.595	-8.685	36.74	-4.53	39.01	26.12
**16_6**	1.31376	1.85826	-0.213	0.051	0.091	0.521	-0.581	-7.904	29.91	-8.45	23.78	23.27
**17_3**	1.22419	1.8672	-0.083	0.075	0.035	0.537	-0.611	-7.664	32.4	-12.76	16.53	31.62

**18_15**	1.14012	1.86007	0.081	0.051	0.05	0.52	-0.598	-7.47	7.89	-13.69	0.19	26.9
**21_18**	1.27198	1.84938	-0.093	0.089	0.099	0.545	-0.587	-7.484	32.77	-7.57	17.07	25.62
**13_2**	1.29455	1.80486	-0.128	0.086	0.044	0.81	-0.632	-7.736	32.48	-6.24	28.76	15.25
**13_17**	1.30063	1.83201	-0.119	0.094	0.089	0.827	-0.624	-7.257	30.25	-3.55	21.4	14.58
**13_8**	1.35052	1.80177	-0.012	0.101	0.199	0.814	-0.575	-8.559	32.99	4.79	43.17	27.06
**21_8**	1.27172	1.8499	0.008	0.09	0.194	0.537	-0.532	-9.24	33.72	-0.41	35.81	30.16
**2_18**	1.27573	1.82915	-0.098	0.09	0.083	0.557	-0.613	-7.366	31.06	-14.64	17.75	25.9
**7_4**	1.27174	1.84843	0.336	0.01	0.13	0.377	-0.542	-8.523	22.06	1.95	29.71	13.77
**15_5**	1.31371	1.8525	-0.138	0.032	0.046	0.667	-0.658	-7.705	24.74	-13.04	23.73	25.2
**17_16**	1.26798	1.86005	-0.093	0.085	0.073	0.546	-0.596	-7.276	31.56	-10.89	16.43	24.06
**9_7**	1.33713	1.84476	-0.212	0.1	0.176	0.456	-0.523	-9.269	41.69	2.02	50.93	30.79
**21_16**	1.25995	1.85806	-0.093	0.086	0.087	0.542	-0.587	-7.378	31.25	-9.34	13.19	22.66
**19_22**	1.19871	1.8583	-0.112	0.088	0.084	0.543	-0.571	-7.602	32.21	-10.19	17.63	22.47
**14_16**	1.3006	1.82538	-0.121	0.096	0.091	0.826	-0.622	-7.305	30.17	-3.74	21.01	14.63
**6_6**	1.37485	1.76017	-0.257	0.067	0.157	0.458	-0.519	-8.354	30.83	2.13	27.61	16.91
**3_7**	1.33245	1.87153	-0.225	0.087	0.1	0.573	-0.584	-8.626	37.58	2.29	31.41	25.44
**3_14**	1.03688	1.71416	0.224	0.037	0.057	0.528	-0.586	-7.754	18.11	-9.6	4.64	23.09
**3_3**	1.25668	1.88205	-0.082	0.073	0.014	0.565	-0.612	-7.58	33.92	-4.58	15.45	23.71
**2_22**	1.27099	1.86139	-0.12	0.082	0.062	0.558	-0.581	-7.485	30.71	-13.8	19.85	26.81
**21_1**	1.24308	1.86316	-0.33	0.078	0.063	0.519	-0.593	-8.203	37.51	-10.11	27.58	23.57
**4_13**	1.15464	1.68917	0.159	0.053	0.112	0.865	-0.584	-8.087	17.76	-0.93	17	12.03
**11_15**	1.30017	1.86018	0.072	0.083	0.088	0.458	-0.6	-7.891	8.83	-14.47	0.2	29.14
**22_20**	1.28108	1.85809	-0.092	0.108	0.091	0.519	-0.615	-6.827	26.1	-11.17	9.29	19.62
**22_3**	1.27265	1.87555	-0.09	0.098	0.051	0.511	-0.623	-7.563	31.36	-10.97	15.72	24.53
**8_3**	1.28753	1.80585	-0.123	0.109	0.125	0.623	-0.548	-8.282	31.89	2.11	21.47	20.67
**14_7**	1.36098	1.81838	-0.253	0.099	0.13	0.823	-0.609	-8.397	37.44	1.85	42.9	22.29
**17_11**	1.27507	1.85824	-0.164	0.1	0.105	0.555	-0.582	-7.772	29.18	-8.27	17.84	24.37
**22_11**	1.32423	1.86863	-0.171	0.121	0.117	0.532	-0.59	-7.673	30.39	-3.61	16.86	17.79
**10_9**	1.34148	1.86977	-0.211	0.109	0.15	0.534	-0.583	-8.597	33.18	-2.89	24.7	24.47
**1_13**	1.11502	1.67085	0.202	0.045	0.082	0.323	-0.589	-7.884	16.67	-16.02	9.01	26.5
**19_11**	1.22244	1.86407	-0.164	0.102	0.11	0.553	-0.568	-7.803	29.28	-8.04	21.52	22.54
**9_17**	1.26993	1.84511	-0.073	0.093	0.148	0.45	-0.548	-7.601	27.67	-6.88	14.18	23.06
**12_12**	1.25975	1.82665	0.203	0.039	0.046	0.775	-0.64	-7.684	15.04	-3.16	10.34	10.75
**16_13**	1.03868	1.79884	0.225	0.04	0.075	0.5	-0.589	-7.876	16.71	-11.15	7.68	24.3
**16_3**	1.22519	1.87101	-0.083	0.076	0.035	0.538	-0.611	-7.643	32.2	-13.76	17.6	33.03
**7_7**	1.3446	1.86325	-0.239	0.094	0.162	0.448	-0.538	-9.185	43.58	6.87	47.85	32.64
**3_12**	1.23533	1.87071	0.232	0.033	0.036	0.523	-0.597	-7.636	17.07	-13.73	4.59	25.51
**14_11**	1.33697	1.81014	-0.191	0.109	0.118	0.831	-0.609	-7.861	27.95	-1.7	24.02	13.25
**4_20**	1.30402	1.79461	-0.147	0.096	0.108	0.908	-0.597	-7.043	28.27	-1.03	17.33	15.18
**16_14**	0.9903	1.77477	0.224	0.04	0.077	0.5	-0.589	-7.839	16.56	-8.14	5.49	21.2
**16_11**	1.27717	1.86391	-0.164	0.101	0.104	0.555	-0.582	-7.755	29.31	-8.27	17.82	24.14
**21_19**	1.20497	1.85911	-0.104	0.09	0.096	0.544	-0.59	-7.581	30.63	-9.5	14.2	25.42
**17_13**	1.03923	1.80884	0.226	0.039	0.076	0.5	-0.589	-7.893	16.55	-10.72	7.31	23.58
**20_8**	1.25281	1.8423	0.011	0.088	0.174	0.537	-0.551	-7.956	32.76	-3.63	31.99	22.21

**14_8**	1.35125	1.79839	-0.013	0.101	0.2	0.814	-0.573	-8.451	33.05	4.81	43.76	27.45
**14_12**	1.21109	1.80951	0.2	0.042	0.062	0.776	-0.619	-7.822	13.61	-6.26	12.61	13.58
**2_11**	1.30995	1.87254	-0.17	0.101	0.098	0.564	-0.596	-7.741	27.19	-11.15	17.65	23.24
**15_3**	1.24477	1.85326	-0.088	0.058	-0.018	0.689	-0.682	-7.364	32.84	-15.81	26.81	29.52
**20_22**	1.25417	1.84709	-0.114	0.081	0.061	0.55	-0.579	-7.422	32.81	-10.28	17.4	23.2
**3_13**	1.10028	1.74906	0.226	0.036	0.055	0.528	-0.586	-7.773	18.1	-9.15	3.74	22.57
**3_15**	1.24251	1.88435	0.085	0.044	0.021	0.548	-0.599	-7.291	9.34	-18.09	-0.21	32.34
**8_18**	1.29493	1.78805	-0.129	0.118	0.161	0.632	-0.532	-7.894	31.85	4.54	17.6	21.28
**2_13**	1.12328	1.7005	0.218	0.037	0.059	0.512	-0.592	-7.771	16.9	-13.67	8.85	24.15
**6_4**	1.29048	1.76403	0.316	0.023	0.142	0.409	-0.518	-8.539	20.26	0.6	26.22	11.3
**20_1**	1.2473	1.85182	-0.329	0.075	0.041	0.521	-0.608	-7.723	35.53	-12.58	24.95	22.72
**19_13**	1.0067	1.80189	0.225	0.042	0.082	0.497	-0.575	-7.899	18.15	-9.44	6.32	24.36
**1_12**	1.21186	1.57961	0.208	0.041	0.064	0.318	-0.601	-7.766	15.15	-17.25	5.79	26.35
**19_3**	1.18296	1.86738	-0.08	0.081	0.047	0.534	-0.609	-7.68	31.97	-13.12	16.73	29.46
**16_4**	1.21851	1.86897	0.348	0.004	0.074	0.479	-0.582	-8.033	19.6	-8.44	23.8	20.1
**4_5**	1.37918	1.79384	-0.206	0.064	0.134	0.886	-0.578	-8.363	28.04	1.59	33.44	13.17
**19_19**	1.28741	1.86612	-0.106	0.09	0.087	0.545	-0.582	-7.47	30.25	-10.06	16.89	26.38
**7_12**	1.25212	1.85752	0.218	0.04	0.112	0.394	-0.56	-8.152	15.45	-3.71	0.69	16.7
**7_17**	1.28044	1.86232	-0.1	0.086	0.131	0.444	-0.561	-7.527	28.15	-1.3	7.6	17.39
**17_18**	1.27117	1.84861	-0.093	0.088	0.088	0.549	-0.596	-7.381	32.88	-9.57	17.67	24.03
**14_6**	1.37578	1.8187	-0.249	0.06	0.1	0.801	-0.606	-8.013	26.98	-1.06	28.81	11.05
**19_7**	1.26381	1.85662	-0.222	0.094	0.125	0.543	-0.581	-8.672	38.93	-4.54	30.73	30.84
**17_19**	1.21738	1.86062	-0.104	0.089	0.083	0.548	-0.6	-7.486	30.56	-10.42	16.3	25.19
**22_7**	1.34788	1.86215	-0.23	0.11	0.125	0.525	-0.59	-8.586	38.45	-0.25	31.09	25.68
**11_18**	1.27392	1.82915	-0.079	0.096	0.115	0.486	-0.573	-7.497	32.07	-10.49	18.48	24.43
**6_19**	1.3103	1.75837	-0.128	0.104	0.156	0.496	-0.534	-7.661	27.91	-3	14.87	21.27
**21_10**	1.23299	1.86347	-0.123	0.091	0.089	0.542	-0.591	-7.516	35	-8.96	17.72	24.4
**1_19**	1.2871	1.86228	-0.118	0.095	0.098	0.371	-0.598	-7.37	29.11	-17.31	15.56	30.58
**5_18**	1.30187	1.7575	-0.123	0.103	0.144	0.538	-0.541	-7.687	31.23	-2.23	18.85	17.6
**3_19**	1.25201	1.8681	-0.107	0.086	0.061	0.573	-0.598	-7.43	31.65	-4.73	8.21	20.59
**18_1**	1.21865	1.85311	-0.33	0.078	0.055	0.521	-0.598	-8.102	37.06	-11.29	27.59	23.14
**15_8**	1.29501	1.83883	0.009	0.078	0.152	0.676	-0.634	-8.574	29.01	-7.85	28.8	30.54
**21_9**	1.29316	1.84705	-0.216	0.112	0.16	0.563	-0.559	-8.746	34.47	-2.15	26.17	28.46
**8_22**	1.30955	1.79091	-0.147	0.117	0.158	0.636	-0.5	-7.931	31.94	3.57	18.18	16.32
**7_2**	1.27271	1.86272	-0.11	0.081	0.09	0.429	-0.572	-8.083	32.58	-2.17	15.63	15.39
**19_5**	1.24692	1.8641	-0.137	0.05	0.1	0.519	-0.567	-8.018	28.97	-8.26	25.74	19.96
**10_5**	1.33351	1.86944	-0.128	0.048	0.091	0.49	-0.593	-7.962	27.24	-7.22	21.2	18.34
**20_18**	1.26438	1.84284	-0.093	0.087	0.078	0.549	-0.601	-7.293	33.33	-10.49	18.13	23.88
**13_22**	1.30399	1.82052	-0.139	0.096	0.096	0.825	-0.595	-7.516	29.4	-4.26	25.65	13.8
**3_18**	1.27327	1.85208	-0.092	0.088	0.072	0.575	-0.599	-7.346	31.11	-7.84	10.64	20.84
**17_12**	1.19914	1.85714	0.232	0.035	0.059	0.496	-0.6	-7.733	14.77	-13.38	5.92	25.18
**1_18**	1.27846	1.80795	-0.113	0.095	0.095	0.369	-0.597	-7.421	32.51	-14.1	16.82	26.92
**19_1**	1.2	1.88019	-0.33	0.078	0.056	0.52	-0.588	-8.107	37.4	-11.81	32.88	24.29
**8_4**	1.29615	1.75724	0.315	0.043	0.17	0.561	-0.516	-8.742	23.08	9.76	34	14.15

**10_20**	1.26065	1.85709	-0.078	0.083	0.071	0.514	-0.616	-6.862	27.49	-12.32	10.91	23.57
**12_16**	1.29694	1.84053	-0.118	0.094	0.077	0.825	-0.643	-7.204	32.01	-1.57	21.23	12.32
**7_15**	1.29013	1.87137	0.061	0.059	0.102	0.421	-0.564	-8.06	7.45	-4.17	-0.64	19.13
**4_7**	1.37006	1.80261	-0.284	0.106	0.155	0.91	-0.579	-9.177	43.08	4.08	55.72	29.29
**12_7**	1.36511	1.83185	-0.257	0.098	0.123	0.82	-0.624	-8.724	32.77	-0.58	42.89	19.38
**4_2**	1.2977	1.63202	-0.156	0.087	0.071	0.894	-0.603	-8.04	33.27	-2.8	30.09	13.85
**9_2**	1.27855	1.85552	-0.099	0.11	0.119	0.43	-0.573	-8.163	28.25	-7.83	23.25	21.6
**9_9**	1.35987	1.84638	-0.211	0.136	0.216	0.473	-0.523	-9.117	36.72	2.27	39.53	31.27
**13_1**	1.25261	1.79253	-0.626	0.086	0.062	0.798	-0.36	-8.102	38.63	-5.41	34.04	15.98
**8_15**	1.32023	1.79866	-0.537	0.093	0.127	0.611	0.036	-8.378	7.26	0.7	0.6	15.82
**4_9**	1.37984	1.80452	-0.567	0.124	0.191	0.925	-0.27	-9.04	35.62	5.34	43.76	28.68
**12_1**	1.32138	1.70549	-0.647	0.083	0.046	0.797	-0.359	-8.06	38.31	-4.25	43.75	12.39
**14_1**	1.25049	1.79308	-0.626	0.087	0.064	0.798	-0.36	-8.084	38.79	-5.72	35.1	16.51

Table S4. Total potential (E), and Gibbs free energies (G) of all structures optimized at the SMD/M06-2X/6-31G(d) level of theory along with the total potential energies calculated by SMD/M06-2X/def2-TZVP//SMD/M06-2X/6-31G(d) and Cartesian coordinates for all of the calculated structures, as well as eigenvalues of imaginary frequencies of transition states.

1

E (SMD/M06-2X/6-31G(d)) = -607.442888426

G (SMD/M06-2X/6-31G(d)) = -607.321304

E (SMD/M06-2X/def2-TZVP//SMD/M06-2X/6-31G(d)) = -607.683031508

```
C    1.62224000  -0.54892200  -0.00001300
C    0.49784100   0.38992300   0.00000700
O    1.52096400  -1.75721500  -0.00003800
N    0.84704100   1.65945900   0.00002200
N    1.13062900   2.74872800   0.00003000
O    2.80083700   0.09614300   0.00000600
C    3.95706100  -0.74753000  -0.00000800
H    4.81133600  -0.07217500   0.00003700
H    3.96941200  -1.37695400   0.89232500
H    3.96944600  -1.37687700  -0.89239500
C   -0.94165200   0.07349300   0.00000300
C   -1.89240700   1.10675000  -0.00003300
C   -3.25193900   0.81919600  -0.00003500
C   -3.69478900  -0.50203600  -0.00000300
C   -2.75639100  -1.53045200   0.00003400
C   -1.39156500  -1.25521800   0.00003700
H   -1.57619600   2.14633900  -0.00006100
H   -3.96679700   1.63662300  -0.00006400
H   -4.75701100  -0.72539600  -0.00000600
```

H	-3.08394200	-2.56593600	0.00006000
H	-0.67474400	-2.06559700	0.00006600

$B(C_6F_5)_3$
E (SMD/M06-2X/6-31G(d)) = -2207.51326663
G (SMD/M06-2X/6-31G(d)) = -2207.414689
E (SMD/M06-2X/def2-TZVP//SMD/M06-2X/6-31G(d)) = -2208.4824978

B	0.00118300	0.00121600	0.00029800
C	-0.26993800	-1.54447700	0.00069700
C	-1.38155200	-2.10806800	0.63342100
C	0.58197700	-2.45394300	-0.63242200
C	-1.63292300	-3.47010500	0.65181600
C	0.35384700	-3.82011800	-0.64910900
C	-0.75963500	-4.33025800	0.00226100
C	-1.20232000	1.00790700	-0.00222600
C	-1.13922600	2.24949800	0.63650200
C	-2.41279100	0.72453600	-0.64083000
C	-2.19537500	3.14557800	0.65537300
C	-3.48393000	1.60256900	-0.65704400
C	-3.37371800	2.81928900	0.00006400
C	1.47485200	0.54030300	0.00202000
C	1.83577500	1.73129300	-0.63433300
C	2.51846500	-0.13805300	0.63778800
C	3.13337700	2.21572700	-0.65463400
C	3.82407500	0.32435200	0.65271800
C	4.13224300	1.50777000	-0.00237100
F	1.67175900	-2.02683100	-1.27539200
F	1.19094300	-4.64295800	-1.27725300
F	-0.98884700	-5.63578500	0.00371100
F	-2.70063400	-3.95733900	1.28031200
F	-2.26083100	-1.33371900	1.27439800
F	2.28658100	-1.28347700	1.28411600
F	4.78035700	-0.35456100	1.28284100
F	5.37773300	1.96131300	-0.00492800
F	3.42771000	3.34933500	-1.28758500
F	0.91983600	2.46060700	-1.27678300
F	-0.03191600	2.62288500	1.28305300
F	-2.08934500	4.31097600	1.29016400
F	-4.39190600	3.66789100	0.00226200
F	-4.61231800	1.28960900	-1.29045800
F	-2.58191400	-0.43115500	-1.28842000

BCl_3
E (SMD/M06-2X/6-31G(d)) = -1405.46167567
G (SMD/M06-2X/6-31G(d)) = -1405.483213
E (SMD/M06-2X/def2-TZVP//SMD/M06-2X/6-31G(d)) = -1405.57543303

B	0.00010300	-0.00011200	-0.00002500

Cl	-1.26595600	-1.20083000	0.00000200
Cl	-0.40721000	1.69654700	0.00000200
Cl	1.67313600	-0.49568400	0.00000200

N_2

E (SMD/M06-2X/6-31G(d)) = -109.482263966

G (SMD/M06-2X/6-31G(d)) = -109.494897

E (SMD/M06-2X/def2-TZVP//SMD/M06-2X/6-31G(d)) = -109.530284564

N	0.00000000	0.00000000	0.54917600
N	0.00000000	0.00000000	-0.54917600

2

E (SMD/M06-2X/6-31G(d)) = -2814.99425646

G (SMD/M06-2X/6-31G(d)) = -2814.748128

E (SMD/M06-2X/def2-TZVP//SMD/M06-2X/6-31G(d)) = -2816.19576608

C	0.82216500	-0.92932800	-2.33017900
C	2.20751000	-0.60752100	-2.26752600
O	-0.09399400	-0.38193700	-1.66146200
B	-0.41076500	-0.07033200	-0.15696600
C	-1.70916600	-1.02065500	0.15376400
C	-1.87647900	-1.85815900	1.25024900
C	-2.80122500	-0.96392500	-0.71082900
C	-3.02409800	-2.61372300	1.46385300
C	-3.95957800	-1.70658100	-0.54110900
C	-4.07138300	-2.54045900	0.56135400
C	-0.93609900	1.46383600	-0.05460800
C	-1.16387100	2.34499500	-1.10288700
C	-1.35864400	1.91616500	1.19267800
C	-1.71280600	3.61125200	-0.92600500
C	-1.91165100	3.16521500	1.41561600
C	-2.09149800	4.02337400	0.33944400
C	0.90801500	-0.44631100	0.71558200
C	1.41603200	-1.73815000	0.61269600
C	1.64275200	0.40304500	1.53554100
C	2.56151900	-2.17948900	1.25215700
C	2.79425800	0.00235300	2.20393800
C	3.25730800	-1.29389100	2.06282100
F	-2.76687700	-0.15123500	-1.78165600
F	-4.96642800	-1.61506500	-1.41257600
F	-5.17614200	-3.25916500	0.75112500
F	-3.12433400	-3.40610300	2.53313600
F	-0.91930200	-1.98288300	2.18145500
F	1.29877300	1.68591500	1.70801300
F	3.47997500	0.87247100	2.94725700
F	4.37129600	-1.68214700	2.68002500
F	2.99919300	-3.43070000	1.09769500
F	0.77876400	-2.63278400	-0.17273500

F	-0.85152800	2.02194600	-2.37003300
F	-1.89369200	4.42160300	-1.97157600
F	-2.62413900	5.23023100	0.52411300
F	-2.28185000	3.54584500	2.63994500
F	-1.22194500	1.10818700	2.25751300
O	0.50687900	-1.84214100	-3.21795200
C	-0.85045600	-2.33793400	-3.21114400
C	2.86906300	0.48486100	-1.52428400
C	4.09252300	0.26343900	-0.88582800
C	2.25238700	1.73722600	-1.44452700
C	4.68107600	1.28644000	-0.14766900
H	4.57455500	-0.70939400	-0.94805400
C	2.83531500	2.74528700	-0.68446900
H	1.32122900	1.91293300	-1.97231400
C	4.04607500	2.52172600	-0.03001300
H	5.62596500	1.10781000	0.35674100
H	2.33971400	3.70811900	-0.60510300
H	4.49665300	3.30945600	0.56556800
H	-0.88622700	-3.06002100	-4.02383000
H	-1.05332200	-2.82226300	-2.25398900
H	-1.54553600	-1.51708300	-3.38499000
N	2.98076000	-1.42674000	-2.98311000
N	3.65460800	-2.10452500	-3.56130500

3
E (SMD/M06-2X/6-31G(d)) = -2705.47897604
G (SMD/M06-2X/6-31G(d)) = -2705.242907
E (SMD/M06-2X/def2-TZVP//SMD/M06-2X/6-31G(d)) = -2706.63800818

C	-0.93890200	0.44035000	2.15542700
C	-1.94260700	1.05668000	1.38921300
O	0.11897600	-0.12065100	1.70967500
B	0.51944100	-0.10265000	0.22543700
C	1.68482300	-1.23612600	0.12277400
C	1.77064500	-2.23281500	-0.84028200
C	2.73900500	-1.19931900	1.03142200
C	2.81129400	-3.15195400	-0.89341900
C	3.79466400	-2.09895800	1.01729300
C	3.82904100	-3.08585300	0.04395600
C	1.20204800	1.33431900	-0.13081000
C	1.41243600	2.40024800	0.73101500
C	1.72888800	1.49751800	-1.40811600
C	2.09332400	3.55512000	0.35843900
C	2.41071000	2.62714400	-1.82471100
C	2.59799600	3.66814300	-0.92493500
C	-0.84449300	-0.43130200	-0.60807700
C	-1.52194200	-1.61423100	-0.30326800
C	-1.50962400	0.41545400	-1.49408500

C	-2.78995800	-1.91521700	-0.76233400
C	-2.78011100	0.14617200	-1.98820000
C	-3.43430900	-1.01022400	-1.59922200
F	2.77655200	-0.24684200	1.97855500
F	4.77613900	-2.01911200	1.91901300
F	4.83370100	-3.96001800	0.00854600
F	2.83920000	-4.09477400	-1.83824200
F	0.82727800	-2.35614200	-1.78652900
F	-0.99106900	1.59203300	-1.85977200
F	-3.40796500	1.03072100	-2.76379500
F	-4.68100100	-1.24135100	-1.99233500
F	-3.42634700	-3.01754600	-0.36730700
F	-0.96758800	-2.48707200	0.55055600
F	0.95236100	2.38234300	1.99670400
F	2.26419800	4.55191100	1.23026100
F	3.25257200	4.76720400	-1.29760700
F	2.88634100	2.72821000	-3.06769200
F	1.55042800	0.51666200	-2.31032300
O	-1.03980800	0.62185800	3.45222400
C	0.13607300	0.35388300	4.25190600
C	-3.28510000	0.71111400	1.23535300
C	-3.83983600	-0.49926300	1.75002500
C	-4.12195900	1.58114200	0.47794900
C	-5.16783500	-0.80775600	1.52898100
H	-3.19852700	-1.17752200	2.30827500
C	-5.44632700	1.25891300	0.24991900
H	-3.68289100	2.48891800	0.07398400
C	-5.96201700	0.06841400	0.77658100
H	-5.59156000	-1.72717300	1.91783600
H	-6.08158900	1.91493700	-0.33480000
H	-7.00204400	-0.18575700	0.59340800
H	-0.12816400	0.68915800	5.25263700
H	0.34810100	-0.71609400	4.24603200
H	0.98734600	0.91150500	3.86321300

indole
E (SMD/M06-2X/6-31G(d)) = -363.677611641
G (SMD/M06-2X/6-31G(d)) = -363.576284
E (SMD/M06-2X/def2-TZVP//SMD/M06-2X/6-31G(d)) = -363.815667193

C	-0.24975700	0.75153700	0.00017200
C	-0.25020400	-0.66737300	0.00004000
C	0.93231500	-1.41772300	0.00027200
C	2.12879000	-0.72106000	0.00000800
C	2.15506900	0.69011600	-0.00037700
C	0.98381500	1.42771200	0.00003400
C	-1.62492200	1.16662400	0.00026400

C	-2.38173900	0.02663900	-0.00007300
H	0.90661400	-2.50345600	0.00088300
H	3.06541900	-1.27053600	0.00062700
H	3.11307900	1.20141000	-0.00067300
H	1.01292800	2.51399400	-0.00008900
H	-1.99935400	2.18072800	0.00069200
H	-3.45567700	-0.09716200	-0.00014900
N	-1.56105400	-1.07537600	-0.00045700
H	-1.87582600	-2.03617100	-0.00012300

TS^{i}

E (SMD/M06-2X/6-31G(d)) = -3069.16520106

G (SMD/M06-2X/6-31G(d)) = -3068.805492

E (SMD/M06-2X/def2-TZVP//SMD/M06-2X/6-31G(d)) = -3070.45502707

C	1.31889200	0.21015000	-1.07630300
C	1.66873200	1.10085200	-0.05897100
O	0.15328200	-0.32641400	-1.17586700
B	-0.97699600	-0.23492600	-0.14393300
C	-2.15013600	-1.19267900	-0.76304100
C	-3.50078800	-0.88159900	-0.86513800
C	-1.81596400	-2.48339000	-1.16670200
C	-4.45173700	-1.76465200	-1.36259200
C	-2.73102800	-3.39469100	-1.67260900
C	-4.06457700	-3.02959200	-1.77178400
C	-1.38182900	1.34502700	-0.04027500
C	-1.18444900	2.18741700	1.04660700
C	-1.84215600	1.98290700	-1.19120100
C	-1.40948100	3.55898700	1.00373700
C	-2.08381900	3.34370300	-1.28030700
C	-1.85621500	4.14200700	-0.16831000
C	-0.52785100	-0.93601600	1.26337100
C	0.60741600	-1.70380800	1.48590400
C	-1.42725500	-0.90681100	2.32494900
C	0.86512300	-2.35260600	2.68870500
C	-1.21643200	-1.54002400	3.53798800
C	-0.05190100	-2.27244900	3.72196200
F	-0.54742300	-2.91488000	-1.05824200
F	-2.34484700	-4.61656800	-2.04906200
F	-4.96437100	-3.88811200	-2.24997700
F	-5.73492500	-1.40424100	-1.44656700
F	-3.96605800	0.31813500	-0.48480700
F	-2.57419500	-0.21929700	2.18773300
F	-2.11645800	-1.46228200	4.52119500
F	0.17747300	-2.89317800	4.87854900
F	1.98765100	-3.06168500	2.84989200
F	1.54332500	-1.86593000	0.53337200
F	-0.66892600	1.73200000	2.19891000

F	-1.15034300	4.32266500	2.06833800
F	-2.02992100	5.45996300	-0.23969900
F	-2.50879800	3.89537600	-2.41951600
F	-2.06324200	1.26367400	-2.30280300
O	2.25401800	-0.22542400	-1.88442200
C	2.02480700	-1.48717400	-2.54769900
C	1.68959900	2.50425400	0.00491300
C	1.93841000	3.12080700	1.25999500
C	1.40306200	3.32115700	-1.12241100
C	1.89374700	4.49768600	1.38224700
H	2.14460700	2.48542100	2.11589900
C	1.35137100	4.69768100	-0.98868600
H	1.21906100	2.84663800	-2.08277100
C	1.59471300	5.28027300	0.26057200
H	2.07290000	4.97117400	2.34155800
H	1.11678100	5.32352300	-1.84361500
H	1.54751500	6.36056800	0.36199500
C	4.58923800	-0.99278600	-0.16425800
C	5.19427300	-0.70476200	-1.41270400
C	5.67793200	-1.70461800	-2.26251900
C	5.53824400	-3.01750100	-1.83961300
C	4.93201800	-3.32968200	-0.60513600
C	4.45916600	-2.33420900	0.23439000
C	4.21712200	0.26774400	0.41760400
C	4.60031000	1.24270800	-0.48053100
H	6.14209900	-1.45899000	-3.21305800
H	5.90220900	-3.82202400	-2.47165300
H	4.83620100	-4.37048200	-0.31115200
H	3.97940100	-2.58247400	1.17605600
H	3.80059400	0.43085700	1.40060000
H	4.51781300	2.31870100	-0.41392400
N	5.18492800	0.66294800	-1.56899200
H	5.52470100	1.16195500	-2.38057500
H	1.19482600	-1.39775300	-3.25019300
H	2.95479500	-1.69388900	-3.07403400
H	1.81675400	-2.26114100	-1.80901500

benzofuran
E (SMD/M06-2X/6-31G(d)) = -383.524626529
G (SMD/M06-2X/6-31G(d)) = -383.435656
E (SMD/M06-2X/def2-TZVP//SMD/M06-2X/6-31G(d)) = -383.671127045

C	0.25935100	0.75421900	0.00012900
C	0.24939500	-0.64818000	0.00022800
C	-0.91019300	-1.41401200	0.00004200
C	-2.11013800	-0.71338800	0.00009800
C	-2.13593400	0.69336200	-0.00004700
C	-0.96452900	1.43796900	-0.00031300

C	1.65656400	1.12560200	0.00013200
C	2.34528700	-0.03646500	0.00014800
H	-0.87128300	-2.49811700	0.00021100
H	-3.04610500	-1.26320400	0.00005900
H	-3.09407100	1.20384100	-0.00016000
H	-0.99379300	2.52337200	0.00019400
H	2.07664900	2.12118900	0.00016300
H	3.40320600	-0.25684400	-0.00033000
O	1.52332200	-1.12811100	-0.00033100

TS[ii]
E (SMD/M06-2X/6-31G(d)) = -3089.01078985
G (SMD/M06-2X/6-31G(d)) = -3088.663417
E (SMD/M06-2X/def2-TZVP//SMD/M06-2X/6-31G(d)) = -3090.30861851

C	1.32625400	0.33635600	-1.16394900
C	1.63798200	1.26034600	-0.17051300
O	0.19455500	-0.27705300	-1.22216800
B	-0.90727700	-0.31317700	-0.15945500
C	-1.97760000	-1.41079600	-0.73559900
C	-3.36021200	-1.27072800	-0.76801200
C	-1.50750800	-2.65195500	-1.15898000
C	-4.21828100	-2.26526200	-1.22203100
C	-2.32699900	-3.67012800	-1.62375800
C	-3.69854600	-3.47284100	-1.65670600
C	-1.50603500	1.20409700	-0.05076500
C	-1.39120700	2.07185800	1.02792100
C	-2.09086100	1.76496900	-1.18434900
C	-1.81556600	3.39534800	0.99539900
C	-2.53032300	3.07666900	-1.26329900
C	-2.38433500	3.90299600	-0.15904400
C	-0.34657700	-0.95051300	1.23834600
C	0.86214400	-1.60670800	1.42991800
C	-1.21560800	-1.00271200	2.32448200
C	1.21510200	-2.21737900	2.62887200
C	-0.90992600	-1.60172100	3.53455500
C	0.32570400	-2.21466400	3.68883500
F	-0.19232700	-2.92601000	-1.10951100
F	-1.81215000	-4.83601600	-2.02304100
F	-4.50748000	-4.43679600	-2.09492000
F	-5.53877200	-2.06636500	-1.23990400
F	-3.95234000	-0.13969800	-0.35510700
F	-2.43077100	-0.43914800	2.21341200
F	-1.78475000	-1.60464700	4.54296400
F	0.64743900	-2.79934700	4.84224000
F	2.40458000	-2.81438700	2.76223300
F	1.77886500	-1.69191000	0.44866700
F	-0.78092400	1.69667200	2.16341500

F	-1.63488600	4.19286800	2.05167200
F	-2.75345700	5.18092200	-0.21991500
F	-3.07099600	3.55529200	-2.38665400
F	-2.25090300	1.01583400	-2.28700500
O	2.25771100	-0.04484300	-2.00763400
C	2.10328700	-1.32810200	-2.64917400
C	1.43410900	2.64924800	-0.03918100
C	1.62230500	3.25326000	1.23005900
C	0.99225700	3.44789500	-1.12667000
C	1.36468400	4.60137000	1.40663100
H	1.95177000	2.63196500	2.05737000
C	0.72558500	4.79319000	-0.93843600
H	0.85972300	2.98311700	-2.10019200
C	0.91200900	5.36512900	0.32522500
H	1.49697600	5.06294700	2.37928800
H	0.36965100	5.40195700	-1.76340000
H	0.69886600	6.42037600	0.46912700
C	4.63425700	-0.39195900	-0.17279100
C	5.09070700	-0.20903800	-1.48390700
C	5.66590600	-1.21179900	-2.25202000
C	5.77086600	-2.45924100	-1.64654000
C	5.31477400	-2.67957200	-0.33416300
C	4.74428200	-1.65898700	0.41438800
C	4.11523700	0.89415200	0.23529000
C	4.29666600	1.71732700	-0.84193300
H	6.00801700	-1.02584700	-3.26444700
H	6.21344000	-3.28143600	-2.20006000
H	5.40936800	-3.67072500	0.09797400
H	4.37605400	-1.84012100	1.41853100
H	3.78478900	1.18368900	1.22104300
H	4.09666400	2.76886700	-0.99265700
H	1.16182100	-1.37046700	-3.19674300
H	2.94707000	-1.39592600	-3.33329400
H	2.14757200	-2.11965000	-1.90007700
O	4.88239000	1.08078500	-1.88278800

indene
E (SMD/M06-2X/6-31G(d)) = -347.618649971
G (SMD/M06-2X/6-31G(d)) = -347.506941
E (SMD/M06-2X/def2-TZVP//SMD/M06-2X/6-31G(d)) = -347.746252541

C	0.21122400	0.72037100	-0.00014800
C	0.22996000	-0.68741300	-0.00028800
C	-0.95535200	-1.40875200	-0.00017400
C	-2.16755900	-0.71212700	0.00004000
C	-2.18650700	0.68450300	0.00012300
C	-0.99722400	1.41440700	0.00005400
C	1.60151700	1.19582900	-0.00012900

C	2.43387400	0.14129600	0.00001900
H	-0.94625200	-2.49578700	-0.00014300
H	-3.10390100	-1.26229200	0.00007100
H	-3.13828700	1.20780100	0.00027800
H	-1.01496300	2.50092900	0.00013900
H	1.88702300	2.24241000	-0.00003400
H	3.51733900	0.18455400	0.00014200
C	1.66368400	-1.15475500	0.00016400
H	1.89921100	-1.76952100	-0.87841800
H	1.89812100	-1.76824500	0.87999600

TS[iii]
E (SMD/M06-2X/6-31G(d)) = -3053.10501529
G (SMD/M06-2X/6-31G(d)) = -3052.734262
E (SMD/M06-2X/def2-TZVP//SMD/M06-2X/6-31G(d)) = -3054.38389012

C	1.31946900	0.41850300	-1.16747200
C	1.57897600	1.36084700	-0.18142900
O	0.22594700	-0.26384000	-1.21732000
B	-0.88278700	-0.34680800	-0.16672500
C	-1.88668600	-1.50616400	-0.74342900
C	-3.27417800	-1.44023200	-0.79611300
C	-1.34559700	-2.72582900	-1.14386600
C	-4.07215400	-2.48577100	-1.24555800
C	-2.10361800	-3.79286700	-1.60317200
C	-3.48321000	-3.66993800	-1.65543900
C	-1.56698200	1.13566700	-0.08159900
C	-1.53750900	2.01131900	0.99658100
C	-2.15149200	1.65621300	-1.23426000
C	-2.04346700	3.30517700	0.94618400
C	-2.66937100	2.93771200	-1.33159700
C	-2.60880200	3.77344200	-0.22653500
C	-0.30903400	-0.94035600	1.24516800
C	0.93183800	-1.52607400	1.45841100
C	-1.19100700	-1.03940100	2.31773000
C	1.30011600	-2.11089000	2.66578400
C	-0.87036600	-1.61443700	3.53557300
C	0.39584700	-2.15476600	3.71201600
F	-0.01854500	-2.92863500	-1.07487600
F	-1.52214700	-4.93505600	-1.97897300
F	-4.23363100	-4.68242600	-2.08849200
F	-5.40110500	-2.35834300	-1.28210300
F	-3.93150700	-0.33695200	-0.40686700
F	-2.43629700	-0.55122700	2.18483900
F	-1.75972800	-1.66396100	4.53001900
F	0.73274500	-2.71557300	4.87294400
F	2.51838600	-2.64123700	2.81981700
F	1.86530300	-1.56612400	0.49085800

F	-0.93979900	1.67714400	2.15130100
F	-1.94266400	4.11625600	2.00278100
F	-3.05478000	5.02585300	-0.30361800
F	-3.20524600	3.37949500	-2.47224000
F	-2.23263000	0.89486700	-2.33730500
O	2.26588400	0.08807700	-2.01735900
C	2.19630100	-1.21701800	-2.62746800
C	1.28433200	2.72673900	-0.01408100
C	1.42957200	3.30698400	1.27160600
C	0.80379600	3.52506500	-1.08526900
C	1.09308200	4.63307100	1.47967200
H	1.78837500	2.68386900	2.08504800
C	0.46247400	4.84832700	-0.86643500
H	0.70414000	3.07659800	-2.07028800
C	0.60722300	5.39734400	0.41305300
H	1.19129100	5.07663300	2.46464000
H	0.07900900	5.45733100	-1.67882200
H	0.33407300	6.43502300	0.58118800
C	4.68940100	-0.22029900	-0.20564500
C	5.17927500	-0.01621100	-1.50893300
C	5.78495700	-1.05352700	-2.20414600
C	5.89569000	-2.30095600	-1.58358000
C	5.40601500	-2.50391100	-0.29010400
C	4.79692200	-1.46509800	0.41258700
C	4.10763700	1.03967600	0.26810700
C	4.21156900	1.97296500	-0.71398300
H	6.16572100	-0.90365300	-3.21113200
H	6.36795900	-3.12362400	-2.11239100
H	5.49908200	-3.48268300	0.17085000
H	4.39951200	-1.62765900	1.40949900
H	3.74150200	1.19954200	1.27531700
H	3.91791000	3.01293800	-0.63507700
H	1.26944600	-1.32645100	-3.19097500
H	3.05691400	-1.25369400	-3.29323400
H	2.27091500	-1.98639600	-1.85786400
C	4.90945500	1.40800700	-1.92032000
H	4.29158300	1.47148500	-2.82421100
H	5.83381800	1.96427000	-2.12648900

styrene
E (SMD/M06-2X/6-31G(d)) = -309.511613917
G (SMD/M06-2X/6-31G(d)) = -309.407898
E (SMD/M06-2X/def2-TZVP//SMD/M06-2X/6-31G(d)) = -309.628707908

C	0.51143800	-0.22428100	-0.02164200
C	0.01557900	1.08809900	-0.03075200
C	-1.35308600	1.32907100	-0.00999000
C	-2.25680200	0.26545300	0.01783200

C	-1.77841000	-1.04225100	0.02232400
C	-0.40671400	-1.28186600	0.00101500
C	1.95403300	-0.53531600	-0.03608100
C	2.95856500	0.33919100	0.04830800
H	-1.71854400	2.35177800	-0.01866100
H	-3.32538700	0.45770300	0.03279500
H	-2.47214500	-1.87763000	0.04137500
H	-0.03557100	-2.30391200	0.00432900
H	2.19000500	-1.59593800	-0.11724300
H	3.99010300	0.00154300	0.02856500
H	2.79974200	1.41018200	0.14304300
H	0.70418000	1.92768000	-0.06029200

TS[iv]
E (SMD/M06-2X/6-31G(d)) = -3014.99754852
G (SMD/M06-2X/6-31G(d)) = -3014.634741
E (SMD/M06-2X/def2-TZVP//SMD/M06-2X/6-31G(d)) = -3016.26605428

C	0.95313500	0.94988400	-1.43417700
C	0.91198600	1.97645600	-0.49065900
O	0.24964500	-0.12283400	-1.32307900
B	-0.60722600	-0.59805100	-0.14320600
C	-1.09488700	-2.09890700	-0.58604400
C	-2.37964900	-2.61432600	-0.45643200
C	-0.14954000	-3.00698500	-1.05762800
C	-2.72022400	-3.91574300	-0.80588500
C	-0.44639700	-4.31170700	-1.42351600
C	-1.74810200	-4.77045100	-1.29723100
C	-1.83601500	0.46968900	0.01152900
C	-2.05626000	1.34737000	1.06594300
C	-2.71899700	0.61606600	-1.05620200
C	-3.07201600	2.29640000	1.07339100
C	-3.74543500	1.54617200	-1.09384200
C	-3.92000900	2.39962000	-0.01493000
C	0.31671100	-0.81604000	1.18601200
C	1.70485600	-0.84485300	1.22730700
C	-0.31023200	-1.17776900	2.37502000
C	2.42516400	-1.13488300	2.38160000
C	0.36467900	-1.48034000	3.54527600
C	1.75233100	-1.45275100	3.54822800
F	1.14077900	-2.64451600	-1.15801600
F	0.50710800	-5.12941500	-1.87730900
F	-2.05836900	-6.02063600	-1.63803900
F	-3.97630300	-4.34833300	-0.66956300
F	-3.38388300	-1.86308600	0.02093600
F	-1.65224300	-1.24324500	2.41193000
F	-0.29711300	-1.80567400	4.65831500
F	2.42840000	-1.73791700	4.66065700

F	3.76202300	-1.11759700	2.37166100
F	2.44221300	-0.59415900	0.13285300
F	-1.23833300	1.37946400	2.13000600
F	-3.20506400	3.14125800	2.09934900
F	-4.86808500	3.33421000	-0.04100200
F	-4.55054900	1.64010700	-2.15510200
F	-2.59669000	-0.17870000	-2.13146700
O	1.83683400	0.98331500	-2.40548500
C	2.16317700	-0.26852100	-3.04580500
C	0.07003700	3.08883900	-0.31837600
C	0.10181300	3.77478500	0.92309400
C	-0.83014300	3.52238700	-1.32868100
C	-0.74555100	4.84463500	1.15066800
H	0.79101300	3.42819600	1.68681900
C	-1.67372500	4.59279900	-1.09283400
H	-0.84432000	2.99798900	-2.28064800
C	-1.63055100	5.24758000	0.14464400
H	-0.73390100	5.36339900	2.10305200
H	-2.37197100	4.92056700	-1.85615400
H	-2.30050800	6.08270900	0.32760900
C	4.41235700	1.78974800	-0.83223300
C	4.88805000	1.44814400	-2.10763500
C	5.74607700	0.36824200	-2.27543400
C	6.14758500	-0.38807600	-1.17215400
C	5.68781600	-0.05461600	0.09919700
C	4.82989600	1.02900500	0.26612500
C	3.49989100	2.92111400	-0.60875900
C	2.84380400	3.61376100	-1.55724500
H	6.10831300	0.11683600	-3.26803800
H	6.81949700	-1.23061500	-1.30571900
H	5.99129400	-0.64049300	0.96107100
H	4.46046700	1.28101800	1.25722200
H	3.37628100	3.21749000	0.43221500
H	2.23804500	4.47369200	-1.29168800
H	1.27600400	-0.69819600	-3.51228500
H	2.90260800	-0.00659200	-3.80025600
H	2.58331100	-0.95892800	-2.31418700
H	2.91988600	3.37563500	-2.61377900
H	4.59346700	2.03748700	-2.97156200

$TS_{3\text{-}4}$

E (SMD/M06-2X/6-31G(d)) = -2705.44055559

G (SMD/M06-2X/6-31G(d)) = -2705.205541

E (SMD/M06-2X/def2-TZVP//SMD/M06-2X/6-31G(d)) = -2706.60131696

C	-1.00129500	0.36569400	2.02635900
C	-2.27355400	0.49641500	1.95058700
O	0.18323600	0.03150200	1.82195300

B	0.62409200	-0.13008500	0.29610500
C	1.83291000	-1.21280400	0.32801800
C	1.98699000	-2.28208800	-0.54626900
C	2.87930000	-1.03080800	1.22847600
C	3.08188600	-3.13681800	-0.51474100
C	3.98694900	-1.86244300	1.29679000
C	4.08705400	-2.92756400	0.41497800
C	1.25571300	1.28457300	-0.19735600
C	1.62990300	2.35391600	0.60580600
C	1.59870300	1.40886100	-1.54090700
C	2.26761400	3.48700900	0.10835300
C	2.23304100	2.51518400	-2.07779700
C	2.57241600	3.56811300	-1.23867000
C	-0.73758800	-0.60727000	-0.47240300
C	-1.29434300	-1.84885000	-0.16278600
C	-1.52987000	0.19019700	-1.29147500
C	-2.52671900	-2.28319000	-0.62540000
C	-2.76515000	-0.21039900	-1.78983400
C	-3.27331300	-1.45036700	-1.44661000
F	2.85247000	0.00773700	2.07809600
F	4.95641600	-1.64081800	2.18693700
F	5.14274000	-3.73813000	0.45794500
F	3.17382300	-4.15450800	-1.37360000
F	1.06555500	-2.54523200	-1.48411500
F	-1.16698200	1.44373800	-1.60471200
F	-3.49207000	0.62317200	-2.53621600
F	-4.47318600	-1.83087600	-1.87737300
F	-3.01241800	-3.47260300	-0.26766600
F	-0.63475900	-2.68286800	0.65571500
F	1.38986900	2.35890900	1.92769100
F	2.59472600	4.49109500	0.92541900
F	3.18485800	4.64501400	-1.72754400
F	2.52395100	2.57998700	-3.37835000
F	1.29111000	0.40811400	-2.38324000
O	-1.56029400	1.37408800	3.06632600
C	-1.41803200	2.78545800	2.72905500
C	-3.62489100	0.45896700	1.45716200
C	-4.44106400	-0.63885100	1.76230500
C	-4.05973000	1.44755200	0.56114100
C	-5.68834400	-0.74978000	1.15843800
H	-4.08060300	-1.40131600	2.44573400
C	-5.31570700	1.33628400	-0.02205000
H	-3.39758600	2.27043600	0.30502900
C	-6.12204300	0.23527300	0.27073500
H	-6.31931000	-1.60543100	1.37518200
H	-5.65554900	2.09135100	-0.72306700
H	-7.09399600	0.14035400	-0.20367900

H	-2.37612200	3.25606400	2.95181900
H	-0.62766600	3.17719400	3.36776500
H	-1.16312500	2.88887000	1.67211700

4

E (SMD/M06-2X/6-31G(d)) = -497.958646465

G (SMD/M06-2X/6-31G(d)) = -497.845344

E (SMD/M06-2X/def2-TZVP//SMD/M06-2X/6-31G(d)) = -498.156618097

C	1.91682000	1.13923300	-0.18762600
C	1.16155200	0.04420600	-0.23258600
O	2.58525100	2.09455600	-0.17275100
O	1.82296900	-1.15039800	-0.43964200
C	2.04076200	-1.87765900	0.77103400
C	-0.30230800	0.07608100	-0.11020800
C	-0.99288700	1.22760900	0.29396300
C	-1.02678900	-1.08312800	-0.41290500
C	-2.37978500	1.21949700	0.37779400
H	-0.44269200	2.13111000	0.54847500
C	-2.41585000	-1.08664400	-0.31348600
H	-0.49510700	-1.97437800	-0.73203600
C	-3.09950000	0.06193100	0.07804200
H	-2.90127000	2.11992500	0.68870700
H	-2.96495400	-1.99345900	-0.54986400
H	-4.18245300	0.05689100	0.15206100
H	2.47887700	-2.83456400	0.48154300
H	2.73248100	-1.33595300	1.42536500
H	1.09726000	-2.04959300	1.30077100

$TS_{3\text{-}5}$

E (SMD/M06-2X/6-31G(d)) = -2705.43734864

G (SMD/M06-2X/6-31G(d)) = -2705.202124

E (SMD/M06-2X/def2-TZVP//SMD/M06-2X/6-31G(d)) = -2706.59652496

C	1.31804600	-2.07097800	1.63686700
C	1.60618800	-0.66382100	1.87797500
O	1.07663000	-2.52852900	0.53142100
B	-0.73869200	0.33402200	-0.34698600
C	-1.56801700	-1.01917000	-0.38371900
C	-2.15448800	-1.33924600	-1.60176800
C	-1.80637200	-1.90392700	0.65733100
C	-2.92566300	-2.47083600	-1.80412200
C	-2.56710300	-3.05375900	0.49267200
C	-3.12986700	-3.33896800	-0.74087400
C	0.71290500	0.40613400	-0.91474600
C	1.56868700	1.48398800	-0.64308600
C	1.28453800	-0.62818100	-1.67063900
C	2.87950700	1.54580000	-1.08014300
C	2.60489800	-0.61364300	-2.09222500

C	3.40713500	0.47671700	-1.79047800
C	-1.52849000	1.62339700	0.07601300
C	-2.44612200	1.62816900	1.12726300
C	-1.45572200	2.80446100	-0.66705800
C	-3.21931400	2.73289600	1.44964800
C	-2.22673700	3.91991900	-0.38634300
C	-3.10920900	3.88387900	0.68427500
F	-1.29763300	-1.69326600	1.87554000
F	-2.74962900	-3.89041500	1.51668000
F	-3.86209500	-4.43692200	-0.90552900
F	-3.46210100	-2.73335000	-2.99547200
F	-1.94664300	-0.52141300	-2.64697600
F	-0.63830600	2.89033300	-1.72034800
F	-2.13112900	5.01994000	-1.13044500
F	-3.84801100	4.94725600	0.97121800
F	-4.06520900	2.69529700	2.47714300
F	-2.59924800	0.54477000	1.89063900
F	1.16648700	2.49637500	0.12734000
F	3.64923600	2.59230200	-0.78659500
F	4.67438700	0.49755600	-2.17465800
F	3.10485900	-1.62983200	-2.79269900
F	0.56892400	-1.68222400	-2.04496400
O	1.23243300	-2.77045500	2.77414300
C	0.73644100	-4.10740800	2.63400600
C	2.95069500	-0.23577300	1.71069600
C	3.26102500	1.10519200	2.05655900
C	3.97452000	-1.05024200	1.15580900
C	4.53933100	1.60481000	1.87738600
H	2.46572400	1.72293500	2.46304200
C	5.23980000	-0.53292700	0.93446000
H	3.74429100	-2.07422400	0.87224800
C	5.51831400	0.78836700	1.30198600
H	4.77599100	2.62787900	2.14953600
H	6.01474600	-1.14603800	0.48627800
H	6.51444900	1.18790500	1.13413200
H	0.75853500	-4.53397200	3.63591400
H	-0.28774400	-4.09161200	2.25417300
H	1.37038500	-4.68787800	1.96076700

5

E (SMD/M06-2X/6-31G(d)) = -2705.506052

G (SMD/M06-2X/6-31G(d)) = -2705.267876

E (SMD/M06-2X/def2-TZVP//SMD/M06-2X/6-31G(d)) = -2706.661422

C	1.30095900	0.72950000	2.27602100
O	0.67159400	1.53162600	2.91651300
C	0.66901000	-0.42037900	1.51442500
B	-0.06106800	-0.00812300	0.12129700

C	-0.82679200	1.43283200	0.22054900
C	-0.80214800	2.42408900	-0.75321600
C	-1.71377500	1.65975100	1.26830300
C	-1.56651200	3.58199600	-0.67867000
C	-2.49298000	2.80002100	1.38356800
C	-2.41536400	3.77325900	0.39872200
C	-1.19997300	-1.01259100	-0.47866300
C	-1.47523900	-1.01682000	-1.84406900
C	-2.08998700	-1.75094500	0.29454800
C	-2.52652200	-1.71587900	-2.41654200
C	-3.15534400	-2.46797000	-0.23667000
C	-3.37761200	-2.44855600	-1.60305600
C	1.36177200	0.03909700	-0.73088100
C	1.82933000	-1.02074700	-1.50790100
C	2.21745300	1.14000900	-0.68157200
C	3.02929300	-0.98952200	-2.20334200
C	3.42735900	1.20978600	-1.35214300
C	3.83769700	0.13331700	-2.12282000
F	-1.85452200	0.72955900	2.22466900
F	-3.31897900	2.96511200	2.41946000
F	-3.15660500	4.87700700	0.48438600
F	-1.49253500	4.50705800	-1.63897800
F	-0.02084500	2.30494400	-1.83720900
F	1.88943900	2.21552400	0.05168600
F	4.19617500	2.29470600	-1.25644300
F	4.99428300	0.17572700	-2.77558100
F	3.41037900	-2.03017300	-2.94446500
F	1.11229400	-2.14698200	-1.63895800
F	-0.69961000	-0.31157400	-2.68153600
F	-2.72986600	-1.68518600	-3.73492900
F	-4.39480900	-3.12671500	-2.13090100
F	-3.96478400	-3.16940600	0.55958100
F	-1.93648800	-1.82935600	1.62590300
O	2.62270500	0.71227200	2.14500700
C	3.31319700	1.83137800	2.72879700
H	3.23424300	1.79107600	3.81711900
H	4.35012600	1.72695700	2.41477200
H	2.88884300	2.76543700	2.35774900
C	0.95548300	-1.69575600	2.06373200
C	0.66010200	-2.89360200	1.35452700
C	1.54162200	-1.80339000	3.35912700
C	0.95370800	-4.12406000	1.90455600
H	0.24520900	-2.83604900	0.35636300
C	1.78919500	-3.04055100	3.91913400
H	1.76431400	-0.91049100	3.93490200
C	1.50471800	-4.19839900	3.18894800
H	0.74841800	-5.03143600	1.34751600

H	2.21028200	-3.11299800	4.91554500
H	1.71587100	-5.17046400	3.62473300

$TS_{3\text{-}6}$

E (SMD/M06-2X/6-31G(d)) = -2705.47849494

G (SMD/M06-2X/6-31G(d)) = -2705.242121

E (SMD/M06-2X/def2-TZVP//SMD/M06-2X/6-31G(d)) = -2706.63722595

C	-0.96406400	0.46023100	2.10819800
C	-1.93583400	0.95575300	1.23148800
O	0.15767200	-0.05928300	1.76905100
B	0.55120700	-0.07460200	0.29156000
C	1.67701400	-1.24473500	0.17246900
C	1.72187700	-2.23386400	-0.80091200
C	2.73427600	-1.25681600	1.07776900
C	2.72619200	-3.19123600	-0.86827200
C	3.75575700	-2.19507900	1.04892800
C	3.74894300	-3.17296100	0.06590800
C	1.25088600	1.34532100	-0.09776800
C	1.42330100	2.44406800	0.73092900
C	1.81015700	1.47067000	-1.36538800
C	2.11267000	3.58816800	0.34142700
C	2.50012000	2.58915900	-1.79878900
C	2.65739600	3.65968100	-0.92860100
C	-0.83956100	-0.37972400	-0.51549200
C	-1.52679500	-1.56265800	-0.20534700
C	-1.44764300	0.41837900	-1.49348700
C	-2.76207900	-1.89070300	-0.72400800
C	-2.68207800	0.11798400	-2.04700900
C	-3.35588800	-1.02278900	-1.63597900
F	2.80944400	-0.31648700	2.03390400
F	4.74343300	-2.16098200	1.94653600
F	4.72035600	-4.08311000	0.01726700
F	2.71551200	-4.12381300	-1.82340500
F	0.77150300	-2.30908600	-1.74626600
F	-0.90717800	1.57370200	-1.87875100
F	-3.26739300	0.95502000	-2.90244800
F	-4.57196000	-1.27734800	-2.09268200
F	-3.41731000	-2.97868800	-0.32443700
F	-1.01512700	-2.39173700	0.71137500
F	0.91119900	2.47222600	1.97582300
F	2.24921000	4.61618800	1.18227400
F	3.31839400	4.74883900	-1.31824700
F	3.00797600	2.65364800	-3.03133700
F	1.65053900	0.46567100	-2.24467600
O	-1.16573700	0.71681900	3.38216800
C	-0.03841400	0.56433000	4.27443300
C	-3.30734200	0.67455900	1.11825000

C	-3.91119700	-0.46258800	1.72441000
C	-4.10889100	1.52723000	0.31374700
C	-5.25964700	-0.71269400	1.55212100
H	-3.29275700	-1.13607400	2.31343600
C	-5.45627400	1.26370300	0.13412800
H	-3.63296600	2.37900900	-0.16471900
C	-6.02526600	0.14732600	0.75406000
H	-5.72091200	-1.57777900	2.01622800
H	-6.06569000	1.91242500	-0.48552800
H	-7.08115600	-0.06199200	0.60947300
H	-0.39066600	0.94091100	5.23261100
H	0.23025700	-0.49038600	4.34955700
H	0.80927300	1.14591400	3.91376000

6
E (SMD/M06-2X/6-31G(d)) = -2705.575105
G (SMD/M06-2X/6-31G(d)) = -2705.337091
E (SMD/M06-2X/def2-TZVP//SMD/M06-2X/6-31G(d)) = -2706.7325746

C	-1.02185800	-1.93286300	0.92169100
C	-1.81564400	-1.48141700	-0.07622000
O	0.31362500	-1.68508700	0.98725200
B	0.90960100	-0.45180900	0.98287100
C	2.46565300	-0.44729100	0.80963900
C	3.16166300	-1.54866800	0.29802500
C	3.25098600	0.65924100	1.14773200
C	4.53758500	-1.54964500	0.12182300
C	4.62648900	0.68639700	0.99008700
C	5.27186900	-0.42699900	0.47149600
C	0.04740200	0.86089200	1.12981100
C	-1.01807800	0.96366300	2.01764400
C	0.24791400	1.95193100	0.28864300
C	-1.84976200	2.07114900	2.07242800
C	-0.56844400	3.07110000	0.30134700
C	-1.62016500	3.13077000	1.20651100
C	-1.21533600	-0.75464200	-1.21379300
C	-0.14004900	-1.25193800	-1.95091800
C	-1.69285200	0.50353300	-1.58746500
C	0.45871700	-0.52220000	-2.97040900
C	-1.12228700	1.24385700	-2.60902000
C	-0.03842000	0.72712000	-3.30662300
F	2.69332000	1.75805300	1.66203300
F	5.33077200	1.76200700	1.33422900
F	6.58745100	-0.41709400	0.31198600
F	5.15627400	-2.61829700	-0.37518800
F	2.52357200	-2.66049800	-0.05873000
F	-2.68250100	1.06644100	-0.88946000
F	-1.57454600	2.46516400	-2.88960700

F	0.52697000	1.43346300	-4.28000800
F	1.49276300	-1.02840700	-3.64023100
F	0.36508400	-2.45314200	-1.66918400
F	-1.28390600	-0.04362000	2.86164200
F	-2.86004900	2.12132200	2.93682500
F	-2.41024000	4.19535500	1.23539000
F	-0.36398100	4.07237400	-0.55058300
F	1.22968500	1.91756700	-0.62017400
O	-1.47740300	-2.73048200	1.88560800
C	-0.61128800	-3.06278100	2.97554700
C	-3.28705000	-1.67258300	-0.05231600
C	-4.02455400	-1.47975200	1.12397100
C	-3.97253200	-1.98919100	-1.23150600
C	-5.40763800	-1.62865100	1.12312300
H	-3.50695900	-1.19837200	2.03609700
C	-5.35734800	-2.13313300	-1.23158400
H	-3.41429800	-2.12853000	-2.15393300
C	-6.08004600	-1.95828300	-0.05327300
H	-5.96387500	-1.47387500	2.04310000
H	-5.87153900	-2.38399900	-2.15471600
H	-7.16007700	-2.07009500	-0.05248500
H	-1.22094200	-3.67011200	3.64325200
H	0.24708900	-3.64020500	2.62561300
H	-0.27273100	-2.16510300	3.49703500

$TS_{2\text{-}3}$

E (SMD/M06-2X/6-31G(d)) = -2814.9467434

G (SMD/M06-2X/6-31G(d)) = -2814.706040

E (SMD/M06-2X/def2-TZVP//SMD/M06-2X/6-31G(d)) = -2816.14903931

C	-0.68387000	1.01596500	2.16652100
C	-1.72558400	1.55312500	1.38926900
O	0.16365300	0.17809800	1.69648100
B	0.47239800	-0.17665100	0.22555500
C	1.86188500	-1.04089500	0.31667900
C	2.12209000	-2.26048000	-0.29892200
C	2.94601300	-0.49498800	1.00015200
C	3.34493600	-2.91555400	-0.21046500
C	4.18160600	-1.11362100	1.11666500
C	4.38183000	-2.34072000	0.50441600
C	0.85099400	1.17009800	-0.62096000
C	1.13135500	2.43235900	-0.11219800
C	1.10123100	1.02623700	-1.98339600
C	1.56497600	3.49375400	-0.89960100
C	1.53403200	2.05217400	-2.80596200
C	1.76717300	3.30429600	-2.25512200
C	-0.80507200	-1.03192200	-0.32135200
C	-1.11559900	-2.23267800	0.31350700

C	-1.71071700	-0.64556700	-1.30117000
C	-2.22243200	-3.00816000	0.00945300
C	-2.83541500	-1.38941300	-1.63906500
C	-3.09564300	-2.57594800	-0.97730600
F	2.83416500	0.71450200	1.57781600
F	5.17649200	-0.53512700	1.79353800
F	5.55977900	-2.95555000	0.59708500
F	3.52867500	-4.09217900	-0.81410300
F	1.18571200	-2.88691000	-1.02678400
F	-1.58560700	0.52584000	-1.94420500
F	-3.69276200	-0.94284800	-2.56042900
F	-4.19485700	-3.27322900	-1.25745900
F	-2.46706400	-4.14566700	0.66486600
F	-0.30990500	-2.69633900	1.28257000
F	1.00392000	2.71079800	1.19990000
F	1.80149500	4.68886200	-0.35253800
F	2.18730400	4.30924100	-3.02222800
F	1.74095300	1.84847100	-4.10870200
F	0.92004900	-0.17556200	-2.55681200
O	-0.47503700	1.46874200	3.37941200
C	0.80238100	1.18146100	3.99087000
C	-2.98271000	1.00796500	0.98295500
C	-3.53762400	-0.13181000	1.60569500
C	-3.66350200	1.61573700	-0.09441200
C	-4.73161900	-0.66194600	1.14059200
H	-3.01337700	-0.58906300	2.44071900
C	-4.86485800	1.09104500	-0.54279000
H	-3.22018300	2.48726300	-0.56635500
C	-5.39170600	-0.04893900	0.07152200
H	-5.15215700	-1.54905900	1.60340000
H	-5.38646000	1.55069000	-1.37541900
H	-6.32579000	-0.46861200	-0.29034000
H	0.76885800	1.69842300	4.94777500
H	0.91140700	0.10640700	4.13756800
H	1.60656000	1.56537300	3.36537500
N	-2.19184200	3.09561800	2.47314700
N	-2.46527600	4.15423000	2.59489000

TS_{1-7}

E (SMD/M06-2X/6-31G(d)) = -2814.95923088

G (SMD/M06-2X/6-31G(d)) = -2814.713958

E (SMD/M06-2X/def2-TZVP//SMD/M06-2X/6-31G(d)) = -2816.15671545

C	1.25726200	1.56295500	2.07890500
O	2.43170500	1.77498500	1.93326700
C	0.63742000	0.19278500	2.14996100
N	-0.36561800	0.20494800	3.07600300
N	-1.18734400	0.22323200	3.81856600

B	-0.10985500	-0.08847300	-0.13593000
C	-0.90641800	1.25941700	-0.43549000
C	-0.74791700	1.97355100	-1.62285400
C	-1.95826700	1.68210200	0.37056900
C	-1.53146700	3.07104500	-1.95264700
C	-2.75395500	2.77921600	0.08737100
C	-2.53369300	3.48213900	-1.08816500
C	-1.09112500	-1.35633400	-0.19301200
C	-1.38558200	-1.75281100	-1.49796700
C	-1.75905000	-2.05906800	0.79610900
C	-2.25589300	-2.78007300	-1.81516200
C	-2.63918600	-3.10067000	0.52358900
C	-2.89086800	-3.46363600	-0.78761100
C	1.38197700	-0.26108900	-0.63261200
C	1.96054400	-1.51097900	-0.88051100
C	2.23790700	0.82977200	-0.82267900
C	3.28183000	-1.67214300	-1.26894400
C	3.56345800	0.70653300	-1.20151800
C	4.08924600	-0.55693400	-1.42680300
F	-2.23772500	1.00362300	1.49359200
F	-3.72916500	3.14981300	0.91573500
F	-3.28855300	4.53218400	-1.39024300
F	-1.33537600	3.72200300	-3.09833000
F	0.16506900	1.60585200	-2.52681600
F	1.79783600	2.07863400	-0.63044500
F	4.32993700	1.78400800	-1.35513100
F	5.35629500	-0.69702900	-1.79265400
F	3.77951900	-2.88803000	-1.48720900
F	1.25891800	-2.63800000	-0.73621400
F	-0.79080800	-1.10896500	-2.51649000
F	-2.48881400	-3.11299900	-3.08350000
F	-3.73280100	-4.45526000	-1.06196200
F	-3.24441100	-3.74466700	1.52086400
F	-1.58304200	-1.77308000	2.09473500
O	0.30305900	2.48583200	2.17021600
C	0.74195700	3.84678000	2.01495600
H	1.21556200	3.97028100	1.03952200
H	-0.16066800	4.45080600	2.08631500
H	1.44405500	4.10733900	2.80876600
C	1.55363717	-1.01508411	2.42051286
C	2.93157078	-0.83233354	2.54039876
C	1.00595528	-2.29175605	2.54581818
C	3.76163032	-1.92616162	2.78486354
H	3.36312321	0.17418681	2.44079420
C	1.83602629	-3.38588209	2.79133847
H	-0.08006903	-2.43594628	2.45153454
C	3.21368813	-3.20331942	2.91073592

H	4.84780779	-1.78228348	2.87874693
H	1.40387810	-4.39230619	2.89049843
H	3.86835313	-4.06560398	3.10354515

7

E (SMD/M06-2X/6-31G(d)) = -2814.96468024
G (SMD/M06-2X/6-31G(d)) = -2814.717258
E (SMD/M06-2X/def2-TZVP//SMD/M06-2X/6-31G(d)) = -2816.1620834

C	-0.36167600	-1.65813900	2.24218100
O	-1.24953600	-2.45930700	2.13565800
C	-0.48168700	-0.15468300	1.89715200
N	0.29219800	0.45296100	2.94944300
N	0.82860200	0.90648500	3.79458200
B	0.13776100	0.00751600	0.18066700
C	1.62929100	-0.63832100	-0.07177300
C	1.96317900	-1.23356800	-1.29086900
C	2.71486600	-0.48361400	0.78382100
C	3.23426200	-1.69165700	-1.60839600
C	4.00080100	-0.93104200	0.51441000
C	4.26589400	-1.54610000	-0.69622200
C	0.27510100	1.55871100	-0.34505000
C	0.06184100	1.87270000	-1.69094000
C	0.80204000	2.61680300	0.38587000
C	0.28203800	3.12720400	-2.24063200
C	1.03161000	3.89141200	-0.11408400
C	0.76822300	4.15278200	-1.44609400
C	-1.07479600	-0.82958400	-0.53910000
C	-2.25913100	-0.23552700	-0.97344200
C	-1.04553400	-2.21348900	-0.69603400
C	-3.31745400	-0.93744100	-1.53145100
C	-2.07757700	-2.95763300	-1.24730300
C	-3.22693600	-2.31210100	-1.67260200
F	2.56677400	0.15014400	1.96051300
F	4.97855900	-0.75425600	1.40529600
F	5.49104400	-1.97832200	-0.98401800
F	3.46889700	-2.26309500	-2.79100000
F	1.04508300	-1.39400500	-2.25457900
F	0.02089600	-2.91953700	-0.28121700
F	-1.97382800	-4.28319200	-1.36259400
F	-4.23402900	-3.00452900	-2.19864400
F	-4.42759000	-0.29945100	-1.90896300
F	-2.45191700	1.08415200	-0.84113900
F	-0.37073200	0.94316100	-2.55394400
F	0.03942900	3.34805800	-3.53321300
F	0.98540700	5.36130600	-1.95767000
F	1.51771400	4.84903800	0.67664800
F	1.12630900	2.45230900	1.68549900

O	0.87787600	-1.93703100	2.61873800
C	1.16395600	-3.33139400	2.83846400
H	0.95241700	-3.89280800	1.92736300
H	2.22334900	-3.37134700	3.08381700
H	0.55981300	-3.70929700	3.66487700
C	-1.86342000	0.50170200	2.02701900
C	-3.04677500	-0.22767600	1.88011200
C	-1.93185100	1.88620400	2.21659100
C	-4.27382500	0.43231400	1.90764500
H	-3.01950500	-1.29686500	1.72308500
C	-3.16157200	2.53387200	2.25512300
H	-1.02974600	2.47710400	2.34038700
C	-4.33921900	1.80940700	2.09348400
H	-5.18403600	-0.14568100	1.78023800
H	-3.19177200	3.60835100	2.40479000
H	-5.29968200	2.31466500	2.11273700

$TS_{7\text{-}5}$

E (SMD/M06-2X/6-31G(d)) = -2814.95641647

G (SMD/M06-2X/6-31G(d)) = -2814.713112

E (SMD/M06-2X/def2-TZVP//SMD/M06-2X/6-31G(d)) = -2816.15675456

C	-0.57542800	-1.86571600	1.99651300
O	-0.62285000	-2.74650600	1.17981600
C	-0.60384200	-0.37390600	1.60138000
N	0.41399100	0.22541600	3.01859500
N	0.87310900	0.63116600	3.92728500
B	0.11802600	-0.02064500	0.11652800
C	1.56716600	-0.74682400	-0.11676300
C	2.12173300	-0.66325000	-1.39355700
C	2.37556500	-1.38045300	0.81286800
C	3.36747800	-1.16253000	-1.73249200
C	3.63236400	-1.89982200	0.52425800
C	4.13512200	-1.78714600	-0.75912400
C	0.41167000	1.57490600	-0.18040000
C	-0.01159100	2.30062800	-1.29577400
C	1.33269100	2.26538200	0.60741400
C	0.35345200	3.62023900	-1.54036600
C	1.72565000	3.57930300	0.40542800
C	1.21843900	4.27302500	-0.68014100
C	-1.06791600	-0.66579800	-0.84843700
C	-2.29072800	-0.02405300	-1.05692900
C	-0.95617700	-1.89308100	-1.50700300
C	-3.32441900	-0.53959700	-1.82433200
C	-1.96476000	-2.44041600	-2.28966000
C	-3.16184900	-1.76404000	-2.44852500
F	1.96874700	-1.53624100	2.09324300
F	4.35336500	-2.49580100	1.47582800

F	5.33720900	-2.27462600	-1.05943700
F	3.83374000	-1.05412400	-2.97764900
F	1.41312200	-0.08812300	-2.37915000
F	0.14800300	-2.64189900	-1.42311000
F	-1.78624400	-3.62128900	-2.88722400
F	-4.13703900	-2.28045900	-3.19204200
F	-4.46448200	0.14005500	-1.96994800
F	-2.52940700	1.18082300	-0.52038000
F	-0.77377400	1.75393800	-2.25071000
F	-0.10779000	4.25320500	-2.62061100
F	1.57791500	5.53384400	-0.90666800
F	2.59885200	4.16345000	1.22785800
F	1.93631300	1.63517500	1.63417300
O	-0.55422100	-2.04313100	3.30848900
C	-0.48657300	-3.41485000	3.74045500
H	0.40290400	-3.88821800	3.32029300
H	-0.42679900	-3.37292900	4.82600100
H	-1.38308400	-3.94941600	3.42180700
C	-1.88500500	0.28514700	1.99640000
C	-1.95393900	1.66682700	2.21725900
C	-3.05056100	-0.48732100	2.05421400
C	-3.17565900	2.26508200	2.48809500
H	-1.05141800	2.27109200	2.19381200
C	-4.27853400	0.12630800	2.28486400
H	-3.01509900	-1.55885000	1.87552700
C	-4.34190400	1.49859300	2.50720300
H	-3.22083000	3.33318500	2.67389400
H	-5.18170900	-0.47471300	2.30167500
H	-5.29864600	1.97291000	2.70211600

TS_{5-8}

E (SMD/M06-2X/6-31G(d)) = -3069.17425587
G (SMD/M06-2X/6-31G(d)) = -3068.809988
E (SMD/M06-2X/def2-TZVP//SMD/M06-2X/6-31G(d)) = -3070.45912399

C	1.78928500	0.86027300	-0.70341400
O	1.40623400	1.94006500	-1.08900700
C	1.04425800	0.07989100	0.37408000
B	-0.57886100	-0.10757500	-0.02307500
C	-1.28014600	0.94679100	-1.07479500
C	-1.96359500	0.59473100	-2.23700800
C	-1.41702200	2.28776800	-0.73064300
C	-2.66044300	1.50567600	-3.02574700
C	-2.09443200	3.23152200	-1.48370600
C	-2.72367900	2.83558800	-2.65369700
C	-1.71316900	-0.20853300	1.15123500
C	-2.89807700	-0.89705100	0.89293400
C	-1.73259500	0.53797400	2.32169400

C	-3.99525800	-0.89493800	1.73855300
C	-2.80744900	0.57071400	3.20269700
C	-3.95066800	-0.15025100	2.90832100
C	-0.22373200	-1.55146700	-0.77298900
C	-0.38910000	-2.79637400	-0.16340800
C	0.41925600	-1.59722100	-2.00909200
C	0.04379800	-3.98695000	-0.72489400
C	0.87413600	-2.76451200	-2.60452000
C	0.68867500	-3.97225700	-1.95351300
F	-0.88907900	2.72218500	0.42282200
F	-2.16247900	4.50555800	-1.08498300
F	-3.38970500	3.71869300	-3.39713100
F	-3.27916200	1.09677700	-4.13722400
F	-2.00741600	-0.67168500	-2.67983500
F	0.63282300	-0.46579000	-2.70225000
F	1.48361300	-2.73107100	-3.79060200
F	1.11547700	-5.10636400	-2.50162200
F	-0.14936100	-5.14610100	-0.09400400
F	-0.97206600	-2.90037600	1.03809700
F	-3.02032800	-1.60221500	-0.24226100
F	-5.09482600	-1.58712000	1.43387500
F	-4.99442800	-0.13169100	3.73563600
F	-2.74415600	1.29549200	4.32213000
F	-0.65830100	1.25974200	2.69246600
O	2.85031500	0.24294500	-1.21972900
C	3.47621500	0.91665500	-2.31898700
H	3.80683700	1.91074200	-2.01359900
H	4.32760400	0.29598100	-2.59446900
H	2.77299600	0.99718700	-3.15086800
C	1.69272800	-0.98395900	1.17277800
C	1.07752300	-1.37968500	2.37994000
C	2.83663800	-1.70192400	0.76289700
C	1.57776700	-2.42147000	3.14381900
H	0.21786900	-0.83361900	2.74337700
C	3.31328200	-2.77153700	1.51631400
H	3.33199900	-1.45950400	-0.16450300
C	2.69579100	-3.13420600	2.70890900
H	1.08790700	-2.68423100	4.07601000
H	4.17959000	-3.32124000	1.16134200
H	3.07891500	-3.96360900	3.29544600
C	3.43912400	1.74492800	1.52513800
C	3.55248700	2.95318000	0.80259600
C	4.70623800	3.32271100	0.11803700
C	5.77899200	2.43820300	0.17124600
C	5.69723900	1.23657700	0.89396300
C	4.54015200	0.88189200	1.57947200
C	2.08795700	1.69475300	2.04186200

C	1.50036200	2.91643300	1.69877600
H	4.76394700	4.25865500	-0.42888100
H	6.69919200	2.68739600	-0.34795100
H	6.56060600	0.57920600	0.92696500
H	4.49437800	-0.03697000	2.15518800
H	1.71282600	1.04214600	2.81191700
H	0.52842700	3.30127700	1.95956000
N	2.34680200	3.63137300	0.94670000
H	2.12051400	4.51288700	0.49971100

8

E (SMD/M06-2X/6-31G(d)) = -2705.43966576

G (SMD/M06-2X/6-31G(d)) = -2705.207445

E (SMD/M06-2X/def2-TZVP//SMD/M06-2X/6-31G(d)) = -2706.60023944

C	-1.92356000	0.00927700	-1.39667000
O	-1.87894700	-0.87928500	-2.23394800
C	-1.37128800	-0.21861200	0.01689600
B	0.39722000	0.01486500	-0.07575700
C	1.26114700	-0.96064900	-1.11392400
C	2.17482300	-0.55355600	-2.09080800
C	1.34590600	-2.32767200	-0.84521200
C	2.97279400	-1.43070300	-2.82077000
C	2.11654700	-3.24279100	-1.54425100
C	2.93910600	-2.78814300	-2.56083400
C	1.29796500	-0.24539900	1.31208400
C	2.56327300	0.35803800	1.35500000
C	1.10323500	-1.12905800	2.36966300
C	3.49468600	0.19071300	2.36587600
C	2.00448200	-1.33009300	3.41247700
C	3.21145400	-0.66154000	3.42090300
C	0.45970600	1.61318800	-0.51537700
C	0.61017800	2.66641900	0.38522800
C	0.19903700	2.03721600	-1.81442600
C	0.51908300	4.00825400	0.04039900
C	0.07723600	3.36112800	-2.20802800
C	0.24524000	4.36459600	-1.26889800
F	0.65466200	-2.84416800	0.19498000
F	2.09085400	-4.53963900	-1.22664300
F	3.69933400	-3.63479700	-3.25255600
F	3.79975600	-0.95888900	-3.75668500
F	2.39857100	0.73708300	-2.37327900
F	-0.00897900	1.13111200	-2.78944000
F	-0.21142600	3.66719400	-3.47750600
F	0.13321100	5.64670600	-1.61596400
F	0.65016400	4.95607200	0.97413100
F	0.80244700	2.43527100	1.69386900
F	2.96505300	1.15722100	0.35100100

F	4.66690400	0.82944000	2.32190100
F	4.09057200	-0.84454800	4.40522300
F	1.71083900	-2.19070200	4.39176200
F	0.01012200	-1.91430800	2.46495800
O	-2.48166000	1.17701200	-1.67037200
C	-2.97896400	1.34908000	-3.00607800
H	-3.77568100	0.62904100	-3.20255400
H	-3.36607400	2.36655900	-3.03935800
H	-2.17139600	1.21877700	-3.72660700
C	-1.83826400	0.76074500	1.11500300
C	-1.84772600	0.32983100	2.45217700
C	-2.18585300	2.10453700	0.89592900
C	-2.18185200	1.17631200	3.50560300
H	-1.61371900	-0.69565100	2.69874800
C	-2.52111500	2.95537400	1.94854700
H	-2.17998700	2.52010200	-0.10033500
C	-2.52218400	2.50299900	3.26273400
H	-2.17455800	0.78683400	4.51949100
H	-2.77550800	3.98789400	1.72529300
H	-2.78004000	3.16965400	4.08002100
C	-3.53000900	-1.69208400	0.44815300
C	-4.01097500	-2.72090700	-0.36639400
C	-5.33675600	-3.10005300	-0.47109800
C	-6.24884500	-2.37765500	0.29547200
C	-5.81273400	-1.33703700	1.11556400
C	-4.46218300	-0.98686800	1.20612700
C	-2.00277900	-1.63487700	0.29684800
C	-1.80569300	-2.65044700	-0.79337300
H	-5.64290100	-3.91263600	-1.12146800
H	-7.30400700	-2.62620400	0.24946400
H	-6.53761400	-0.78103500	1.70145600
H	-4.16857100	-0.17535900	1.85860100
H	-1.53693700	-2.08807400	1.17816100
H	-0.89172500	-2.97266200	-1.26147600
N	-2.90828600	-3.27742000	-1.05624000
H	-2.98398600	-4.03624700	-1.73143500

$(H_2O)_3$
E (SMD/M06-2X/6-31G(d)) = -229.167064366
G (SMD/M06-2X/6-31G(d)) = -229.125684
E (SMD/M06-2X/def2-TZVP//SMD/M06-2X/6-31G(d)) = -229.316594783

O	1.09731800	-1.36969400	0.01130400
H	1.27040200	-0.41589700	-0.08785400
H	0.13090600	-1.37890900	0.09438100
O	0.72643000	1.45440200	-0.10624900
H	-0.18173400	1.09401700	-0.07103700
H	0.89271500	1.76961800	0.79536700

O	-1.55251000	-0.17570600	0.01430400
H	-2.21415400	-0.08551200	0.71924200
H	-2.06803800	-0.25533500	-0.80497000

$(H_2O)_3H^+$
E (SMD/M06-2X/6-31G(d)) = -229.599155882
G (SMD/M06-2X/6-31G(d)) = -229.545852
E (SMD/M06-2X/def2-TZVP//SMD/M06-2X/6-31G(d)) = -229.740937092

H	-2.08011700	-1.27682300	-0.39524800
O	-2.13031800	-0.36548200	-0.05364700
H	-2.59094200	-0.42405700	0.80359200
H	-0.88388500	0.32043500	0.03004400
O	0.00079200	0.90031500	0.07965400
H	0.00134300	1.53595400	-0.66604400
H	0.88078400	0.31719200	0.03003800
O	2.11273200	-0.40996100	-0.07992500
H	2.78184200	-0.10254300	0.55930300
H	2.02532800	-1.36913800	0.06966200

$TS_{8\text{-}9}$
E (SMD/M06-2X/6-31G(d)) = -3298.3784198
G (SMD/M06-2X/6-31G(d)) = -3297.947061
E (SMD/M06-2X/def2-TZVP//SMD/M06-2X/6-31G(d)) = -3299.80455391

C	1.59385900	0.57862400	1.01027700
O	2.05964100	1.63937600	1.39427600
C	1.04305600	0.24116000	-0.37278100
B	-0.70867800	0.08181200	0.04948600
C	-1.16221200	-1.19982800	1.00271400
C	-2.42227000	-1.13773800	1.60202900
C	-0.53599000	-2.42894500	1.16799300
C	-2.99648200	-2.15708100	2.34414400
C	-1.06916300	-3.48211700	1.90475900
C	-2.30848500	-3.34945600	2.50238100
C	-1.80214500	-0.11897700	-1.18824700
C	-2.90317600	0.69158100	-1.47678300
C	-1.82520500	-1.32306200	-1.89832200
C	-3.83431600	0.41198400	-2.47426300
C	-2.72510400	-1.64501600	-2.90147300
C	-3.74156700	-0.75549700	-3.20750500
C	-0.92334800	1.52534800	0.85918200
C	-0.93962300	2.76774200	0.21762200
C	-0.91873600	1.63543400	2.25030200
C	-0.92023400	3.99072500	0.87303900
C	-0.89206800	2.83619400	2.94876400
C	-0.88958900	4.03160000	2.25629800
F	0.64414100	-2.69815800	0.58924500
F	-0.39595000	-4.63305000	2.01880700

F	-2.83669400	-4.35146100	3.20748800
F	-4.20317700	-2.00304200	2.89946000
F	-3.16358600	-0.02065900	1.48237000
F	-0.89562700	0.54760000	3.04356200
F	-0.85269600	2.83514100	4.28526900
F	-0.86230600	5.19693500	2.90282800
F	-0.93522600	5.13139600	0.17647000
F	-0.99354800	2.86040300	-1.11994800
F	-3.18828600	1.79386800	-0.77095100
F	-4.84104900	1.25887100	-2.70426500
F	-4.62752500	-1.03564700	-4.16191000
F	-2.63945800	-2.81322900	-3.54419100
F	-0.94470400	-2.29937700	-1.59724900
O	1.45234500	-0.44160700	1.85261700
C	1.88379800	-0.22888800	3.19979500
H	1.42762000	0.67034900	3.61532200
H	1.55821200	-1.11130000	3.74932300
H	2.97307900	-0.13972500	3.24341000
C	1.23762600	1.36474100	-1.41388500
C	0.65621000	1.16638700	-2.67785100
C	2.09228500	2.46660300	-1.27602100
C	0.90206400	2.01964600	-3.74721300
H	-0.00689200	0.32414700	-2.83758700
C	2.33567500	3.33126500	-2.34629800
H	2.60392400	2.65176400	-0.34441700
C	1.74854800	3.11696300	-3.58821100
H	0.43170300	1.82476800	-4.70661800
H	3.00758000	4.17233200	-2.20050700
H	1.94745000	3.78743300	-4.41863600
C	3.07700800	-1.60038300	-0.25633200
C	3.31487700	-2.79542100	-0.95306300
C	4.37541000	-3.65856700	-0.68196000
C	5.24272700	-3.28391800	0.33077900
C	5.06253800	-2.06999800	1.01678700
C	4.00388300	-1.22068700	0.72438300
C	1.94254200	-0.91626300	-0.93183100
C	1.55737000	-1.83613900	-1.94863900
H	4.51354500	-4.57228500	-1.25077000
H	6.08111400	-3.92425000	0.58468500
H	5.77079100	-1.78467800	1.78856400
H	3.92639300	-0.27214400	1.24471400
H	2.73852000	-0.36452600	-1.81599600
H	0.81644800	-1.71257500	-2.72257300
N	2.36347500	-2.88536200	-1.96710800
H	2.34832800	-3.60680600	-2.68217300
O	3.70855100	0.16738700	-2.67258600
H	3.30167700	0.93465900	-3.11712500

H	4.41364200	0.54428300	-2.05444300
O	5.51978400	1.01286800	-0.99646700
H	5.95977800	0.21187900	-0.66764600
H	5.19317400	1.46408700	-0.17971600
O	4.78258900	2.10269900	1.37968800
H	5.04970700	3.02890400	1.49455700
H	3.82261300	2.08196300	1.55345300

9

E (SMD/M06-2X/6-31G(d)) = -3068.76590755

G (SMD/M06-2X/6-31G(d)) = -3068.409345

E (SMD/M06-2X/def2-TZVP//SMD/M06-2X/6-31G(d)) = -3070.05678867

C	-1.75327600	-0.50429100	1.28760000
O	-1.87543100	-1.58181800	1.83730800
C	-1.38960000	-0.21590000	-0.16819500
B	0.38715500	0.03001700	0.01869800
C	0.87062300	1.30285700	0.97581300
C	2.17018700	1.28111700	1.48927000
C	0.20688400	2.50357500	1.21455700
C	2.74830600	2.29971500	2.22980400
C	0.74601100	3.55238100	1.95592000
C	2.02307300	3.45414600	2.47400500
C	1.32825200	0.33690800	-1.32992700
C	2.48625000	-0.34269400	-1.72766000
C	1.16020300	1.53020300	-2.03553800
C	3.26803900	0.02044300	-2.82157900
C	1.90404900	1.93515500	-3.13307700
C	2.97020900	1.15741800	-3.54805900
C	0.81585500	-1.42799600	0.70918800
C	0.96426400	-2.61482300	-0.00991400
C	0.91919200	-1.61267600	2.08624000
C	1.19188500	-3.85763100	0.56614100
C	1.15356400	-2.82988900	2.70882600
C	1.29215300	-3.97154600	1.94102800
F	-1.00640600	2.75235700	0.71340200
F	0.03790900	4.67247400	2.14942000
F	2.55368400	4.45568000	3.17994300
F	3.99696400	2.18286200	2.69759500
F	2.96332000	0.21514400	1.26911500
F	0.72735400	-0.58293200	2.93481900
F	1.22123700	-2.90946900	4.04383400
F	1.50903000	-5.15793000	2.51397500
F	1.31451700	-4.94692000	-0.20269100
F	0.90221200	-2.63176900	-1.35019200
F	2.98956100	-1.38156100	-1.04490500
F	4.33794100	-0.71137700	-3.15018500
F	3.71594900	1.51807900	-4.59268300

F	1.62803100	3.08628200	-3.75490500
F	0.24929800	2.42669200	-1.61788000
O	-1.89754500	0.63233600	1.98505300
C	-2.20732100	0.47508300	3.36629600
H	-1.45517400	-0.13943800	3.86419700
H	-2.21282600	1.48307200	3.78168200
H	-3.19212500	0.01457100	3.48842700
C	-1.57953200	-1.42020900	-1.11606600
C	-1.31852700	-1.19081600	-2.47759300
C	-2.00844300	-2.70286200	-0.75680700
C	-1.44438900	-2.19175200	-3.43041300
H	-1.00530000	-0.19957900	-2.79325000
C	-2.13901800	-3.71288100	-1.71472700
H	-2.22036500	-2.93169600	0.27773000
C	-1.85422100	-3.47099600	-3.05207300
H	-1.22222100	-1.97350000	-4.47156400
H	-2.46853600	-4.69930300	-1.39854300
H	-1.95390100	-4.26046400	-3.79155300
C	-3.86482900	0.64829600	-0.30994800
C	-4.58913000	1.60739400	-1.06356000
C	-5.97429200	1.77618400	-0.95962400
C	-6.65771800	0.94359500	-0.09011000
C	-5.97316200	-0.03907800	0.65168100
C	-4.59962300	-0.19411500	0.54898000
C	-2.45782400	0.77908700	-0.66384800
C	-2.42676300	1.75848200	-1.62174700
H	-6.48798900	2.53058100	-1.54871600
H	-7.73406900	1.04325700	0.01497700
H	-6.53420800	-0.69189500	1.31402800
H	-4.11187200	-0.97511300	1.12331300
H	-1.60586800	2.15901800	-2.18692100
N	-3.68618100	2.25886400	-1.85708600
H	-3.89762300	3.01438400	-2.49386200

$TS_{9\text{-}10}$

E (SMD/M06-2X/6-31G(d)) = -3068.74388018

G (SMD/M06-2X/6-31G(d)) = -3068.390887

E (SMD/M06-2X/def2-TZVP//SMD/M06-2X/6-31G(d)) = -3070.04029122

C	-0.04464100	-0.39459600	-2.04631100
O	0.18960500	0.79785800	-1.76992000
C	0.68538200	-1.52485300	-1.61294200
B	-0.81926100	0.82889400	1.04189300
C	-1.43499800	2.12958500	0.40796100
C	-1.08781000	3.41086700	0.82688000
C	-2.31634000	2.06782000	-0.67079500
C	-1.57056800	4.56234700	0.22259400
C	-2.79995300	3.19292900	-1.31509000

C	-2.42536500	4.45018500	-0.86209000
C	0.66539700	0.84809600	1.53939100
C	1.12168100	0.04526500	2.58894300
C	1.65204800	1.60434300	0.89510200
C	2.45847400	-0.08678800	2.91956900
C	2.99970600	1.49776000	1.20483200
C	3.40620600	0.62929300	2.20226500
C	-1.72439300	-0.45441300	1.21653900
C	-1.22963700	-1.76430700	1.24994600
C	-3.11851300	-0.36042400	1.32786700
C	-2.03980500	-2.88734000	1.33976400
C	-3.95646900	-1.45790600	1.42747400
C	-3.41281900	-2.73387700	1.42232500
F	-2.73429400	0.88785500	-1.13429700
F	-3.61503900	3.07944700	-2.36580700
F	-2.89012000	5.54116000	-1.46211200
F	-1.21299600	5.76830700	0.66755300
F	-0.24908900	3.58352500	1.85530700
F	-3.72489000	0.83154700	1.36357800
F	-5.27626500	-1.29821300	1.53344000
F	-4.20324800	-3.79697200	1.50913000
F	-1.50413700	-4.10556200	1.35050900
F	0.07541100	-2.01431100	1.20054700
F	0.25560900	-0.64955100	3.33620100
F	2.84519800	-0.89716700	3.90767800
F	4.69899400	0.48134300	2.47260200
F	3.89974300	2.22220000	0.54343600
F	1.35592300	2.48460200	-0.05397900
O	-1.09054400	-0.66488400	-2.92246300
C	-1.47258500	0.41353900	-3.75142300
H	-0.67581400	0.65923000	-4.46602900
H	-2.35324000	0.07613500	-4.30355600
H	-1.71829100	1.31418000	-3.18794000
C	0.17239100	-2.88716100	-1.72644200
C	1.02998100	-4.01313800	-1.79654000
C	-1.21535800	-3.18299600	-1.73175000
C	0.54579500	-5.31372000	-1.87625000
H	2.10414000	-3.85329200	-1.79872100
C	-1.69675900	-4.48404500	-1.81774500
H	-1.92197500	-2.36333200	-1.65771700
C	-0.82560600	-5.57185100	-1.88822500
H	1.25324700	-6.13796000	-1.93704200
H	-2.77245100	-4.64959500	-1.80693600
H	-1.20290700	-6.58850400	-1.94568600
C	3.05777400	-0.38214800	-1.53406600
C	4.23570000	-0.60492500	-0.77273000
C	5.42876900	0.09179900	-0.99823300

C	5.43881500	1.02726800	-2.01949200
C	4.28255600	1.27316000	-2.78859600
C	3.10164700	0.58529700	-2.55331700
C	2.02743100	-1.28665400	-1.04410900
C	2.63427300	-1.97607000	-0.01577200
H	6.30978900	-0.09946600	-0.39130800
H	6.34741400	1.58646800	-2.22475400
H	4.32056400	2.01959900	-3.57719900
H	2.20959000	0.79731800	-3.13283100
H	2.23701900	-2.76672900	0.60518100
N	3.95007100	-1.58321900	0.14262300
H	4.59460000	-1.95772000	0.82448700

10

E (SMD/M06-2X/6-31G(d)) = -3068.80615816

G (SMD/M06-2X/6-31G(d)) = -3068.454672

E (SMD/M06-2X/def2-TZVP//SMD/M06-2X/6-31G(d)) = -3070.10024443

C	-1.13833700	0.05301900	-1.47035900
O	0.13907900	-0.23737100	-1.26772700
C	-2.14426900	-0.86109500	-1.50296700
B	0.99741500	0.26206200	-0.14393200
C	2.38112700	0.92621900	-0.73948900
C	3.31096200	1.44921200	0.15437700
C	2.80331100	0.89592100	-2.06283000
C	4.55353100	1.93867100	-0.20985900
C	4.04419600	1.37579900	-2.47533000
C	4.92507400	1.89919200	-1.54629600
C	1.54035600	-1.07692400	0.66916100
C	1.57006400	-1.29127100	2.04194200
C	2.08920800	-2.11330000	-0.08251400
C	2.03425900	-2.46423300	2.62734400
C	2.54925600	-3.30573200	0.45488500
C	2.51899000	-3.48522200	1.82852800
C	0.12515400	1.28205800	0.80699300
C	-0.93799100	0.78009400	1.55419600
C	0.28316100	2.65933900	0.90065100
C	-1.76730200	1.55677300	2.34812100
C	-0.53162400	3.48167900	1.67146900
C	-1.56847100	2.92753400	2.40004400
F	2.03049000	0.39797900	-3.03954400
F	4.39466600	1.32695400	-3.76551500
F	6.11798300	2.36032700	-1.92853000
F	5.39462900	2.43876600	0.70141500
F	3.00045900	1.50298300	1.46302100
F	1.25255100	3.30098700	0.22167400
F	-0.32174600	4.80159800	1.70992800
F	-2.35913900	3.69711600	3.14850100

F	-2.75735800	1.00298700	3.05825500
F	-1.19511400	-0.54005300	1.54750500
F	1.13873100	-0.35608500	2.90532800
F	2.01891700	-2.61278200	3.95639200
F	2.96200400	-4.61996200	2.37307100
F	3.01810900	-4.27820900	-0.33578000
F	2.20048100	-1.99366300	-1.41844900
O	-1.44745900	1.35975100	-1.68503600
C	-0.48647700	2.12680900	-2.40174700
H	-0.19567100	1.62465800	-3.32944700
H	-0.98032900	3.07197500	-2.63460500
H	0.40583300	2.32925200	-1.80358300
C	-1.94785400	-2.28084600	-1.12704300
C	-3.00022500	-3.00399600	-0.53846400
C	-0.74358000	-2.96673400	-1.35783400
C	-2.84338600	-4.33559600	-0.16392100
H	-3.95103000	-2.50921100	-0.36123800
C	-0.58420300	-4.29580400	-0.97543800
H	0.07220600	-2.44782500	-1.84268800
C	-1.63064900	-4.99095300	-0.37170600
H	-3.67440000	-4.86143300	0.29855000
H	0.36924400	-4.78735600	-1.15624700
H	-1.50597300	-6.02777400	-0.07333100
C	-4.47882400	0.12304000	-0.87352800
C	-5.64599700	0.46974800	-1.59589900
C	-6.77198700	1.01427800	-0.96519000
C	-6.70642900	1.20245200	0.40679500
C	-5.55576300	0.85050700	1.14523700
C	-4.44750800	0.30866300	0.51638500
C	-3.51794800	-0.39160400	-1.81611500
C	-4.13489000	-0.34553300	-3.03909900
H	-7.66068100	1.27865700	-1.53097200
H	-7.56167100	1.62592400	0.92539000
H	-5.54231600	1.00667900	2.21998300
H	-3.56676900	0.01145800	1.07763800
H	-3.75847500	-0.63684000	-4.01052300
N	-5.40654600	0.16975300	-2.91414500
H	-6.05476200	0.31074400	-3.67662100

11

E (SMD/M06-2X/6-31G(d)) = -3298.44170559

G (SMD/M06-2X/6-31G(d)) = -3298.010070

E (SMD/M06-2X/def2-TZVP//SMD/M06-2X/6-31G(d)) = -3299.86578978

C	-1.15492300	0.53662800	-0.98307100
O	0.05829200	-0.03645800	-0.97237200
C	-2.24723000	-0.05962400	-1.48990900
B	1.02130900	0.01754500	0.19965900

C	1.69029900	-1.47232500	0.40278700
C	2.57401200	-1.65166700	1.46352500
C	1.44379200	-2.61587000	-0.34414600
C	3.16474700	-2.86257100	1.78630100
C	2.01155200	-3.85312700	-0.05726200
C	2.87599000	-3.97940700	1.01598600
C	0.21178700	0.27765000	1.60485400
C	0.56825300	1.13302000	2.64235100
C	-0.89930900	-0.52168000	1.87739800
C	-0.17734500	1.27547600	3.80750800
C	-1.66949000	-0.41612000	3.02701800
C	-1.31315100	0.50618900	3.99787700
C	2.19497900	1.12032000	-0.19625800
C	2.05894300	2.50027400	-0.04853000
C	3.38060400	0.75476600	-0.83562700
C	3.00219100	3.42851600	-0.46849000
C	4.36137300	1.64412700	-1.25676500
C	4.17157300	3.00148900	-1.07368000
F	0.65331100	-2.58153900	-1.43080600
F	1.73609900	-4.91765600	-0.81597000
F	3.43117400	-5.15734000	1.30245400
F	4.00433400	-2.96528100	2.81980700
F	2.90070300	-0.59683500	2.23295500
F	3.64149500	-0.53558400	-1.14451800
F	5.46201400	1.19995800	-1.86543600
F	5.08030200	3.87936200	-1.49016800
F	2.76382300	4.73659000	-0.32492700
F	0.95730000	3.04069700	0.50026000
F	1.69415200	1.85894600	2.59106400
F	0.20744200	2.13253400	4.75701900
F	-2.03402500	0.62900000	5.11138100
F	-2.73793700	-1.19948400	3.20778600
F	-1.27110500	-1.47797000	1.00814400
O	-1.10744600	1.82143900	-0.50918100
C	-2.07669100	2.32333700	0.41468300
H	-1.54350000	3.03213500	1.05200500
H	-2.88741400	2.82841000	-0.11475600
H	-2.48194200	1.50933800	1.01866800
C	-2.14365600	-1.44008800	-2.05269100
C	-2.74566700	-2.53406800	-1.42259000
C	-1.47536500	-1.64803900	-3.26402300
C	-2.65622300	-3.80855600	-1.97745800
H	-3.27921200	-2.38578000	-0.48817100
C	-1.38232800	-2.92161600	-3.82016600
H	-1.02611500	-0.79937800	-3.77450900
C	-1.97000800	-4.00750100	-3.17476200
H	-3.12115700	-4.64968000	-1.47120800
H	-0.85227900	-3.06458600	-4.75760300

H	-1.89843900	-5.00271600	-3.60354700
C	-4.61118200	0.49172300	-0.54909700
C	-5.71733100	1.24187600	-1.01374400
C	-6.89905800	1.35990100	-0.27116600
C	-6.94824400	0.71610500	0.95478800
C	-5.85006700	-0.02510200	1.44199700
C	-4.68525200	-0.14102400	0.70273300
C	-3.56999000	0.60570200	-1.53997000
C	-4.08150900	1.39233800	-2.54061800
H	-7.74005200	1.93814900	-0.64219900
H	-7.84941900	0.78753300	1.55639200
H	-5.92119500	-0.50697800	2.41263400
H	-3.83375600	-0.70187700	1.08093000
H	-3.62389500	1.71103000	-3.46755000
N	-5.36260700	1.77867500	-2.22795100
H	-5.95564700	2.35369600	-2.81131600
H	-0.31230000	2.91085200	-1.53992000
O	0.18893400	3.41900400	-2.22780100
H	0.57331400	4.18770000	-1.77026600
H	1.17327200	2.60062400	-2.83560700
O	1.91317400	2.01275200	-3.32805100
H	1.91035900	2.24354600	-4.27886900
H	1.76492100	0.93790800	-3.22297100
O	1.57603400	-0.41291000	-3.11584800
H	2.38325900	-0.86565900	-2.80901500
H	0.90279700	-0.54820600	-2.39985500

$TS_{11\text{-}12}$

E (SMD/M06-2X/6-31G(d)) = -3298.41797374

G (SMD/M06-2X/6-31G(d)) = -3297.988212

E (SMD/M06-2X/def2-TZVP//SMD/M06-2X/6-31G(d)) = -3299.8428948

C	-1.08557100	0.12901800	-1.32417400
O	0.11445000	-0.29495000	-1.07132500
C	-2.18325700	-0.72627800	-1.55306300
B	1.01020200	0.18638000	0.11277700
C	2.38828100	-0.69736800	0.06831400
C	3.40890700	-0.34392500	0.94963600
C	2.62025200	-1.87175600	-0.63588800
C	4.58463400	-1.05861600	1.10364500
C	3.78572800	-2.62201700	-0.51608900
C	4.77533800	-2.21445000	0.35981800
C	0.28497000	-0.26563700	1.51546600
C	0.19232300	0.46458500	2.69803200
C	-0.21883900	-1.56486800	1.60105700
C	-0.48422400	0.00874100	3.82364300
C	-0.90956300	-2.05683600	2.69957000
C	-1.05559400	-1.25318900	3.81908100

C	1.27247700	1.79784400	-0.04455800
C	0.38341100	2.76745300	0.41939700
C	2.36343900	2.31789000	-0.73209300
C	0.58385100	4.13320700	0.27678800
C	2.60151100	3.67375400	-0.91528000
C	1.70641500	4.59279300	-0.39542400
F	1.71350300	-2.35645700	-1.50590500
F	3.94891400	-3.73550200	-1.23375100
F	5.89660300	-2.92169700	0.49017500
F	5.52468700	-0.65651000	1.96112600
F	3.26430800	0.75154800	1.71499900
F	3.26940200	1.49825600	-1.31308700
F	3.67525500	4.09206100	-1.58757600
F	1.91242000	5.89800100	-0.55100200
F	-0.30192000	5.00556900	0.76016900
F	-0.76417200	2.41441200	1.01376300
F	0.77864300	1.66334600	2.82849000
F	-0.56827900	0.77003700	4.91789800
F	-1.70213600	-1.70337300	4.89288300
F	-1.39284800	-3.30138500	2.70522600
F	-0.00066400	-2.42709900	0.60164600
O	-1.29597100	1.43341000	-1.50990900
C	-0.50676900	2.09204700	-2.52202000
H	-1.11040300	2.15394400	-3.43166500
H	-0.28237100	3.09698200	-2.16521300
H	0.41794600	1.54772500	-2.71716600
C	-2.02702600	-2.20097900	-1.33588300
C	-2.79602700	-2.87173000	-0.38055100
C	-1.11581200	-2.92838200	-2.10593200
C	-2.63189400	-4.23934500	-0.17860600
H	-3.51430800	-2.31448900	0.21580100
C	-0.95158600	-4.29553500	-1.90634300
H	-0.52177100	-2.41720800	-2.86119900
C	-1.70803100	-4.95476100	-0.93900600
H	-3.22353200	-4.74616700	0.57825100
H	-0.23341500	-4.84611900	-2.50710000
H	-1.58067500	-6.02155900	-0.78063600
C	-4.01115500	0.52334400	-0.17377000
C	-5.37707300	0.82800400	-0.37769000
C	-6.14657300	1.47543300	0.59711800
C	-5.51983500	1.79906800	1.78926500
C	-4.16240800	1.48558200	2.01798700
C	-3.40460100	0.85121300	1.04910800
C	-3.54114200	-0.13576500	-1.36518600
C	-4.62021700	-0.22068500	-2.21013500
H	-7.19343000	1.70629000	0.42458000
H	-6.08666500	2.30030900	2.56807800

H	-3.70890000	1.74714300	2.96959700
H	-2.35965300	0.60686000	1.22729600
H	-4.70381000	-0.67696500	-3.18818700
N	-5.71548700	0.36380000	-1.62662700
H	-6.63315100	0.42518000	-2.04725000
H	-2.05765600	-0.54109300	-2.94211400
O	-2.14361600	-0.40635200	-4.17662600
H	-2.72061300	0.36231900	-4.35233000
H	-1.21777300	-0.20010500	-4.59412800
O	0.11254300	0.18350800	-5.17531600
H	0.34068000	-0.36823700	-5.94239600
H	0.86468100	0.08684400	-4.53572900
O	1.98669200	-0.23499200	-3.31016200
H	2.52292000	0.44366700	-2.86778700
H	1.51703100	-0.69678600	-2.59436700

12

E (SMD/M06-2X/6-31G(d)) = -861.72755854

G (SMD/M06-2X/6-31G(d)) = -861.487880

E (SMD/M06-2X/def2-TZVP//SMD/M06-2X/6-31G(d)) = -862.056548326

C	-0.76190500	1.83162800	-0.26809900
O	-0.99330400	1.68843600	-1.44587100
C	-0.79562300	0.72855800	0.78785900
O	-0.38421700	2.99626300	0.27026300
C	-0.22378000	4.07496300	-0.65658300
H	-1.16748700	4.28454500	-1.16515800
H	0.08507800	4.93352900	-0.06180500
H	0.54122800	3.82920700	-1.39656000
C	-1.80143700	-0.35679800	0.42450600
C	-1.49456600	-1.71351300	0.52656600
C	-3.09429600	0.01132600	0.03313000
C	-2.45552200	-2.68350100	0.23770500
H	-0.49937300	-2.01865600	0.83486700
C	-4.05397300	-0.95289700	-0.25427300
H	-3.35131800	1.06483900	-0.04935700
C	-3.73614500	-2.30786600	-0.15486400
H	-2.19669600	-3.73507300	0.32055200
H	-5.05107100	-0.64723500	-0.55754500
H	-4.48332400	-3.06254800	-0.38149900
C	1.48879200	-0.32624600	-0.01343400
C	2.72898100	-0.57941600	0.62321000
C	3.81124800	-1.15671000	-0.05188100
C	3.62845000	-1.48154900	-1.38549800
C	2.40048000	-1.24157400	-2.03810600
C	1.33183000	-0.67018900	-1.36839200
C	0.62163700	0.25643700	0.98019300
C	1.35265300	0.33462600	2.13601800

H	4.75351300	-1.34201500	0.45502900
H	4.44635700	-1.93136600	-1.94035300
H	2.29578700	-1.51205300	-3.08450400
H	0.38789600	-0.48428100	-1.87270200
H	1.06442500	0.71630300	3.10634300
N	2.61297000	-0.16536600	1.92816800
H	3.33944800	-0.22236000	2.62912100
H	-1.11602100	1.20345200	1.72291300

13

E (SMD/M06-2X/6-31G(d)) = -723.906115802

G (SMD/M06-2X/6-31G(d)) = -723.738866

E (SMD/M06-2X/def2-TZVP//SMD/M06-2X/6-31G(d)) = -724.179841016

C	-0.50850900	-0.78563500	-0.11667000
C	0.51632700	0.26611100	-0.08342800
O	-0.19420500	-1.95838300	-0.25975000
N	0.10203300	1.50346600	-0.26692700
N	-0.21681900	2.57057200	-0.43687000
C	-1.95304600	-0.40060900	0.00346200
C	-2.87746400	-1.10523400	-0.77356200
C	-4.23367900	-0.81508900	-0.67815500
C	-4.67553000	0.16374200	0.21218700
C	-3.75974000	0.84937600	1.00782400
C	-2.39904800	0.57267600	0.90263800
H	-2.51965400	-1.87415000	-1.45154400
H	-4.94738900	-1.35415100	-1.29343300
H	-5.73530900	0.38670000	0.29105600
H	-4.10411800	1.59684700	1.71560900
H	-1.69372200	1.09408200	1.54479600
C	1.97882100	0.06448700	-0.01194400
C	2.86177000	1.06786000	-0.43671300
C	4.23729900	0.88613600	-0.34446800
C	4.75788600	-0.30071900	0.16666000
C	3.88542100	-1.30128700	0.58908600
C	2.50733000	-1.12653400	0.50658000
H	2.47838600	1.99793100	-0.84845800
H	4.90230100	1.67617000	-0.68011900
H	5.83175100	-0.44403100	0.23490900
H	4.27730200	-2.23010600	0.99292500
H	1.84056300	-1.91217700	0.83786400

$TS_{13\text{-}14}$

E (SMD/M06-2X/6-31G(d)) = -2931.41699392

G (SMD/M06-2X/6-31G(d)) = -2931.126074

E (SMD/M06-2X/def2-TZVP//SMD/M06-2X/6-31G(d)) = -2932.64796929

C	1.05335900	-1.86147600	1.44385900
O	0.64587100	-2.84107800	0.85481000

C	0.07812600	-0.75689700	1.80584000
N	0.63545000	0.07435500	2.75950700
N	1.03352700	0.75371800	3.53782600
B	-0.19177800	0.23624300	-0.20720200
C	1.26450700	0.64890800	-0.77952400
C	1.40029500	0.72473700	-2.16853600
C	2.37898900	1.08486800	-0.07525500
C	2.55748200	1.12919200	-2.81181400
C	3.56757200	1.48074000	-0.67767200
C	3.65920100	1.50587700	-2.05705600
C	-1.09000100	1.57260000	-0.05556900
C	-2.03510000	1.90779200	-1.03037200
C	-0.84798500	2.58627900	0.86452100
C	-2.72485400	3.11200200	-1.04814800
C	-1.52206100	3.79743600	0.89506000
C	-2.47451600	4.06411500	-0.07379000
C	-0.91123200	-1.04392600	-0.84221100
C	-2.28019800	-1.28885700	-0.69441300
C	-0.23431100	-2.02712800	-1.56781500
C	-2.92401000	-2.40687600	-1.20057000
C	-0.84205100	-3.15141900	-2.10355800
C	-2.19993700	-3.34744100	-1.91423800
F	2.38219400	1.11350500	1.26596400
F	4.61289700	1.83238200	0.07053200
F	4.78348300	1.89030800	-2.65376400
F	2.61891800	1.16465100	-4.14277700
F	0.36909000	0.38839700	-2.95705300
F	1.08726500	-1.94356100	-1.77140200
F	-0.12655800	-4.05001300	-2.78176300
F	-2.79844300	-4.42764100	-2.40428600
F	-4.22803200	-2.58827600	-0.98526300
F	-3.06161500	-0.43788100	-0.02163500
F	-2.31555300	1.07428300	-2.03929100
F	-3.61832500	3.36137500	-2.00510100
F	-3.13066300	5.21956400	-0.07540100
F	-1.24710600	4.70239500	1.83397000
F	0.09024400	2.41738900	1.81146600
C	-1.34408600	-1.04873100	2.24223300
C	-1.92715200	-2.30784000	2.07104600
C	-2.10459800	-0.01427800	2.80295800
C	-3.25767900	-2.51031900	2.43469300
H	-1.36066300	-3.11901800	1.63415100
C	-3.42456600	-0.23056700	3.17846600
H	-1.67355500	0.97348500	2.94697400
C	-4.01117100	-1.47961200	2.98698300
H	-3.70149100	-3.48965800	2.28226700
H	-3.99582400	0.58467500	3.61123200

H	-5.04654800	-1.64665800	3.26684400
C	2.52139700	-1.70561500	1.71472400
C	3.04051100	-1.54718700	3.00260000
C	3.38469300	-1.80228100	0.61871900
C	4.41683300	-1.44278500	3.18447600
H	2.38450300	-1.53712200	3.86853000
C	4.75699600	-1.66997800	0.80431200
H	2.97116300	-1.96031700	-0.37262200
C	5.27306900	-1.48386400	2.08602600
H	4.81832600	-1.32850400	4.18630400
H	5.42342100	-1.71866400	-0.05140400
H	6.34444600	-1.38403700	2.23031200

14

E (SMD/M06-2X/6-31G(d)) = -2931.41935724

G (SMD/M06-2X/6-31G(d)) = -2931.126979

E (SMD/M06-2X/def2-TZVP//SMD/M06-2X/6-31G(d)) = -2932.65071133

C	1.00099700	-2.00360800	1.25020400
O	0.51664900	-2.93279200	0.65198900
C	0.07849100	-0.79609000	1.60138300
N	0.74425900	-0.07974200	2.64293000
N	1.20959000	0.45310300	3.48598200
B	-0.16546400	0.14408000	-0.00376900
C	1.24709700	0.59064500	-0.71329400
C	1.24998000	0.82456800	-2.09173000
C	2.44072200	0.93300300	-0.09179200
C	2.34900100	1.27541700	-2.80345400
C	3.57630900	1.37256100	-0.76292400
C	3.53280900	1.54698500	-2.13300700
C	-0.98899500	1.56174700	0.14226400
C	-1.94406200	1.99010200	-0.78551000
C	-0.61721900	2.55705000	1.04097200
C	-2.52831900	3.25105200	-0.76454300
C	-1.17441700	3.82424900	1.10753200
C	-2.15057700	4.17712800	0.19186400
C	-1.00068100	-1.00240200	-0.81668600
C	-2.37573800	-1.18162600	-0.66291200
C	-0.40941500	-1.91637100	-1.68674000
C	-3.10530800	-2.17654900	-1.29689000
C	-1.09850600	-2.92052600	-2.34962400
C	-2.46219200	-3.05552100	-2.15172100
F	2.57835300	0.83548400	1.24312600
F	4.69679700	1.62998100	-0.08695300
F	4.60292900	1.97428700	-2.79824600
F	2.27622700	1.45729300	-4.12270600
F	0.13740600	0.60898100	-2.80913700
F	0.91441900	-1.88891500	-1.91274000

F	-0.45474200	-3.76281900	-3.16131900
F	-3.14261400	-4.01834500	-2.76905300
F	-4.41845800	-2.29128700	-1.08366100
F	-3.08816900	-0.37048100	0.13122700
F	-2.35259100	1.20910700	-1.79316800
F	-3.44446300	3.57861500	-1.67677100
F	-2.70387100	5.38612700	0.22149500
F	-0.76376500	4.69943100	2.02619900
F	0.36622200	2.31323800	1.92993800
C	-1.29557800	-1.10670400	2.22125900
C	-1.91283200	-2.35123900	2.08181400
C	-1.96301100	-0.08155500	2.90117500
C	-3.19106700	-2.54992900	2.60170900
H	-1.42396000	-3.15639900	1.55107000
C	-3.23134000	-0.29278200	3.42803000
H	-1.50059900	0.89459600	3.02558000
C	-3.85483400	-1.52848900	3.27300100
H	-3.66498300	-3.51852300	2.47566500
H	-3.73229700	0.51516500	3.95157400
H	-4.85026600	-1.69264400	3.67345300
C	2.47238700	-1.96540800	1.53074400
C	2.99693800	-1.93356300	2.82564800
C	3.32601700	-2.03661000	0.42515600
C	4.37705100	-1.93040300	3.00775500
H	2.34108300	-1.94505200	3.69207200
C	4.70346500	-2.00444100	0.61510600
H	2.90358500	-2.09401800	-0.57352200
C	5.22888600	-1.94544000	1.90513700
H	4.78488700	-1.91491300	4.01333600
H	5.36601300	-2.03333500	-0.24431500
H	6.30424900	-1.92423700	2.05200100

TS_{14}

E (SMD/M06-2X/6-31G(d)) = -2931.40859154

G (SMD/M06-2X/6-31G(d)) = -2931.118591

E (SMD/M06-2X/def2-TZVP//SMD/M06-2X/6-31G(d)) = -2932.64054977

C	0.95024500	-2.10293500	1.06099700
O	0.47235900	-3.01469100	0.42251100
C	-0.00207100	-0.92622000	1.37041400
N	0.92397100	-0.15515800	2.78694600
N	1.23808700	0.31507200	3.72662000
B	-0.17149200	0.13166700	0.02077400
C	1.22403100	0.63036300	-0.69333300
C	1.18547300	0.98712900	-2.04393400
C	2.43725000	0.91238100	-0.07782500
C	2.26290600	1.49869700	-2.74703900
C	3.55182400	1.41224300	-0.74342000

C	3.46678800	1.70949600	-2.09030300
C	-0.99436300	1.54503700	0.22613400
C	-1.98767000	2.02040100	-0.63347600
C	-0.55351900	2.50107100	1.13817200
C	-2.54528100	3.29102800	-0.53692000
C	-1.07942700	3.77522300	1.27626800
C	-2.09677700	4.17648000	0.42658800
C	-1.02154900	-0.96712200	-0.88230700
C	-2.39031700	-1.17662400	-0.71330100
C	-0.43776800	-1.79288300	-1.84166800
C	-3.12659200	-2.10760400	-1.43121700
C	-1.13468100	-2.73021400	-2.58819500
C	-2.49427100	-2.89227700	-2.38066100
F	2.61846100	0.69730300	1.23652900
F	4.69337100	1.61298100	-0.08266300
F	4.51711700	2.19707400	-2.74642700
F	2.15125200	1.79752500	-4.04244800
F	0.04797000	0.83652200	-2.74002400
F	0.88256000	-1.74115500	-2.07859400
F	-0.50247100	-3.48593500	-3.48842800
F	-3.18182500	-3.79044800	-3.08073800
F	-4.43690800	-2.24509300	-1.21711400
F	-3.08889700	-0.44962400	0.16900300
F	-2.46423400	1.28436600	-1.64555300
F	-3.50318000	3.66724500	-1.38568900
F	-2.62245700	5.39444600	0.52616900
F	-0.60449700	4.61217000	2.19990900
F	0.46567300	2.20021800	1.96601200
C	-1.23941100	-1.23965000	2.16237200
C	-1.91288500	-0.19837800	2.82045700
C	-1.77346500	-2.53336200	2.20702100
C	-3.09294400	-0.44381100	3.50539800
H	-1.49724600	0.80401600	2.82249400
C	-2.97720000	-2.76500400	2.86867700
H	-1.28296800	-3.35192100	1.69728600
C	-3.63720000	-1.72832200	3.51986200
H	-3.59254000	0.37008100	4.02049200
H	-3.39211800	-3.76774900	2.87642300
H	-4.57017000	-1.91828300	4.04129700
C	2.41801200	-2.09641600	1.36301200
C	2.93594800	-2.17797700	2.65709800
C	3.27654400	-2.10466500	0.25847700
C	4.31469200	-2.22052800	2.84461400
H	2.27360400	-2.23637300	3.51562300
C	4.65330800	-2.11785700	0.45544900
H	2.85989400	-2.07756700	-0.74398300
C	5.17283900	-2.17033200	1.74806400

H	4.71659600	-2.29269800	3.85015200
H	5.31935200	-2.09653100	-0.40150800
H	6.24755400	-2.18576700	1.90045200

15

E (SMD/M06-2X/6-31G(d)) = -2931.46389705

G (SMD/M06-2X/6-31G(d)) = -2931.176424

E (SMD/M06-2X/def2-TZVP//SMD/M06-2X/6-31G(d)) = -2932.70168328

C	1.69060700	-0.82427900	0.40187200
C	2.47930500	-1.98904300	0.37399500
O	0.42935200	-0.98385700	0.27057900
N	1.74494800	-3.09244900	0.11525300
N	1.10649200	-3.97772000	-0.10029300
B	-0.72102500	-0.01065700	-0.06347900
C	-1.81432000	-0.97819300	-0.80913100
C	-2.56529100	-0.63338900	-1.92725100
C	-2.11318400	-2.22642300	-0.26667600
C	-3.52117400	-1.46891100	-2.49233900
C	-3.05497600	-3.09306300	-0.80214600
C	-3.76612800	-2.70955200	-1.92827100
C	-0.10992200	1.15524200	-1.02261600
C	0.05659500	2.49220900	-0.68481800
C	0.42413300	0.79404300	-2.25624700
C	0.71753600	3.40697600	-1.49670700
C	1.07605900	1.67374400	-3.10510200
C	1.22589500	2.99713500	-2.71742200
C	-1.44809800	0.48124100	1.30861800
C	-1.20841500	-0.00247600	2.58673100
C	-2.53317600	1.34637200	1.20190200
C	-1.96230000	0.36906100	3.69551700
C	-3.31206400	1.74293300	2.27498200
C	-3.02356100	1.24288300	3.53799600
F	-1.48869500	-2.64830300	0.84736300
F	-3.29076800	-4.28022900	-0.23804500
F	-4.67829600	-3.52319000	-2.45825900
F	-4.20466500	-1.08483800	-3.57310500
F	-2.39743500	0.55082800	-2.53500800
F	-2.85343900	1.83947800	-0.00594900
F	-4.33459900	2.58454100	2.11073600
F	-3.75843500	1.60731400	4.58772400
F	-1.67284200	-0.11603000	4.90530800
F	-0.18798100	-0.84583900	2.83086900
F	-0.37738900	2.96925800	0.49091800
F	0.88359900	4.66975300	-1.09768700
F	1.86671600	3.85968800	-3.50416600
F	1.57190300	1.26112300	-4.27405100
F	0.34713000	-0.48582900	-2.66205300

C	3.92784700	-2.17938000	0.61532400
C	4.67215200	-3.01375500	-0.22429500
C	4.54878700	-1.53626300	1.69137800
C	6.03059800	-3.20236900	0.01180600
H	4.19085600	-3.50878700	-1.06401200
C	5.91118500	-1.71346800	1.90780600
H	3.96516500	-0.90459100	2.35424900
C	6.65301600	-2.54794100	1.07264900
H	6.60332500	-3.85334200	-0.64110100
H	6.39067000	-1.20874100	2.74050300
H	7.71434000	-2.69010200	1.25095500
C	2.32259000	0.50237300	0.57718300
C	3.28768600	0.93119200	-0.34082800
C	1.91899700	1.33013500	1.62789000
C	3.81402100	2.21302400	-0.22736100
H	3.59857600	0.27432600	-1.14863200
C	2.47178900	2.60140600	1.74750900
H	1.19388700	0.97378200	2.35103900
C	3.40481500	3.04807400	0.81367500
H	4.54283400	2.56162100	-0.95219700
H	2.16250100	3.24635800	2.56357400
H	3.81880600	4.04803600	0.89856400

TS$_{15\text{-}16}$
E (SMD/M06-2X/6-31G(d)) = -2931.4287877
G (SMD/M06-2X/6-31G(d)) = -2931.145119
E (SMD/M06-2X/def2-TZVP//SMD/M06-2X/6-31G(d)) = -2932.6677477

C	1.77050300	-0.33610200	-0.69575100
C	2.74696100	-1.15113900	-1.07339000
O	0.50160900	-0.51391800	-0.95304100
N	1.68618400	-2.61511000	-1.83596200
N	1.27355000	-3.56039000	-2.21030700
B	-0.67602900	0.02987900	-0.16014100
C	-1.98291500	-0.52974500	-0.98487600
C	-3.10998300	0.19818400	-1.34905500
C	-2.04862300	-1.88795700	-1.28904500
C	-4.20305600	-0.35928300	-2.00258600
C	-3.11600500	-2.48522200	-1.94309200
C	-4.20586000	-1.70998200	-2.30613000
C	-0.56378200	1.66312200	-0.12470600
C	-0.30924100	2.45143700	0.99059500
C	-0.59087100	2.35903900	-1.33016100
C	-0.08504200	3.82220300	0.92534900
C	-0.38352600	3.72412800	-1.44301500
C	-0.12167500	4.46435700	-0.29949600
C	-0.79856900	-0.67925300	1.31475200
C	-0.05814300	-1.75241200	1.78762800

C	-1.85674400	-0.29886500	2.13624900
C	-0.30972500	-2.38118400	3.00329800
C	-2.14912300	-0.89387100	3.35106200
C	-1.36404200	-1.95199000	3.78912500
F	-1.05033900	-2.71128600	-0.92017000
F	-3.11065000	-3.79424200	-2.20990200
F	-5.24626300	-2.25976000	-2.93196700
F	-5.25180100	0.39749500	-2.33682500
F	-3.20770300	1.50965300	-1.08259300
F	-2.65236300	0.71199700	1.74647600
F	-3.17370700	-0.47363800	4.09711900
F	-1.62331500	-2.54433100	4.95477500
F	0.45292400	-3.40037900	3.41008900
F	0.98660600	-2.24278900	1.08971700
F	-0.22319100	1.91674000	2.21914600
F	0.20097800	4.51444200	2.03152900
F	0.10439400	5.77559100	-0.38286200
F	-0.42216700	4.32986800	-2.63357700
F	-0.82571600	1.69492100	-2.47465800
C	4.10492000	-1.54264200	-1.17186300
C	4.89929200	-1.08373300	-2.24197700
C	4.65556500	-2.38123100	-0.18051100
C	6.23552500	-1.44722000	-2.30402500
H	4.45388500	-0.44307200	-2.99662600
C	5.99023600	-2.74460100	-0.25752200
H	4.02014200	-2.72505500	0.63051700
C	6.77365300	-2.27606900	-1.31628100
H	6.86009900	-1.09253400	-3.11683600
H	6.42633400	-3.38622000	0.50052300
H	7.81997300	-2.56129200	-1.37185800
C	2.33048200	0.84110600	0.05298300
C	2.65066300	2.01144600	-0.64237700
C	2.49214400	0.76232700	1.43612400
C	3.08878500	3.12479400	0.06467200
H	2.53702400	2.04585300	-1.72247600
C	2.92589100	1.88546800	2.13735600
H	2.26847500	-0.16566600	1.95454200
C	3.21464900	3.06488700	1.45486300
H	3.32678500	4.04213100	-0.46535800
H	3.03641700	1.83752000	3.21598200
H	3.54677500	3.94027100	2.00465100

16
E (SMD/M06-2X/6-31G(d)) = -2821.95195238
G (SMD/M06-2X/6-31G(d)) = -2821.673523
E (SMD/M06-2X/def2-TZVP//SMD/M06-2X/6-31G(d)) = -2823.14668424

C	1.90715200	-0.24771000	-0.70006200

C	2.97650400	-0.87651300	-1.11447600
O	0.65850600	-0.53833300	-0.98121900
B	-0.55867100	-0.06820500	-0.21464700
C	-1.80994200	-0.70311000	-1.07168600
C	-2.96489300	-0.04139600	-1.47172200
C	-1.78423500	-2.06267900	-1.37784000
C	-4.00305700	-0.66005200	-2.15868700
C	-2.79596900	-2.71902200	-2.06382500
C	-3.91760300	-2.00829700	-2.46058500
C	-0.55058900	1.57110200	-0.17071200
C	-0.39385100	2.37070600	0.95447100
C	-0.57296600	2.26825000	-1.37580300
C	-0.25449700	3.75331800	0.89952500
C	-0.44635000	3.64400100	-1.47927700
C	-0.27931300	4.39577200	-0.32530900
C	-0.68132600	-0.77869200	1.26150000
C	0.13266200	-1.77466800	1.77848800
C	-1.79895600	-0.48392600	2.03820500
C	-0.11028700	-2.41248300	2.99126600
C	-2.08631100	-1.09153200	3.24798700
C	-1.22982400	-2.07277800	3.72916600
F	-0.75048000	-2.82477000	-0.98378900
F	-2.70705300	-4.02546000	-2.33122300
F	-4.90488000	-2.61740700	-3.11770100
F	-5.08343900	0.03499400	-2.52663300
F	-3.14519800	1.26268300	-1.21022700
F	-2.65894600	0.45678100	1.61034000
F	-3.17100400	-0.75405600	3.95017200
F	-1.48183900	-2.67478900	4.89169600
F	0.72497600	-3.35342000	3.44245600
F	1.25088300	-2.16594600	1.13426800
F	-0.32474700	1.83993400	2.18617500
F	-0.05980600	4.45904300	2.01724200
F	-0.13312600	5.71905400	-0.39863800
F	-0.47443900	4.25034500	-2.66994600
F	-0.72304400	1.59471200	-2.52894100
C	4.15905600	-1.52826000	-1.35937700
C	4.92245300	-1.23262300	-2.52548700
C	4.60831200	-2.52315200	-0.44201200
C	6.09853200	-1.91439700	-2.75795500
H	4.55996600	-0.47280300	-3.21017400
C	5.78635100	-3.19571800	-0.69157800
H	4.00466100	-2.73179400	0.43575800
C	6.52282000	-2.88814200	-1.84331200
H	6.69303200	-1.70286500	-3.63959900
H	6.14285000	-3.95616800	-0.00598000
H	7.44989300	-3.42109200	-2.03392600

C	2.37808100	0.92847300	0.11947900
C	2.66693800	2.13936000	-0.52242200
C	2.48807600	0.80968100	1.50750200
C	3.00257500	3.25155100	0.23935200
H	2.60385900	2.20176100	-1.60513600
C	2.81930000	1.93129000	2.26230200
H	2.30576500	-0.14803500	1.98473100
C	3.06581900	3.14972600	1.63143300
H	3.21088200	4.19885100	-0.24856200
H	2.88564200	1.85312300	3.34271600
H	3.32002700	4.02290900	2.22458800

$TS_{16\text{-}17}$
E (SMD/M06-2X/6-31G(d)) = -2821.95162137
G (SMD/M06-2X/6-31G(d)) = -2821.672779
E (SMD/M06-2X/def2-TZVP//SMD/M06-2X/6-31G(d)) = -2823.14607734

C	1.64380700	-0.51532600	-1.07399200
C	2.80693200	-0.93964500	-1.34046500
O	0.37784800	-0.47178500	-1.27121500
B	-0.68070300	-0.02225600	-0.25832600
C	-2.07947200	-0.18434900	-1.09569300
C	-3.10144700	0.75203600	-1.19477900
C	-2.34089100	-1.40282400	-1.71940100
C	-4.28234000	0.52215700	-1.89081300
C	-3.50370400	-1.67336800	-2.42556300
C	-4.48450400	-0.69785500	-2.51326200
C	-0.32706300	1.51662700	0.16985400
C	0.03214100	1.96842900	1.43336200
C	-0.29264100	2.49259700	-0.82459400
C	0.39548800	3.28442600	1.70182500
C	0.05675200	3.81478900	-0.60260200
C	0.40928200	4.21445900	0.67845100
C	-0.81658900	-1.06317500	0.99868600
C	-0.16814200	-2.27793800	1.16055000
C	-1.79955700	-0.79894600	1.94968300
C	-0.44074000	-3.15949600	2.20235700
C	-2.10862500	-1.64360500	3.00114100
C	-1.42054100	-2.84348000	3.12594300
F	-1.44998200	-2.40465400	-1.63782500
F	-3.69227000	-2.86112600	-3.00669600
F	-5.61166200	-0.93410700	-3.18366400
F	-5.22500300	1.46604000	-1.95953200
F	-2.99957300	1.95511000	-0.60910000
F	-2.49338000	0.34936600	1.86495100
F	-3.05608700	-1.32569900	3.88625600
F	-1.69836400	-3.67624200	4.12852500
F	0.23195400	-4.30859400	2.31038000

F	0.80356000	-2.66725900	0.31262900
F	0.08294300	1.13810700	2.48732900
F	0.77924100	3.64040300	2.93080900
F	0.77447700	5.47396700	0.91401300
F	0.06610800	4.70070900	-1.60178500
F	-0.62733300	2.16577200	-2.08288700
C	4.12670800	-1.42113800	-1.64278900
C	4.55814800	-2.62994100	-1.07618200
C	4.97014800	-0.69402800	-2.49590400
C	5.83046000	-3.10705300	-1.36597800
H	3.88823100	-3.17987700	-0.42201000
C	6.24123600	-1.17821600	-2.77488600
H	4.62190000	0.23932000	-2.92744000
C	6.66986400	-2.38119500	-2.21101000
H	6.16814400	-4.04314300	-0.93344300
H	6.89893800	-0.62054100	-3.43359800
H	7.66475300	-2.75498300	-2.43295200
C	2.56529100	0.30708800	-0.08900400
C	2.91573300	1.62441800	-0.45428700
C	2.68697500	-0.13390200	1.24276300
C	3.36349800	2.49800800	0.52118100
H	2.82376300	1.93073800	-1.49277500
C	3.13390200	0.75230700	2.21100800
H	2.43465200	-1.16152600	1.48725400
C	3.46712300	2.05971100	1.84786500
H	3.63247600	3.51692700	0.26261600
H	3.22540400	0.43254100	3.24323500
H	3.81743500	2.75055500	2.60903800

17

E (SMD/M06-2X/6-31G(d)) = -614.465147024

G (SMD/M06-2X/6-31G(d)) = -614.306466

E (SMD/M06-2X/def2-TZVP//SMD/M06-2X/6-31G(d)) = -614.697058988

C	0.00002800	2.15567600	0.00015800
C	-0.00005500	0.82852200	0.00024500
O	0.00025500	3.32003600	0.00001400
C	1.31443600	0.13602900	0.03176200
C	2.44223100	0.69719500	-0.58184400
C	1.45004200	-1.08749200	0.70190800
C	3.67761800	0.06032700	-0.51279900
H	2.34862700	1.63452100	-1.12526800
C	2.68290800	-1.73093200	0.75102800
H	0.58690300	-1.53194300	1.18879700
C	3.80297400	-1.15947600	0.14886400
H	4.54052600	0.51339400	-0.99174400
H	2.76921200	-2.67998800	1.27188200
H	4.76398700	-1.66251300	0.19239400

C	-1.31454300	0.13610500	-0.03159700
C	-2.44252900	0.69734400	0.58154300
C	-1.44986700	-1.08758600	-0.70147300
C	-3.67787700	0.06038800	0.51230100
H	-2.34921500	1.63483700	1.12474800
C	-2.68269700	-1.73106000	-0.75087600
H	-0.58655400	-1.53215100	-1.18796200
C	-3.80298700	-1.15955100	-0.14914500
H	-4.54089600	0.51352600	0.99098000
H	-2.76878000	-2.68023100	-1.27155700
H	-4.76394000	-1.66268300	-0.19283200

18

E (SMD/M06-2X/6-31G(d)) = -669.824876067

G (SMD/M06-2X/6-31G(d)) = -669.648694

E (SMD/M06-2X/def2-TZVP//SMD/M06-2X/6-31G(d)) = -670.074915671

N	-0.07207000	-0.25071900	0.00000200
C	-1.16930600	-0.91399300	0.00002100
H	-1.16358000	-2.00998400	0.00003000
C	-2.48170400	-0.26774400	0.00001000
C	-3.62549600	-1.07479200	0.00001600
C	-2.61401000	1.12856900	-0.00000700
C	-4.89077400	-0.49590200	0.00000400
H	-3.51461600	-2.15624500	0.00002900
C	-3.87706200	1.70330900	-0.00001800
H	-1.72019500	1.74459700	-0.00001100
C	-5.01539100	0.89199900	-0.00001300
H	-5.77622200	-1.12360100	0.00000800
H	-3.98154400	2.78378900	-0.00003200
H	-6.00177500	1.34621900	-0.00002300
C	1.11401500	-1.04287000	-0.00000300
O	1.12059700	-2.25918200	-0.00001600
C	2.37692400	-0.25137600	0.00000000
C	2.37294800	1.14702900	0.00001400
C	3.59312200	-0.94451500	-0.00001200
C	3.57849200	1.84366400	0.00001400
H	1.42807300	1.67807300	0.00002200
C	4.79393100	-0.24665600	-0.00001100
H	3.57838700	-2.02980800	-0.00002200
C	4.78685700	1.14945900	0.00000200
H	3.57479500	2.92929800	0.00002400
H	5.73565000	-0.78668500	-0.00002100
H	5.72545700	1.69574300	0.00000200

$TS_{17\text{-}19}$

E (SMD/M06-2X/6-31G(d)) = -1284.28070958

G (SMD/M06-2X/6-31G(d)) = -1283.923545

E (SMD/M06-2X/def2-TZVP//SMD/M06-2X/6-31G(d)) = -1284.7584264

C	0.08469100	0.32125600	1.55178600
C	0.79940600	1.17764800	0.74635200
O	0.25440000	-0.49096700	2.42183900
C	0.15863100	2.15420700	-0.16430700
C	-0.90739000	2.97214100	0.24278500
C	0.62160200	2.29801700	-1.48408700
C	-1.50631700	3.86818600	-0.63914400
H	-1.26194800	2.90544500	1.26689800
C	0.03329000	3.20271800	-2.36316900
H	1.45987900	1.69043800	-1.81827300
C	-1.04021200	3.98923300	-1.94689600
H	-2.33178700	4.48571500	-0.29624500
H	0.41119600	3.28896500	-3.37802100
H	-1.50231700	4.69388700	-2.63173200
C	2.27559100	1.11998400	0.84926000
C	3.05946900	2.24757500	0.54113400
C	2.95508300	-0.05303400	1.23022900
C	4.44841100	2.20559900	0.60949000
H	2.56953000	3.17218800	0.25002000
C	4.34420600	-0.09146500	1.29924400
H	2.38481400	-0.94378700	1.47283700
C	5.10386800	1.03531600	0.98883200
H	5.02063100	3.09767800	0.36958300
H	4.83521800	-1.01633100	1.59019300
H	6.18790700	1.00246300	1.04197200
N	-1.14129900	-1.47243500	-0.07179600
C	-0.11088000	-1.22347800	-0.80267900
H	-0.06323000	-0.30504200	-1.39412100
C	1.00539800	-2.14796500	-0.91717800
C	2.06354900	-1.82001500	-1.77395400
C	1.07891400	-3.29273000	-0.10900800
C	3.18704300	-2.63717400	-1.83199000
H	2.00129100	-0.92119900	-2.38226500
C	2.20287400	-4.10345800	-0.16970600
H	0.25799100	-3.52270000	0.56393200
C	3.25487600	-3.77570300	-1.03038500
H	4.00994300	-2.38434600	-2.49248600
H	2.26813800	-4.98857800	0.45484000
H	4.13431300	-4.41143000	-1.07068300
C	-1.96616900	-0.57139800	0.46126400
O	-1.51960600	0.38809700	1.17383400
C	-3.42227600	-0.73485800	0.34433300
C	-3.94753400	-1.82030300	-0.36638100
C	-4.27269700	0.21544500	0.92162200
C	-5.32414000	-1.95456300	-0.49493100
H	-3.27631000	-2.55329500	-0.80294300

C	-5.64800500	0.07513600	0.78670900
H	-3.85075100	1.05480700	1.46442100
C	-6.17233100	-1.00814000	0.08066800
H	-5.73694500	-2.79669100	-1.04064100
H	-6.31251500	0.80873800	1.23133600
H	-7.24801400	-1.11597100	-0.02081200

19

E (SMD/M06-2X/6-31G(d)) = -1284.34896984

G (SMD/M06-2X/6-31G(d)) = -1283.983889

E (SMD/M06-2X/def2-TZVP//SMD/M06-2X/6-31G(d)) = -1284.82122034

C	-0.01018500	0.21414300	-1.43035700
C	-0.71482300	0.60343600	-0.13898700
O	-0.41986300	0.33925500	-2.54925400
C	-0.12928400	1.99463600	0.19010100
C	-0.31808700	3.01743400	-0.74865900
C	0.54503300	2.28676600	1.37746800
C	0.15307300	4.30231600	-0.50634100
H	-0.84689400	2.80194700	-1.67396300
C	1.01346000	3.57875500	1.62130800
H	0.72248500	1.51788600	2.12161200
C	0.82147000	4.58804300	0.68391600
H	-0.00392400	5.08083000	-1.24663900
H	1.53353000	3.78891800	2.55099000
H	1.18969100	5.59102100	0.87699300
C	-2.23378800	0.67593400	-0.28523000
C	-2.95473700	1.49743200	0.58687900
C	-2.94088300	-0.14788200	-1.16806900
C	-4.34791800	1.50352000	0.57482300
H	-2.42449200	2.13905000	1.28527300
C	-4.33285400	-0.13483800	-1.18620300
H	-2.40898300	-0.81292200	-1.83923200
C	-5.04264000	0.68965200	-0.31581100
H	-4.88701100	2.14865400	1.26212800
H	-4.86259000	-0.77913500	-1.88152400
H	-6.12842300	0.69590800	-0.33092700
N	1.09531100	-0.66465000	1.02887300
C	-0.34241000	-0.46275000	0.94325300
H	-0.67859500	-0.05682700	1.90299400
C	-1.05979700	-1.79101100	0.74377100
C	-2.22992200	-2.04728400	1.46227300
C	-0.60682300	-2.74026700	-0.17539300
C	-2.94592300	-3.22309500	1.25675900
H	-2.58895300	-1.30998600	2.17644000
C	-1.32387200	-3.91714300	-0.38413400
H	0.31045700	-2.57576500	-0.73517000
C	-2.49549900	-4.16051500	0.32892600

H	-3.85498700	-3.40666000	1.82156200
H	-0.96136800	-4.64552100	-1.10319900
H	-3.05174500	-5.07869700	0.16608500
C	1.78604900	-0.52943000	-0.02037800
O	1.27521200	-0.23475100	-1.27223700
C	3.25498200	-0.70645800	-0.05600400
C	3.92266500	-1.05790200	1.12287600
C	3.97776200	-0.52247500	-1.23823500
C	5.30162500	-1.22255200	1.11670400
H	3.35009200	-1.19841100	2.03364600
C	5.36038100	-0.68844400	-1.23734000
H	3.46280900	-0.25079800	-2.15284100
C	6.02344300	-1.03778900	-0.06356100
H	5.81596500	-1.49535500	2.03294000
H	5.91845400	-0.54388200	-2.15708500
H	7.10153900	-1.16712600	-0.06639100
